# Synthesis, Structure–Activity Relationships, and Parasitological Profiling of Brussonol Derivatives as New *Plasmodium* *falciparum* Inhibitors

**DOI:** 10.3390/ph15070814

**Published:** 2022-06-30

**Authors:** Camila S. Barbosa, Anees Ahmad, Sarah El Chamy Maluf, Igor M. R. Moura, Guilherme E. Souza, Giovanna A. H. Guerra, Roberto R. Moraes Barros, Marcos L. Gazarini, Anna C. C. Aguiar, Antonio C. B. Burtoloso, Rafael V. C. Guido

**Affiliations:** 1 São Carlos Institute of Physics, University of São Paulo, Av. João Dagnone, 1100, Santa Angelina, São Carlos 13563-120, Brazil; camilasbarbosa@ifsc.usp.br (C.S.B.); sarahmaluf@usp.br (S.E.C.M.); igormrmoura@usp.br (I.M.R.M.); guilherme.eduardo.souza@usp.br (G.E.S.); 2 São Carlos Institute of Chemistry, University of São Paulo, Av. João Dagnone, 1100, Santa Angelina, São Carlos 13563-120, Brazil; anees_chemist@yahoo.com; 3 Department of Microbiology, Immunology and Parasitology, Escola Paulista de Medicina, Federal University of São Paulo, São Paulo 04023-062, Brazil; giovanna.abreu31@gmail.com (G.A.H.G.); moraes.barros@unifesp.br (R.R.M.B.); 4 Department of Biosciences, Federal University of São Paulo, Rua Silva Jardim, 136, Santos 11015-020, Brazil; marcos.gazarini@unifesp.br

**Keywords:** malaria, icetexane diterpenoids, antimalarials, resistance, *Plasmodium* *falciparum*

## Abstract

Malaria is a parasitic disease caused by protozoan parasites from the genus *Plasmodium*. *Plasmodium falciparum* is the most prevalent species worldwide and the causative agent of severe malaria. The spread of resistance to the currently available antimalarial therapy is a major concern. Therefore, it is imperative to discover and develop new antimalarial drugs, which not only treat the disease but also control the emerging resistance. Brussonol is an icetexane derivative and a member of a family of diterpenoids that have been isolated from several terrestrial plants. Here, the synthesis and antiplasmodial profiling of a series of brussonol derivatives are reported. The compounds showed inhibitory activities in the low micromolar range against a panel of sensitive and resistant *P. falciparum* strains (IC_50_s = 5–16 μM). Moreover, brussonol showed fast-acting in vitro inhibition and an additive inhibitory behavior when combined with the antimalarial artesunate (FIC_index_~1). The mode of action investigation indicated that brussonol increased the cytosolic calcium levels within the parasite. Hence, the discovery of brussonol as a new scaffold endowed with antiplasmodial activity will enable us to design derivatives with improved properties to deliver new lead candidates for malaria.

## 1. Introduction

Malaria is a parasitic disease caused by protozoan parasites from the genus *Plasmodium* spp. *Plasmodium falciparum* is the most prevalent species worldwide [1] and the causative agent of severe malaria. Despite the reduction in malaria incidence and mortality over the last 20 years, the disease affects millions of people annually. In 2020, the World Health Organization (WHO) estimated the occurrence of 241 million cases and over 600 thousand deaths globally. Children aged under 5 years were the most vulnerable group affected by the disease. In 2020, they accounted for 77% of all malaria deaths worldwide [1]. The spread of resistance to the currently available antimalarial therapy is a major concern, especially the recent evidence of the independent emergence of artemisinin partial resistance in the WHO African Region (World Health Organization, 2020). A WHO protocol to monitor antimalarial drug efficacy, as well as surveillance for resistance markers, are common routines used in endemic countries to readapt the protocol for the management and control of malaria cases [2]. All progress achieved over the past two decades in controlling, managing, and preventing malaria cases can be lost if the spread of artemisinin resistance outruns the speed of new antimalarials delivery [2]. Therefore, it is imperative to discover and develop new antimalarial drugs, which would not only treat symptoms, but also control the emerging resistance, and contribute toward the elimination and eradication of the disease. 

**Figure 1:**
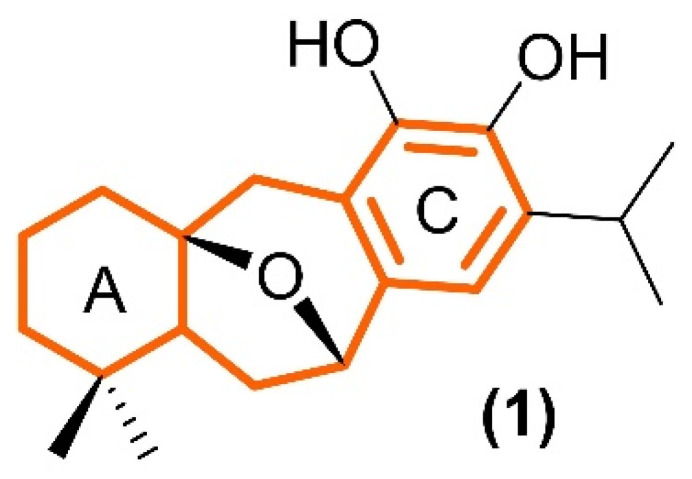
Molecular structure of brussonol (**1**). The structure in orange indicates the 6-7-6 tricyclic framework characteristic of icetexanes diterpenes.

A valuable source for the discovery of new potential drug candidates relies on the synthesis of active compounds inspired by natural products. Plant extracts have been used by humans to treat diseases since ancient times, and their chemical complexity and diversity are attractive features to medicinal chemists [3]. The icetexanes are a family of diterpenoids that have been isolated from different parts of terrestrial plants. They are found in several genera, such as *Salvia*, *Perovskia*, and *Dracocephalum* [4,5,6]. These compounds have a 6-7-6 tricyclic framework, which is a key feature of the icetexane skeleton (Figure 1). Antibacterial [4,7], antiprotozoal [6,8,9,10], and antiproliferative activities [11] have been reported for some diterpenes. In this work, we investigated brussonol and synthetic derivatives as *P. falciparum* inhibitors (Figure 1). In this sense, we conducted extensive synthetic and biological profiling efforts to assess the antiplasmodial properties of the brussonol series. Our findings indicated that the natural compound derivates are new antiplasmodial hit candidates.

## 2. Results

### 2.1. Synthesis of Brussonol and Derivatives

**Figure 2:**
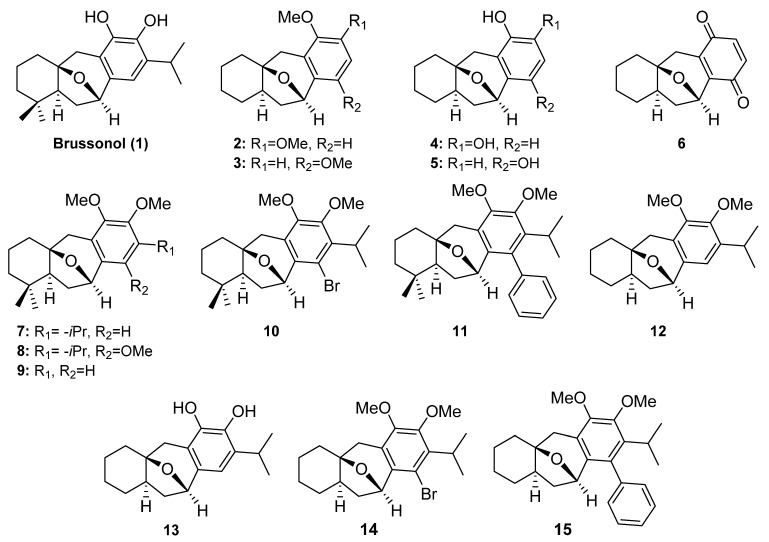
Design of brussonol analogs as new *Plasmodium falciparum* inhibitors.

In our initial structure–activity relationship (SAR) campaign, the design and synthesis of natural product brussonol (**1**) and 14 analogs were executed (Figure 2). The design strategy involved modifications to the aromatic and six-membered cyclic ring of the brussonol (**1**) skeleton to determine the necessary motifs required for antiplasmodial activity.

**Scheme 1:**
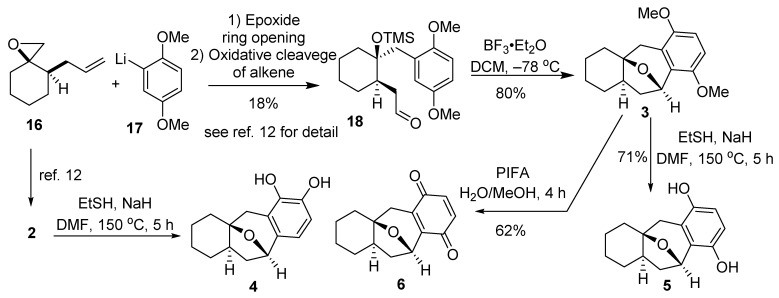
Synthetic route for the synthesis of brussonol analogs.

**Scheme 2:**
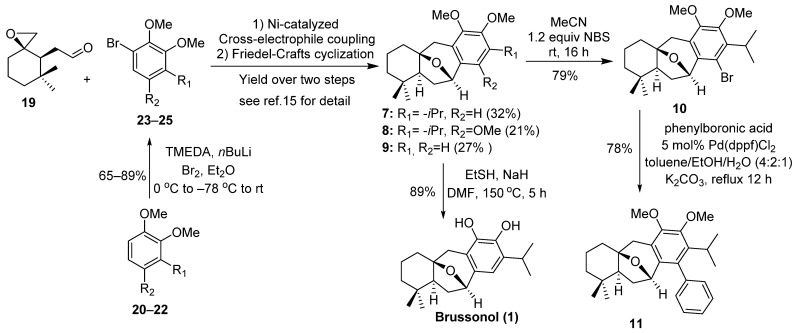
Synthesis of brussonol (**1**) and analogs.

**Scheme 3:**
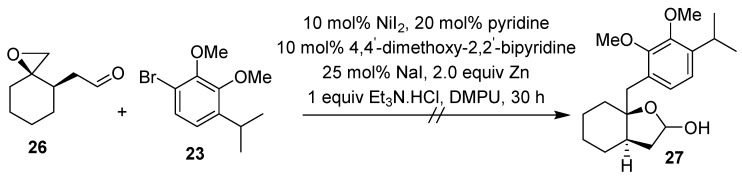
Cross-electrophile coupling between aryl bromide and epoxy-aldehyde.

Three synthetic routes were explored to obtain desired brussonol analogs **1**–**15** as new antimalarial agents (Scheme 1, Scheme 2 and Scheme 3). Target compounds **2** with 1,2-disubstitution on the aromatic ring were synthesized according to the protocol reported previously by our group [12]. Thus, following the same synthetic route, the synthesis of 1,4-disubstituted analog **3** was accomplished (Scheme 1). After preparing epoxide **16**, it was subjected to a regioselective epoxide ring-opening reaction mediated by the *ortho*-lithiated nucleophile **17**, derived from 1,4-dimethoxybenzene. In the next step, oxidative cleavage of the terminal olefin into aldehydes was employed using Lemieux–Johnson oxidation conditions. Marson-type Friedel–Crafts acylation of aldehyde **18** using BF_3_.Et_2_O as the Lewis acid afforded the synthesis of tricyclic moiety **3** in 80% yield. Finally, the hydroquinone type brussonol analogs **4** and **5** were prepared from respective tricyclic moieties **2** and **3** via demethylation protocol using EtSH, NaH, and DMF as solvents in good yields [13]. Similarly, *p*-quinone **6**, a komaroviquinone-type derivative, was synthesized from **3** via oxidative demethylation of phenol ethers using a hypervalent iodine (III) reagent (PIFA) in 62% yield [14].

Recently, we reported a short and stereoselective synthesis of (±)-brussonol and (±)-komaroviquinone, via a Ni-catalyzed epoxide ring-opening approach in the presence of aryl halides [15]. Using the same protocol, we successfully prepared brussonol (**1**) and several other analogs (**7**–**9**, **10**, and **11**) in good quantities (Scheme 2). The coupling fragments, such as epoxide **19** and aryl bromides (**23**–**25**), were prepared as previously described in the literature [15,16,17,18]. The hemiacetal intermediates obtained via a regioselective cross-electrophile coupling of aryl bromides **23**–**25** and epoxy-aldehyde **19** were readily subjected to Friedel–Crafts cyclization reaction to afford the formation of the tricyclic compounds (icetexane diterpenes skeleton) **7**–**9** in 21–32% yields. The subsequent demethylation of compound **7** furnished brussonol (**1**) in an 89% yield. After optimization studies, we obtained the bromine-substituted analog **10** from compound **7** in the presence N-Bromosuccinimide (NBS) in acetonitrile as the solvent in 79% yield [19]. At this stage, we were set for the Suzuki cross-coupling reaction as we had the bromine-substituted analog **10** in hand. The Suzuki cross-coupling reaction between substrate **10** and phenylboronic acid catalyzed by Pd(dppf)Cl_2_, in the presence of K_2_CO_3_ as the base, provided our desired coupled product **11** in 79% yield when the reaction was allowed to run for 24 h [20].

Similarly, we also planned the synthesis of isopropyl containing dimethoxy analog, but this time without the geminal dimethyl on the cyclohexane ring. Unfortunately, we were not able to isolate the desired acetal product **27** in pure form when aryl bromide **23** and epoxy-aldehyde **26** were subjected to the nickel/iodide catalyzed cross-electrophile coupling reaction (Scheme 3).

**Scheme 4:**
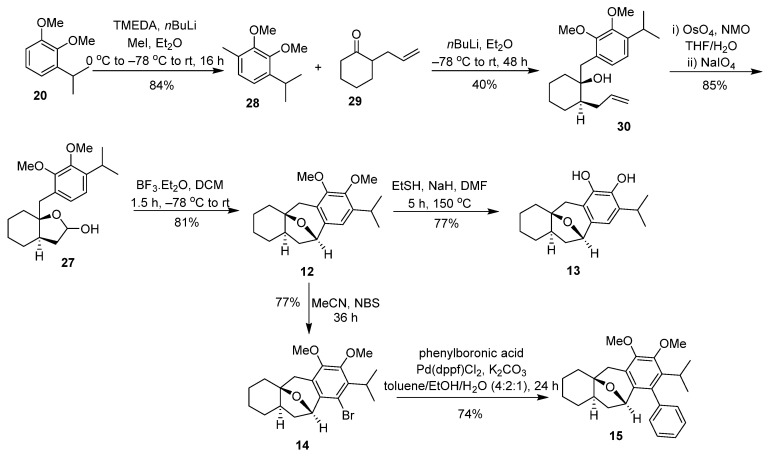
Synthesis of brussonol analogs.

To overcome the difficulties and failures associated with the preparation of our desired acetal intermediate **27**, we switched to an alternative synthetic plan reported in the literature [21]. Following the reported protocol, ortho-directed metalation in the presence of nBuLi and TMEDA in diethyl ether as a solvent, with subsequent electrophilic quench with iodomethane, provided the methylated aryl moiety **28** in 84% yield (Scheme 4). Deprotonation of the methyl group in **28**, followed by the addition of ketone **29**, gave the desired tertiary alcohol **30** in 40% yield. Oxidative cleavage of the allyl group in tertiary alcohol **30**, followed by hemiacetalization, provided acetal **27** in good yield. After successful synthesis of hemiacetal **27**, it was readily subjected to Marson-type Friedel–Crafts cyclization, which resulted in tricyclic structure **12** in 81% yield. The demethylation of compound **12** afforded **13** in 77% yield. We also successfully obtained brominated analog **14** in the presence of NBS in a 77% yield [19]. Similarly, the Suzuki cross-coupling reaction between substrate **14** and phenylboronic acid furnished the anticipated analog **15** in 74% yield [20]. With the above-given strategy, we prepared four additional synthetic analogs (**12**–**15**) in good quantities for biological activity assessment. All compounds were synthesized as a racemic mixture.

### 2.2. Brussonol and Derivatives Showed Antiplasmodial Activity and Low Cytotoxic Activity

**Figure 3:**
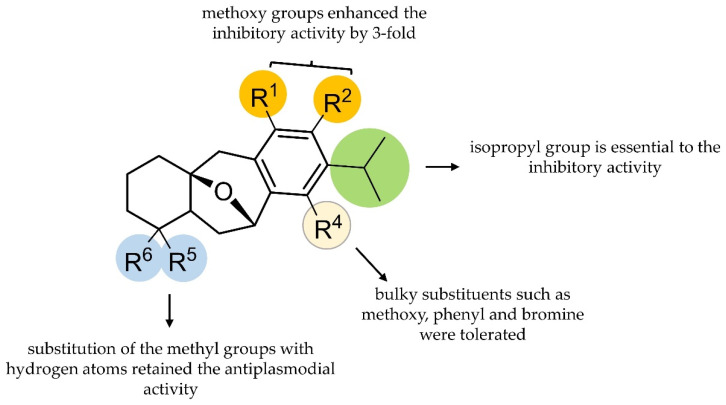
SAR summary around the icetexane diterpenoid scaffold.

The in vitro inhibitory activity against *P. falciparum* (3D7 strain), cytotoxic effect on HepG2 cells, and selectivity index (SI) of brussonol and derivatives were assessed (Table 1). The assessed IC_50_ value of brussonol was 16 µM, indicating that the icetexane diterpenoid scaffold is endowed with promising antiplasmodial activity. Because of that, we designed new analogs to investigate the SAR underlying the central molecular scaffold (Scheme 1, Scheme 2, Scheme 3 and Scheme 4 and Table 1). The SAR investigation indicated that some key structural features are crucial for antiplasmodial activity (Figure 3). For example, the removal of the isopropyl group at R^3^ in derivatives **2**–**6** and **9** abrogated the antiplasmodial activity, suggesting that the bulky substituent at R^3^ is favorable for the inhibitory activity. By contrast, the substitution of the hydroxyl substituent at R^1^ and R^2^ with methoxy groups enhanced by 3-fold the inhibitory activity (e.g., **1** vs. **7**; **12** vs. **13**). The substitution with bulkier and hydrophobic substituents, such as methoxy (**8**), bromine (**10**), and phenyl (**11**), at R^4^ was tolerated. Finally, the substitution of the methyl groups at positions R^5^ and R^6^ with hydrogen atoms retained the antiplasmodial activity (e.g., **7** vs. **12**; **10** vs. **14**; **11** vs. **15**). In sum, brussonol and derivatives showed IC_50_ values that ranged from 5 to >10 µM, and 7 out of 14 derivatives showed IC_50_ values < 10 µM, thereby suggesting that structural modifications around the icetexane diterpenoid scaffold modulate the inhibitory activity of this series.

Compounds with IC_50_ values < 10 µM had their cytotoxic effect assessed against a human hepatocellular carcinoma (HepG2) cell line. Brussonol and derivatives showed varying cytotoxic effects on HepG2 cells. For instance, brussonol showed an IC_50_ value of 67 µM against the mammalian cells (Table 1). The IC_50_s of this set of derivates ranged from >12 to >400 µM, therefore, the compounds were not generally cytotoxic, as demonstrated by the assessed SI values greater than 10 against the liver cells [22].

### 2.3. Brussonol Is a Potent Inhibitor of Resistant P. falciparum Strains

**Figure 4:**
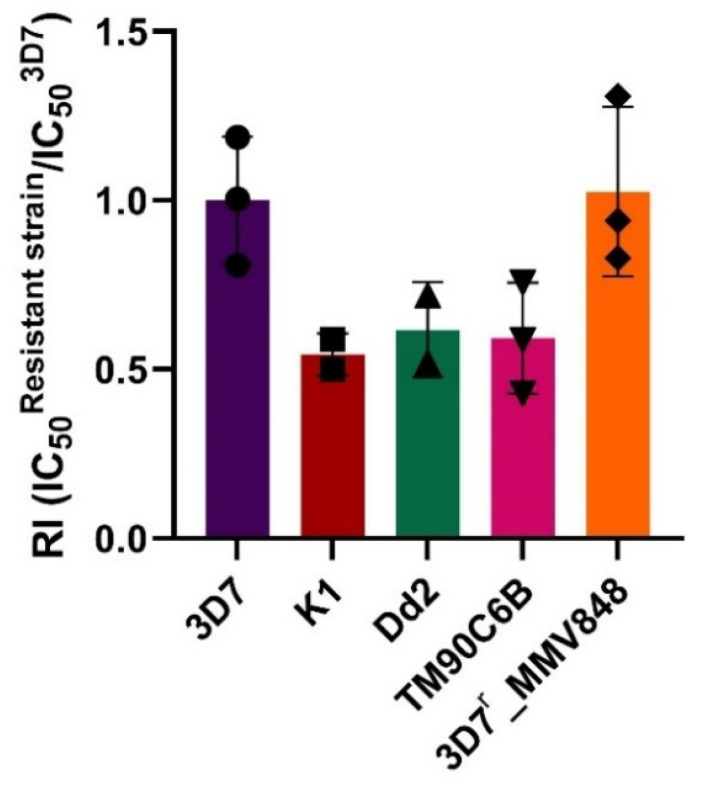
Analysis of resistance index (RI) of brussonol against a panel of sensitive and resistant *P. falciparum* strains (K1, Dd2, TM90C6B, and 3D7^r^_MMV848) relative to the sensitive strain (3D7). Plot of the fold-change in the antiplasmodial potency of brussonol against a panel of resistant strains relative to the sensitive strain. (*n* ≥ 2, mean IC_50_ ± SD).

We selected brussonol (**1**, IC_50_ = 16 µM) as a representative of the series for parasitological profiling because the molecule was our first identified hit. Thus, we first assessed the inhibitory activity of brussonol against a small panel of representative-resistant strains of *P. falciparum* (Table 2). The panel included K1 (resistant to chloroquine, sulfadoxine, pyrimethamine, and cycloguanil), Dd2 (resistant to chloroquine, sulfadoxine, pyrimethamine, mefloquine, and cycloguanil), TM90C6B (resistant to chloroquine, pyrimethamine, and atovaquone), and 3D7r-MMV848 (resistant to MMV692848) strains. As the IC_50_ values were determined for the resistant parasite strains, we determined the resistance index (RI), which corresponds to the ratio between the IC_50_ values against each resistant strain and the 3D7 strain (Figure 4). In this context, a molecule shows cross-resistance if the RI value is greater than five [23]. Brussonol showed comparable inhibitory activities between the resistant and the sensitive strains (RI < 5), thereby indicating no cross-resistance with the standard antimalarials used as inhibition control.

### 2.4. Brussonol Is a Potent P. knowlesi Inhibitor

To evaluate whether this series of brussonol derivatives show inhibitory activity against other Plasmodium species, we assessed the antiplasmodial activity of brussonol (**1**) against *P. knowlesi*, a simian parasite that causes zoonotic malaria in humans. This parasite is prevalent in Malaysia [24], and more than 2600 zoonotic cases were reported in 2020, after 3 years without notification of human malaria in this region [1]. Compound **1** showed inhibitory activity against *P. knowlesi* parasites in the low micromolar range (IC_50_ = 20 ± 4 µM), which corresponds to a similar inhibitory activity observed against *P. falciparum* (IC_50_ = 16 ± 2 µM). This finding suggests that brussonol is an inhibitor of different Plasmodium species.

### 2.5. Brussonol Is a Fast-Acting P. falciparum Inhibitor

**Figure 5:**
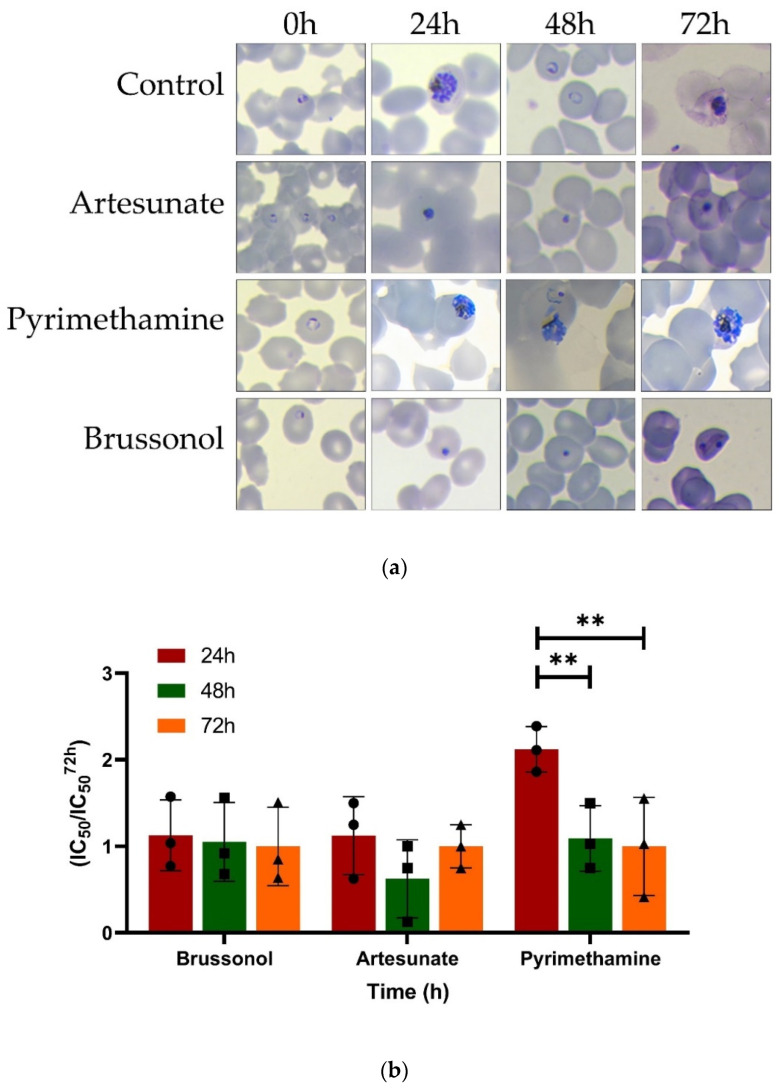
Speed-of-action assessment of brussonol. (**a**) Morphological development evaluation of parasites over time in *P. falciparum* culture stained with giemsa. (**b**) IC_50_ values were determined at 24, 48, and 72 h. Artesunate and pyrimethamine were used as a positive control for fast- and slow-acting inhibition, respectively. The results were normalized with respect to the assessed IC_50_ value at 72 h. Statistical analysis was carried out by using ANOVA (** *p* < 0.01; a *p*-value < 0.05 indicates a significant difference within samples). These data correspond to three independent experiments, mean IC_50_ ± SD.

The speed-of-action assay assesses the inhibitory activity of the tested compounds after 24, 48, and 72 h of drug exposure. The IC_50_ values for each time were defined and then compared to each other to determine whether the compound is a fast- or slow-acting inhibitor. Fast-acting inhibitors show similar IC_50_ values for each assessed time, whereas slow-acting inhibitors show pronounced inhibitory activity over the later hours. Moreover, to confirm the speed-of-action, the morphological development of the parasite in parallel with the IC_50_ assessment was verified. The parasites in the negative control wells developed according to the expected timeline (Figure 5a). Artesunate, a fast-acting inhibitor, caused parasite death within the first 24 h, as verified by the appearance of pyknotic nuclei (Figure 5a). By contrast, pyrimethamine, a slow-acting inhibitor, allowed the parasite to develop until 24 h, delaying the development past this point (Figure 5a). Effective parasite death was observed for pyrimethamine at the 48 and 72 h time points, indicating its slow-acting inhibitory activity. Brussonol caused parasite death within the first 24 h of incubation as observed for artesunate (Figure 5a). At this time point, we verified the appearance of several pyknotic nuclei, suggesting cellular death caused by a toxic effect on the parasite. Furthermore, the IC_50_ values for artesunate and brussonol at 24, 48, and 72 h were in good agreement, whereas the IC_50_ values for pyrimethamine were statistically different between 24 h and 48 h, and 24 h and 72 h (Figure 5b). These findings indicated that brussonol showed inhibitory activity comparable with artesunate, thereby suggesting a fast-acting inhibition.

### 2.6. Brussonol Shows an Additive Combination Profile with Artesunate

**Figure 6:**
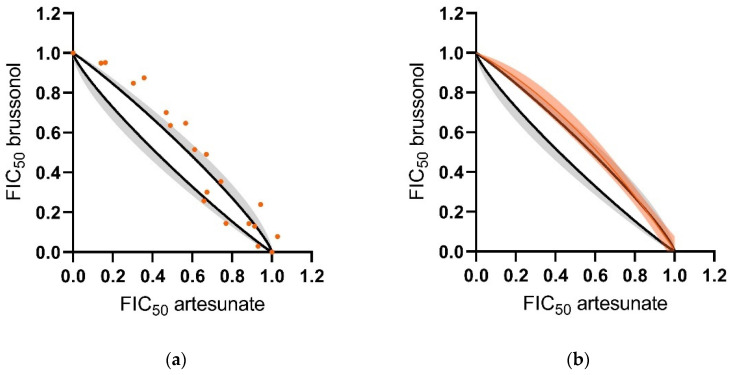
Isobologram and additive isobole were calculated for the combination of brussonol with artesunate. Black lines correspond to the arithmetic average of the upper and lower limits of the additive area, and the gray area indicates the calculated standard deviations. (**a**) Experimentally determined FIC_50_ value pairs are individually represented as orange dots. (**b**) Non-linear fit of experimental data, indicated by the fitted curve (dark orange) and the confidence interval of 95% (clear orange). Data analyzed are from three independent experiments.

A drug combination investigation is important to assess whether the combination of the tested compound with a known antimalarial drug is advantageous or not [25]. In this assay, a fixed molar ratio was used to assess the combination profile of brussonol and artesunate. The range of concentrations tested was based on the assessed IC_50_ values of both the compounds previously determined. In this method, we first determined the additive isobole, which defined the range of IC_50_ values pairs to be attributed to an additive (neutral) character of combination. Next, the combination pairs were plotted. Data points distributed below or above the additive isobole indicate a synergic or antagonistic profile, respectively [26]. The analysis of the isobologram of brussonol in combination with artesunate indicated the experimental combination data do not diverge significantly from the additivity region (Figure 6a,b). Moreover, the sum of fractional inhibitory concentrations in the proportion of 1:1 (respective to each compound’s IC_50_ value) for the brussonol-artesunate pair was 1.1 ± 0.1, while the additive region defined by the additive isobole was 1.0 ± 0.2. Therefore, the absence of a significant difference between these values (*p* = 0.4818) indicated an additive effect in the inhibitory activity when brussonol is used in combination with artesunate. These findings suggest that the combination of brussonol with artesunate was favorable for the in vitro inhibitory activity.

### 2.7. Brussonol Does Not Interfere with Isoprenoid Biosynthesis

**Figure 7:**
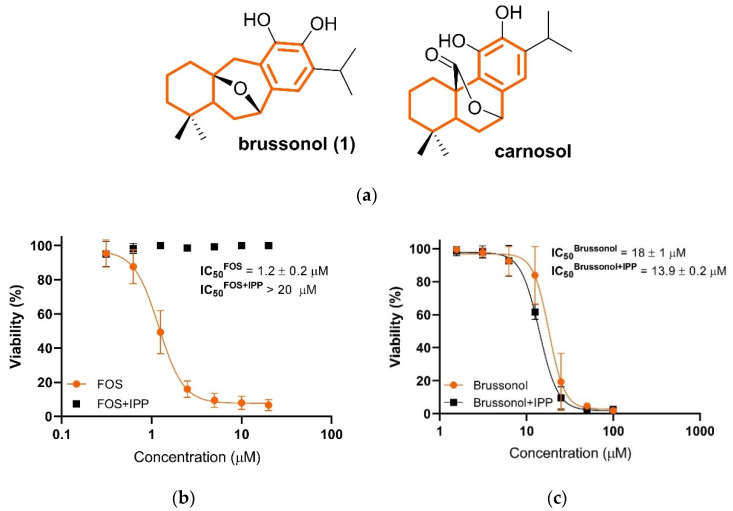
Chemical “rescue” assay to evaluate the action of brussonol (**1**) in the metabolism of isoprenoids (**a**) Molecular structures of carnosol and brussonol [35]. (**b**) Concentration–response curves for fosmidomycin (FOS, positive control) against *P. falciparum* 3D7 strain with and without the supplementation of IPP. (**c**) Concentration–response curves for brussonol against *P. falciparum* 3D7 strain with and without supplementation of IPP (*n* ≥ 2, mean IC_50_ ± SD).

To shed some light on the mode of action underlying brussonol antiplasmodial activity, a structural similarity search in the SciFinder database was performed. The main goal of this approach was to identify whether there were similar compounds with known mechanisms of action. To this end, we set a similarity threshold of >80%. Among the best hits, carnosol (81% similarity), an inhibitor of the squalene synthase (SQS) enzyme, was identified. Carnosol is an essential biomolecule for the biosynthesis of steroids (Figure 7a) [27]. The biosynthesis of steroids is a complex process in which the isoprenoid isopentenyl pyrophosphate (IPP) is one of the precursors [28,29]. In the malaria parasite, the isoprenoid biosynthesis occurs in the apicoplast via an alternate biosynthetic route, the methylerythritol phosphate pathway (MEP), whose components are different from the mevalonate pathway, which is employed by humans to generate isoprenoids [29]. These molecules play central roles in parasite development, including gene expression regulation, and as membrane constituents. Therefore, due to the absence of mammalian homologs and the essentiality of this pathway, the enzymes of the isoprenoid biosynthesis are attractive and validated antimalarial drug targets [30].

To evaluate whether brussonol interfered with the biosynthesis of steroids in malaria parasites, we conducted a chemical rescue assay [31,32,33,34]. The assay identifies compounds that interfere with the metabolism of isoprenoids by “rescuing” their growth inhibition through the supplementation of isopentenyl pyrophosphate (IPP), a key precursor of isoprenoid biosynthesis [31]. Therefore, the assay is useful to identify compounds that affect this metabolic pathway and suggest a putative mode of action for compounds under investigation. Thus, the chemical rescue assay compares the IC_50_ values of the tested compound in the presence and absence of IPP. We used fosmidomycin (FOS) as a positive control for growth inhibition rescuing upon supplementation of IPP (Figure 7b). In this assay, compounds that interfere in the metabolism of isoprenoids show a significant increment in the IC_50_ value in the presence of IPP (Figure 7b). Nevertheless, IPP supplementation did not reverse the growth inhibition by brussonol (Figure 7c). The natural product showed comparable IC_50_ values in the presence (IC_50_ = 13.9 ± 0.2 µM) and absence (IC_50_ = 18 ± 1 µM) of IPP (Figure 7c). These findings indicate that the antiplasmodial activity of brussonol did not rely on the inhibition of the isoprenoid biosynthesis pathway.

### 2.8. Brussonol Induces [Ca^2+^]_cyt_ Rise in P. falciparum

**Figure 8:**
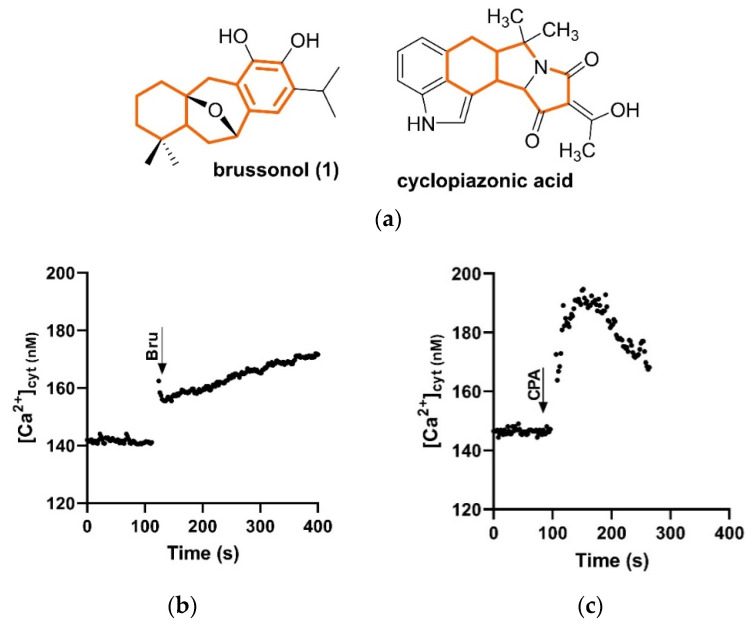

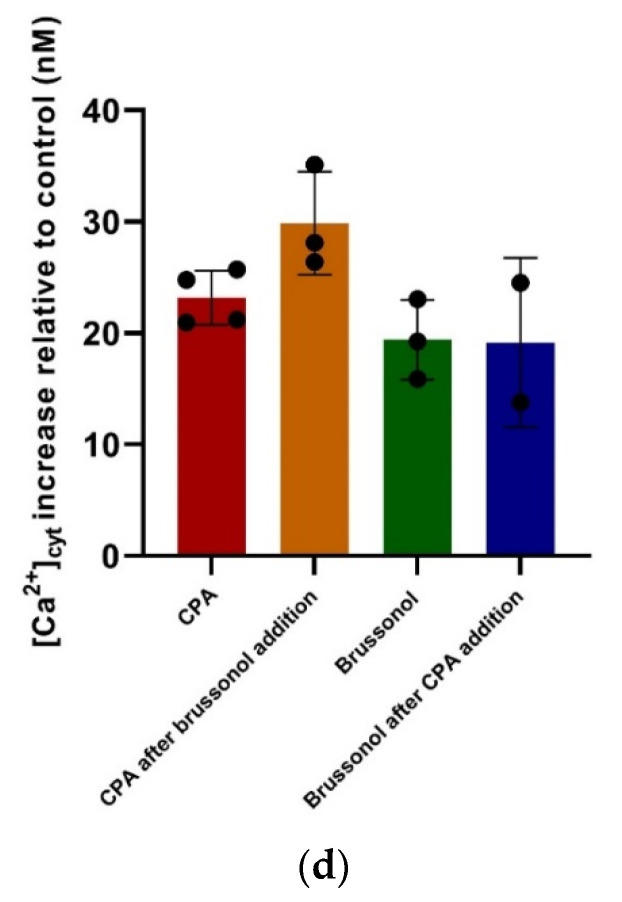
Effect of brussonol on the cytosolic Ca^2+^ mobilization in *P. falciparum* loaded with Fluo-4 AM calcium indicator. (**a**) Chemical structure of brussonol and CPA. The fused rings are highlighted for comparison purposes. Representative traces of [Ca^2+^]_cyt_ rise induced by 10 μM brussonol (**b**) and 10 μM CPA (**c**) in MOPS buffer containing Ca^2+^. (**d**) Histograms of the increase in the cytosolic Ca^2+^ levels with the addition of CPA (10 μM), brussonol (10 μM), and brussonol (10 μM) after and before treatment with CPA (10 μM). The data analyzed were collected from two independent experiments.

One of the compounds identified in the structural similarity search was the cyclopiazonic acid (CPA) (Figure 8a). CPA is a highly selective inhibitor for a *P. falciparum* sarco/endoplasmic reticulum Ca^2+^-ATPase (PfSERCA) pump [36,37,38]. Because of that, brussonol activity on the modulation of the SERCA pump was investigated. To assess the effect of brussonol on calcium mobilization in *P. falciparum* intraerythrocytic stages, isolated parasites loaded with Fluo-4-AM were exposed to 10 μM brussonol in MOPS Ca^2+^ buffer. Brussonol elicited sustained [Ca^2+^]_cyt_ rise (19 ± 4 nM) relative to the basal cytosolic calcium level (Figure 8b,d), which was comparable to the calcium concentration mobilized by CPA (23 ± 2 nM) (Figure 8c,d). To verify if the brussonol-elicited [Ca^2+^]_cyt_ increment originated from the endoplasmic reticulum (ER), targeting the PfSERCA pump, CPA was added before the addition of brussonol. Firstly, as expected, 10 μM CPA significantly led to a transient increment in the cytosolic Ca^2 +^ concentration (23 ± 2 nM) in isolated parasites, consistent with the previous results reported [36,37,38,39]. After the CPA addition reached a plateau, we proceeded with the subsequent addition of 10 μM brussonol, which led to an increase in [Ca^2 +^]_cyt_ (19 ± 8 nM) comparable to brussonol alone (19 ± 4 nM). In the brussonol-pretreated parasites, the addition of CPA still increased the cytosolic Ca^2 +^ level (30 ± 5 nM) (Figure 8d). These results indicated that brussonol induces a [Ca^2+^]_cyt_ rise in *P. falciparum*; however, the calcium mobilization is carried out by a different mechanism than CPA.

## 3. Discussion

The discovery and development of new chemical series to replace the antimalarials with emerging resistance are urgently needed. Brussonol is an attractive natural compound with a new molecular scaffold endowed with promising antiplasmodial activity. The synthetic strategy to obtain brussonol and derivatives required five to seven steps, except for some compounds that required further modification, such as bromination (**10** and **14**, Table 1). In this sense, we designed and assessed the biological activity against *P. falciparum* of 15 brussonol derivatives (Table 1). Of these, seven compounds showed inhibitory activity in the low micromolar range (IC_50_~5 µM), indicating an attractive potential for further potency improvements. For example, compounds **8**, **10**, and **12** showed promising inhibitory potencies against *P. falciparum* (IC_50_s~5 µM), low cytotoxic effects on HepG2 cells (IC_50_s > 170 µM), and reasonable selectivity indexes (SI > 30). Moreover, brussonol (**1**) showed comparable inhibitory potencies against both *P. falciparum* and *P. knowlesi* (IC_50_~20 µM), which indicated that this series has inhibitory activity against different Plasmodium species. The synthesis and assessment of A- and C-ring substitution variants (Figure 1) indicated a clear structure–activity relationship, thereby suggesting that the inhibitory activity of this series can be modulated by suitable chemical groups. For instance, we discovered compounds with 3-fold increased potency compared with brussonol. In this sense, compound **12** stood out as an attractive frontrunner candidate, because it showed inhibitory potency in the low micromolar range (IC_50_ = 5 µM), low cytotoxic effect on liver cells (IC_50_ > 400 µM), and a considerable selectivity index (SI > 62).

A constant challenge for malaria eradication and elimination, as well as for antimalarial drug discovery, relies on the emergence of parasite resistance to available antimalarials [40]. Consequently, it is critical to discover new compounds as drug candidates that overcome the rise of resistance. The Medicines for Malaria Venture (MMV) developed a preclinical resistance assessment strategy to identify active compounds against a collection of resistant strains [41]. In these assays, the IC_50_s of selected compounds are determined against genetically defined resistant strains of *P. falciparum* with naturally occurring known resistance mechanisms. In line with this, we assessed the cross-resistance potential of brussonol and verified that the icetexane diterpenoid scaffold was equipotent against a small panel of resistant *P. falciparum* strains (Figure 4). This finding suggested that brussonol is active against resistant strains of the parasite; in addition, the data indicated that antiplasmodial activity relied on a different mode of action than the gold standard antimalarials.

Another important property of new antimalarials is their speed of action. Ideally, new compounds with antimalarial activity should have a fast onset of action, so that the patient’s symptoms would be rapidly relieved, and a major parasite population in the human host is killed within the first hours of treatment, minimizing the risk of resistance selection [42,43]. Brussonol showed a speed-of-action profile like artesunate, a known fast-acting antimalarial drug. We observed a pronounced inhibitory activity within the first 24 h in the presence of brussonol (Figure 5). Therefore, these parasites did not recover after washing the drug out, indicating that brussonol was effective in killing the parasites.

The in vitro association assay allowed us to determine if a combination of brussonol with artesunate would be advantageous [25]. Several scientific methods to evaluate this in vitro combination are available [44]. In this study, we applied the isobologram analysis combined with fractional inhibitory concentrations (FICs) so that it allowed the identification of what type of interaction (e.g., additive, synergistic, or antagonistic) there is between brussonol and gold-standard antimalarial [45]. The use of ACTs as the first-line treatment for malaria was crucial to slow down the emergence and the spread of artemisinin resistance [41]. In this context, the combined use of multiple agents to treat malaria is recommended, so that the lifespan of antimalarial agents is likely to improve [45]. The analysis of the isobologram obtained in the combination study indicated that brussonol showed an additive profile with artesunate, thereby suggesting that the combination was favorable for the in vitro inhibitory activity (Figure 6). This finding agrees with other reports that showed that hits and lead compounds can be attractive candidates for ACTs and highlights the importance of the assessment of the combination profile in the early phases of the drug discovery pipeline [46,47,48,49].

The identification of the mode of action of new chemical entities (NCE) is a key step in the drug discovery and development paradigm. The definition of how and where a molecule exerts its pharmacological properties significantly helps the lead optimization process of a chemical class and could anticipate the emergence of a resistance mechanism [50]. The study of brussonol’s mode of action started by identifying that the compound did not interfere with hemozoin polymerization (Figure S1). Next, a similarity-based search in SciFinder was performed to identify structural-related compounds to brussonol with any target-associated information. Consequently, this search returned carnosol and cyclopiazonic acid (CPA) as close analogs (Figure 7a and Figure 8a, respectively). Carnosol is a natural product that modulates the biosynthesis of the steroids pathway [27]. Therefore, we tested the ability of brussonol to modulate the isoprenoid biosynthesis pathway. Based on the protocol developed by Yeh and Derisi [31], we verified that brussonol did not interfere in isoprenoid biosynthesis once the parasites treated with this molecule could not be “rescued” by the supplementation with IPP. CPA is a PfSERCA-specific inhibitor that modulates the cytosolic Ca^2+^ homeostasis. In Plasmodium species, Ca^2+^ signaling plays a central role in the parasite life cycle [38,51], including intraerythrocytic parasite proliferation invasion and egress from the host cell, protein secretion, and cell cycle regulation. The endoplasmic reticulum (ER) is the major intracellular Ca^2+^ storage compartment in *P. falciparum* [51] and regulates cytosolic Ca^2+^ through the sarco/endoplasmic reticulum Ca^2+^-ATPase (PfSERCA or PfATP6) pump. The addition of 10 μM brussonol in Fluo-4AM-loaded parasites elicited a sustained [Ca^2+^]_cyt_ rise, which increased steadily even after SERCA was inhibited by CPA. In addition, the calcium mobilization by CPA in the brussonol-pretreated parasites also increased the cytosolic Ca^2+^ levels in the same manner (Figure 8). These results suggested that the increase in [Ca^2+^]_cyt_ by brussonol was not due to the efflux of Ca^2+^ from the ER, thereby indicating that brussonol did not target PfSERCA. These findings are in contrast with that observed with artemisinin derivatives, which act on the ER calcium store [52,53,54,55]. This [Ca^2 +^]_cyt_ rise elicited by brussonol may originate from the extracellular media via Ca^2 +^ influx through membrane calcium channels or other intracellular calcium stores in Plasmodium, including mitochondria or acidic organelles [51]. In sum, although the brussonol target remains unknown, it did interfere with the Ca^2+^ levels within the parasite, which is an interesting result that could explain the additive effect in the inhibitory activity when brussonol was combined with artesunate. In this case, each drug acts in a different parasite calcium source, thereby impacting on calcium homeostasis and parasite development.

## 4. Materials and Methods

### 4.1. Maintenance of In Vitro Culture

The *P. falciparum* strains were cultivated in human blood cells (hRBC) O^+^, while *P. knowlesi* was cultivated in rhesus red blood cells (rRBC) as described [56]. *P. falciparum* complete RPMI (consisting of RPMI-1640, supplemented with 0.2% NaHCO_3_, 25 mM HEPES (pH 7.4), 11 mM D-glucose, 10 mg/L hypoxanthine, 25 mg/L gentamicin, and 0.5% (*m*/*v*) AlbuMAX II) [57]. The parasitemias of these cultures were maintained below 10% with 2.5% hematocrit. *P. knowlesi* complete RPMI (consisting of RPMI-1640 supplemented with 25 mM HEPES, 50 mg/L mM hypoxanthine, 0.26% NaHCO_3_, 10 mg/L gentamicin, and 1% Albumax II) [58]. This culture was maintained with RBCs up to 5% hematocrit. The culture medium was changed daily, and all cultures were maintained under a 90% N_2_/5% CO_2_/5% O_2_ gas mixture at 37 °C. All compounds were purchased from Sigma-Aldrich (Cotia, Brazil).

### 4.2. Biological Activity against P. falciparum Blood-Stage Parasites In Vitro

The antiplasmodial activity of brussonol and derivatives was evaluated against *P. falciparum* blood parasites 3D7(chloroquine-sensitive). The parasites were synchronized through sterile 5% (*m*/*v*) D-sorbitol treatment over 10 min at 37 °C for the enrichment of ring stages [59]. Centrifugation 600× *g* over 5 min was used to pellet the cultures. The parasitemia was determined by microscope analysis of thin blood smears stained with Giemsa 10% (*v*/*v*) after fixation with methanol. The initial parasitemia was calculated for 1000 red blood cells (RBCs), and cultures were diluted to 0.5% parasitemia and 2% hematocrit by the addition of the appropriate volumes of blood and medium. A total of 180 µL aliquots of parasites were distributed into 96-well plates followed by the addition of 20 µL of previously prepared aliquots of ten-fold concentrated compounds, the range of concentrations tested was 10–1.53 µM. Negative and positive control wells, which correspond to non-parasitized erythrocytes and parasite cultures in the absence of compounds, were set in parallel. The DMSO concentration was maintained below 0.05% (*v*/*v*) for all compounds except for brussonol, which was 0.5%. The plates were incubated for 72 h at 37 °C in a humidified incubator with a gas mixture of 90% N_2_, 5% O_2,_ and 5% CO_2_. Each test was performed in duplicates, and the results were compared with the control cultures. Once completed incubation, the culture medium was removed, and the cells were resuspended in 100 µL PBS (116 mM NaCl, 10 mM NaH_2_PO_4_, 3 mM KH_2_PO_4_) and lysed with 100 µL lysis buffer (20 mM Tris base, 5 mM EDTA, 0.0008% (*v*/*v*) Triton X-100, 0.008% (*m*/*v*) saponin, pH 8.0) containing 0.002% (*v*/*v*) SYBR Green I. Plates were incubated for 30 min at room temperature, and a SpectraMAX Gemini EM plate reader (Molecular Devices Corp., Sunnyvale, CA) was used to determine the fluorescence corresponding to parasitic density (excitation at 485 nm, emission at 535 nm) [60]. The half-maximal inhibitory concentration (IC_50_^Pf^) was determined by non-linear regression analysis of the resulting concentration–response curve using the software GraphPad Prism version 8.0.1 for Windows, GraphPad Software, San Diego, California USA, www.graphpad.com, accessed on 3 March 2022”.

### 4.3. Cytotoxic Tests Using Immortalized Cells

The cytotoxic effects of brussonol and derivatives were evaluated against the human hepatocellular carcinoma cell line (HepG2). The cells were cultivated in an RPMI medium supplemented with 10% (*v*/*v*) fetal bovine serum and 25 µg/mL gentamicin. Conditions for the cultivation of these cells were 37 °C and 5% CO_2_, and every 2 days the supplemented medium was changed.

For experimental procedures, the cells were trypsinized and transferred to a 96-well plate at 30,000 cells per well (180 µL). The plate was incubated at 37 °C and 5% CO_2_ to allow cell adhesion. Next, 20 µL of serial dilutions of the compounds tested were added to the plate, with a range of concentrations tested from 400–6.25 µM. Cells without any compounds were used as a positive control for growing and the wells containing only medium were used as negative controls. The plate was incubated for 72 h at 37 °C and 5% CO_2_. After incubation, a microscope was used to determine the highest compound concentration to be used in the experiments (highest concentration without precipitation). A colorimetric assay was used to evaluate cytotoxicity. This assay is based on the metabolic cell activity in the presence of 3-(4,5-dimethylthiazol-2-yl)-2,5-diphenyltetrazolium bromide (MTT) [61]. Shortly after incubation, 20 µL of a solution of MTT at 5 mg/mL (solubilized in phosphate buffer) is added to each well. The plate is incubated for 3 to 4 h at 37 °C to allow MTT cleavage in living cells. Then, the supernatant is removed, and 100 µL of dimethylsulfoxide (DMSO) is added to solubilize the purple formazan crystals. The absorbance, which is proportional to the number of viable cells, was determined using a SpectraMAX Plus 384 plate reader (Molecular Devices Corp., Sunnyvale, CA) (λ = 570 nm). All compounds described here were purchased from Sigma-Aldrich (verificar local).

### 4.4. Calculation of Selectivity Index (SI)

After the IC_50_ against the parasite and the human cell line is determined, it is possible to calculate the SI. It is calculated as the formula below:

SI = IC_50_^HepG2^/IC_50_*^Pf^*.

The SI shows the difference between the inhibitory potency against the parasite and the cytotoxic concentration for mammalian cells. For our reference, compounds with SI values higher than 10 are considered well tolerated by the cellular model used, and they are considered for further evaluation.

### 4.5. In Vitro Evaluation against P. knowlesi

Blood-stage *P. knowlesi* cultures were diluted to 0.5% parasitemia and brought to 2% hematocrit for growth in 96-well plates containing the desired drug concentration series (200 µL of culture/well). Plates were incubated for 40 h at 37 °C in a humidified chamber containing a gas mixture of 5% CO_2_, 5% O_2_, and 90% N_2_. Parasite growth in the plates was measured by luminescence (NanoLuc method) [62]. In this method, plates were removed from the incubator and 100 µL of NanoGlo IC50 solution (Promega NanoGlo reaction mixture diluted tenfold in PBS) were added to each well and mixed by pipetting. The plates were then incubated for 3 min at room temperature and the luminescence was assessed using a plate luminometer (Molecular Devices, San Jose, CA, USA) with 1 s integration time. Luminescence readings were normalized to values from control wells containing no drug. IC_50_ values were determined from fitted response curves (non-linear regression with variable slope, GraphPad Prism Software), and data from at least two independent assays were used to calculate the average IC_50_ value of the *P. knowlesi* pvcen-pvhsp70-DNanoLuc transgenic line with each method.

### 4.6. Resistance Assessment

The antiplasmodial activity of brussonol was assessed against a panel of *P. falciparum* strains: 3D7 (chloroquine-sensitive), K1 (resistant to chloroquine, mefloquine, and sulfadoxine), Dd2 (resistant to chloroquine, mefloquine, and pyrimethamine), TM90C6B (resistant to atovaquone), and 3D7^r^-MMV848 (resistant to MMV692848, a PI4K inhibitor). The assay to determine the IC_50_ value of brussonol against the panel of resistant strains was carried out as described above. After the determination of the IC_50_ value for each resistant strain, a resistance index (RI) was calculated using the following equation:

RI = IC_50_^Resistant strain^/IC_50_^3D7^

RI values greater than 3 were considered indicative of cross-resistance.

### 4.7. Speed-of-Action Assay

To determine whether brussonol is a fast- or slow-acting inhibitor, a protocol adapted from Terkuile and collaborators (1993) [63] was used. In this method, three 96-well plates were prepared by adding 180 µL of the parasite’s inoculum with 0.5% parasitemia and 2% hematocrit. A serial dilution of the compounds was added to each plate; the starting concentration for brussonol was 6× IC_50_, while for artesunate and pyrimethamine (fast- and slow-acting controls, respectively) were 10× IC_50_. In addition, a positive and negative control, which corresponded to parasite cultures with no addition of inhibitor and non-parasitized erythrocytes, respectively, were used for the parasite’s control growth. Each plate was treated for a different period (24, 48, or 72 h), and the first two plates were washed twice with fresh medium to remove the inhibitor, followed by incubation of 48 and 24 h, respectively. After 72 h of incubation at 37 °C, all plates were evaluated using SYBR Green I assay to determine the IC_50_ for each treatment. A statistical analysis using a one-way ANOVA test was done to compare each time point using GraphPad Prism version 8.0.1 for Windows, GraphPad Software, San Diego, California USA, www.graphpad.com, accessed on 1 February 2022. In parallel, the morphological development of the parasite under the compounds’ pressure was assessed by adding the compounds tested at the highest concentration evaluated. Blood smears of each well were made and stained at time points 0, 24, 48, and 72 h.

### 4.8. In Vitro Combination with Artesunate

This assay was adapted from the work done by Fivelman and collaborators (2004) [64]. Consideration of additivity ranges was included in the analysis, as described by Grabovsky and Tallarida (2004) [26]. Brussonol and artesunate were diluted and combined in a 96-well plate in seven fixed-ratio combinations (1:0, 6:1, 5:2, 4:3, 3:4, 2:5, 1:6, 0:1). Starting concentrations were 6× IC_50_ for both compounds. This experiment was performed with 0.5% parasitemia and 2% hematocrit, and controls were drug-free non-parasitized erythrocytes and parasitized erythrocytes. Serial dilutions of these combinations were prepared and incubated with the parasite as described above to determine the antiplasmodial activity against *P. falciparum*. The SYBR Green I test was applied to determine the IC_50_ value for each combination, using the software GraphPad Prism version 8.0.1 for Windows, GraphPad Software, San Diego, CA, USA, www.graphpad.com, accessed on 12 August 2020. The absolute IC_50_s of brussonol and artesunate (proportions 1:0 and 0:1), the partial IC_50_s (IC_50_^brussonol^ and IC_50_^artesunate^) for each combination (6:1, 5:2, 4:3, 3:4, 2:5, 1:6), and the fractional inhibitory concentrations for each combination were determined. FIC pairs were plotted as points in isobolograms, and if the majority of the FIC_50_ pairs were above or below the additive range, the combination evaluated showed antagonism or synergic interaction, respectively.

### 4.9. Chemical Rescue Assay

The chemical rescue assay was carried out as described by Yeh and DeRisi (2011). This assay consists in verifying the IC_50_ value of the tested compounds with and without the isopentenyl pyrophosphate (IPP), purchased from Isoprenoids, LC (Florida, USA). Briefly, 3D7 strain ring stages were cultured in 96-well plates, with 0.5% parasitemia and 2% hematocrit. Non-parasitized erythrocytes and parasitized erythrocytes without drugs were used as growth controls, and fosmidomycin (FOS) was used as a positive control for chemical rescue. Serial dilutions of brussonol and FOS were prepared in duplicates. In the same 96-well plate, brussonol and FOS had their antiplasmodial activity evaluated with and without supplementation of 200 µM IPP. Plates were incubated for 72 h and analyzed by the SYBR Green I assay.

### 4.10. [Ca^2+^]_cyt_ Measurements

Isolated parasites were incubated for 1 h at 37 °C with 5 μM of Fluo-4AM in 1 mL MOPS buffer (116 mM NaCl, 5.4 mM KCl, 0.8 mM MgSO4, 5.5 mM D-glucose, 50 mM MOPS, and 2 mM CaCl2, pH 7.2), with 1 mM probenecid to minimize compartmentalization and extrusion of probe. The parasites were washed three times with the same buffer and transferred to a quartz cuvette. Intracellular calcium was measured continuously using a Hitachi F-7000 spectrofluorimeter (Tokyo, Japan) by measurement of the fluorescence (λex = 488 nm and λem = 530 nm) at 37 °C, under agitation. When indicated, 10 µM of cyclopiazonic acid (CPA) or 10 µM of brussonol were added to the cuvette. Maximal fluorescence (F_max_) was determined after the lysis of with digitonin 0.15% (*m*/*v*), and minimal fluorescence (F_min_) was determined by adding 25 mM EGTA in 3 M Tris, pH 8.8, until no further decrease in fluorescence was observed. The cytosolic calcium concentration ([Ca^2+^]_cyt_) was calculated from the fluorescence data (F) using a K_d_ value of 345 nM using the formula:

Ca^2 +^_cyt_ = 345 × [(F − F_min_)/(F_max_ − F)]

The results (*n* = 2) were analyzed through One-Way ANOVA with Tukey post-test. All compounds described here were purchased from Sigma-Aldrich (verificar local).

## 5. Conclusions

Brussonol and analogs belong to a chemical class of natural product derivatives, which showed promising antiplasmodial profile, including low propensity to cross-resistance, fast-acting inhibition, and an additive profile in combination with artesunate. Mode of action investigation indicates a brussonol-induced [Ca^2+^]_cyt_ rise in *P. falciparum*, suggesting that the compound modulates the calcium homeostasis and parasite development. To the best of our knowledge, this is the first report on the antimalarial activity of the icetexane diterpenoid scaffold. Our findings indicated that the brussonol and analogs are new molecular scaffolds endowed with attractive antiplasmodial activities that justify the design of new derivatives with improved properties to deliver new lead candidates for malaria.

## Figures and Tables

**Table 1:** In vitro antiplasmodial activity, cytotoxicity, and SI of brussonol and its derivatives.

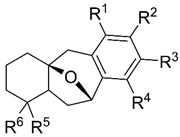									
**Code**	**R^1^**	**R^2^**	**R^3^**	**R^4^**	**R^5^**	**R^6^**	**IC_50_*^Pf^* (µM)**	**IC_50_^HepG2^ (µM)**	**SI ^1^**
**1**	OH	OH	*i*Pr	H	Me	Me	16 ± 2	67 ± 4	4
**2**	OMe	OMe	H	H	H	H	>10	ND ^2^	ND ^2^
**3**	OMe	H	H	OMe	H	H	>10	ND ^2^	ND ^2^
**4**	OH	OH	H	H	H	H	>10	ND ^2^	ND ^2^
**5**	OH	H	H	OH	H	H	>10	ND ^2^	ND ^2^
**6**	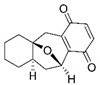						>10	ND ^2^	ND ^2^
**7**	OMe	OMe	*i*Pr	H	Me	Me	5.2 ± 0.4	>192	>37
**8**	OMe	OMe	*i*Pr	OMe	Me	Me	5 ± 2	170 ± 38	34
**9**	OMe	OMe	H	H	Me	Me	>10	ND ^2^	ND ^2^
**10**	OMe	OMe	*i*Pr	Br	Me	Me	5.6 ± 0.1	>400	>70
**11**	OMe	OMe	*i*Pr	Ph	Me	Me	5.8 ± 0.2	>12	>2
**12**	OMe	OMe	*i*Pr	H	H	H	5.6 ± 0.8	>400	>62
**13**	OH	OH	*i*Pr	H	H	H	>10	ND ^2^	ND ^2^
**14**	OMe	OMe	*i*Pr	Br	H	H	5.65 ± 0.05	>25	>4
**15**	OMe	OMe	*i*Pr	Ph	H	H	5.89 ± 0.02	>50	>8

^1^ SI = IC_50_^HepG2^/IC_50_*^Pf^*, ^2^ ND = not determined.

**Table 2:** IC_50_ values of compound brussonol against a panel of sensitive and resistant *P. falciparum* strains.

*Pf* Strain	IC_50_^brussonol^ (µM)	RI	IC_50_^PYR^ (µM)	RI	IC_50_^ATV^ (µM)	RI	IC_50_^ART^ (µM)	RI	IC_50_^MMV848^ (µM)	RI
3D7	13 ± 2	1	0.06 ± 0.01	1	0.0007 ± 0.0003	1	0.008 ± 0.004	1	0.13 ± 0.02	1
K1	6.6 ± 0.1	0.5	>10	>166	NT	NT	0.007 ± 0.003	1	NT	NT
Dd2	7 ± 2	0.5	>10	>166	NT	NT	0.011 ± 0.008	1	NT	NT
TM90C6B	7 ± 2	0.5	NT	NT	3 ± 1	4285	0.006 ± 0.002	1	NT	NT
3D7^r^_MMV848	12 ± 3	1	NT	NT	NT	NT	0.013 ± 0.008	2	2.4 ± 0.4	18

Data represent mean ± standard deviation of at least two independent experiments conducted in triplicate; RI = IC_50_^resistant strain^/IC_50_^3D7^; NT = not tested; PYR = pyrimethamine; ATV = atovaquone; ART = artesunate; MMV848 = *Pf*PI4K inhibitor.

## Data Availability

Data are contained within the article and supplementary materials.

## Supplementary Materials

The following supporting information can be downloaded at: https://www.mdpi.com/article/10.3390/ph15070814/s1, Experimental details on the synthesis of compounds **1**–**15**. Figure S1: Hemozoin formation inhibition assay. NMR spectra of compounds **1**–**15**. References [65,66,67,68,69] are cited in the supplementary materials.
