# Supplementary material for: Synthesis, Structure–Activity Relationships, and Parasitological Profiling of Brussonol Derivatives as New Plasmodium falciparum Inhibitors
Source: Pharmaceuticals (Basel). 2022 Jun 30;15(7):814. doi: 10.3390/ph15070814 (PMC9321043; doi:10.3390/ph15070814)
Supplement: Supplementary file 1 [file pharmaceuticals-15-00814-s001.zip › pharmaceuticals-1777140-supplementary.pdf]

SUPPLEMENTARY MATERIAL

Synthesis, structure-activity relationships, and parasitological profiling of brussonol derivatives as new *Plasmodium falciparum* inhibitors

Camila S. Barbosa[1], Anees Ahmad[2], Sarah E. C. Maluf[1], Igor M. R. Moura[1], Guilherme E. Souza[1], Giovanna A. H. Guerra[3], Roberto R. M. Barros[3], Marcos L. Gazarini[4], Anna C. C. Aguiar[1,4*], Antônio C. Burtoloso[2*], Rafael V. C. Guido[1,*]

[1] São Carlos Institute of Physics, University of São Paulo, Av. João Dagnone, 1100, Santa Angelina, São Carlos, SP, 13563-120, Brazil

[2] São Carlos Institute of Chemistry, University of São Paulo, Av. João Dagnone, 1100, Santa Angelina, São Carlos, SP, 13563-120, Brazil

[3] Department of Microbiology, Immunology and Parasitology, Escola Paulista de Medicina, Federal University of São Paulo, São Paulo, Brazil

[4] Department of Biosciences, Federal University of São Paulo, Santos, SP, Brazil

Table of Contents

1. Experimental Section: Synthesis..02
2. Hemozoin Formation Polymerization Inhibition..23
3. References..24
4. NMR Spectra..25

1. Experimental Section: Synthesis

All commercially available reagents were used without further purification unless otherwise noted. All solvents used for reactions and chromatography were dried and purified by standard methods[1]. TLC analyses were performed using silica gel 60F 254 precoated plates, with detection by UV-absorption (254 nm) and by spraying with *p*-anisaldehyde, potassium permanganate and phosphomolybdic acid solutions followed by charring at ~150 °C for visualization. Flash column chromatography was performed using silica gel 200-400 Mesh. All NMR analyses were recorded on Agilent NMR Spectrometer (400 MHz or 500 MHz) using $CDCl_3$ as solvent and TMS as internal standard. Chemical shifts are reported in ppm downfield from TMS with reference to internal solvent. Infrared spectra were obtained using FT-IR (Bruker, model ALPHA) at 4.0 cm^{-1} resolution and are reported in wavenumbers. The samples were dispersed using DCM as solvent. High-resolution mass spectra (HRMS) were recorded using electron spray ionization in positive mode (ESI) in a Waters, model Xevo G2 or in a ThermoFischer, model Orbitrap LTQ Velos. For all the known compounds ^{1}HNMR data matched with that of the previously reported.

2-allylcyclohexan-1-one (32)[2]

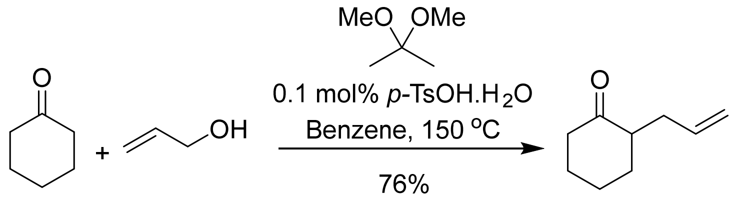

31 **29**

In a 100 ml round bottom flask equipped with a short-path distillation flask, the mixture of cyclohexanone (**31**) (3.00 g, 3.17 mL, 30.6 mmol), allyl alcohol (3.91 g, 4.57 mL, 67.3 mmol), 2,2-dimethoxypropane (6.37 g, 7.52 mL, 61.2 mmol), 4-methylbenzenesulfonic acid hydrate (0.006 g, 0.03 mmol), and benzene (50 mL) was heated at 90 °C until the acetone and the benzene-methanol azeotrope are completely removed. After the distillation had completed, the mixture was heated at 160 °C for 30 min. Vacuum distillation (45 °C, 1 torr) gives 2-allylcyclohexanone (**2**) in 76% (3.22 g, 23, 3 mmol) yield as a clear oil. ^{1}H NMR (500 MHz, $CDCl_3$) δ: 5.81-5.73 (m, 1H), 5.05-4.98 (m, 2H), 2.57-2.51 (m, 1H), 2.42-2.27 (m, 3H), 2.16-2.03 (m, 2H), 1.98 (dt, *J*= 14.5, 7.5 Hz, 1H), 1.88-1.84 (m, 1H), 1.71-1.61 (m, 2H), 1.40-1.32 (m, 1H).

4-allyl-1-oxaspiro[2.5]octane (16)[2]

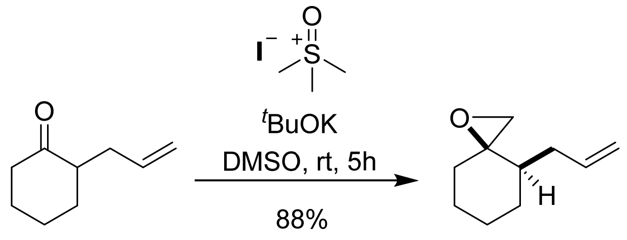

Trimethylsulfoxonium iodide (2.58 g, 11.7 mmol) was added to dry DMSO (25 mL) and stirred until complete dissolution of the salt. Addition of allyl cyclohexanone **29** (1.00 g, 7.3 mmol), followed by addition of solution of potassium tert-butoxide (1.3 g, 11.6 mmol) in DMSO (17 mL). The resulting solution was kept under stirring at room temperature for 5 hours. After this time, H_2O (50 mL) was added, the aqueous phase was extracted with ethyl ether (3 x 20 ml), washed with H_2O (17 ml), dried over anhydrous $MgSO_4$ and the solvent was evaporated. Column chromatography: 2-4% ethyl acetate/hexanes. Yield: 88% (0.98 g, 6.5 mmol). Colorless oil. ^{1}H NMR (500 MHz, $CDCl_3$) δ: 5.79-5.70 (m, 1H); 5.04-4.97 (m, 2H); 2.75 (dd, *J*= 5.0, 0.8 Hz, 1H); 2.52 (d, *J*= 4.5 Hz, 1H); 2.22-2.16 (m, 2H); 2.04-1.97 (m, 1H); 1.76-1.70(m, 1H), 1.67-1.51 (m, 6 H), 1.47-1.39 (m, 2H).

(((1S,2R)-2-allyl-1-(2,3-dimethoxybenzyl)cyclohexyl)oxy)trimethylsilane (32)[2]

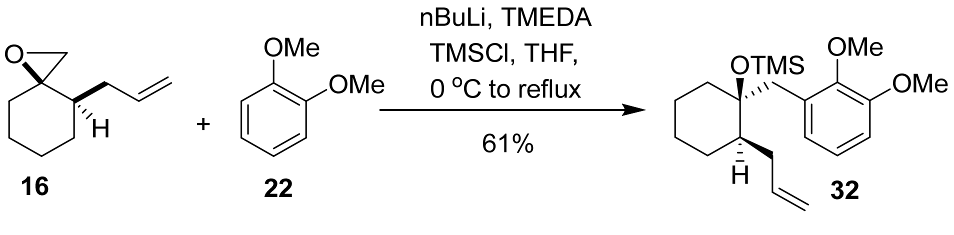

To a solution of 1,2-dimethoxybenzene (**22**) (0.82 g, 5.9 mmol) in dry THF (19.7 mL) was added 2.15M nBuLi (3.4 mL, 5.9 mmol) dropwise under stirring at ºC. It was allowed to stirr at room temperature for 2.5 hours. After addition of TMEDA (0.68 g, 0.89 mL, 5.9 mmol) solution was warmed to reflux. A solution of epoxide **16** (0.30g, 1.97 mmol) in THF (1.97 mL) was added under N_2 at -78 ºC and was kept under stirring for 3 hours at rt. After cooling the reaction mixture to 0 ºC, TMSCl (1.25 mL, 9.8 mmol) was added and allow to stir for overnight. After addition of aqueous sodium bicarbonate solution (15 mL) it was extracted with DCM (3 x 10 mL). The combined organic phase was dried over anhydrous $MgSO_4$ and the solvent was evaporated. Column chromatography: 2-4% ethyl acetate/hexanes. Yield: 61% (0.44 g, 1.12 mmol). yellowish oil. ^{1}H NMR (500 MHz, $CDCl_3$) δ: 6.96 (t, *J*= 7.8 Hz, 1H), 6.79 (d, *J*= 8.5 Hz, 2H), 5.88-5.80 (m, 1H), 5.06-4.98 (m, 2H), 3.87 (s, 3H). 3.80 (s, 3H), 3.06 (d, *J*= 13.0 Hz, 1H), 2.93 (d, *J*= 13 Hz, 1H), 2.68-2.65 (m, 1H), 1.95-1.88 (m, 1H), 1.77-1.74 (m, 1H), 1.55-1.16 (m, 8H), 0.17 (s, 9H).

2-((1R,2S)-2-(2,3-dimethoxybenzyl)-2- ((trimethylsilyl)oxy)cyclohexyl)acetaldehyde (33)[2]

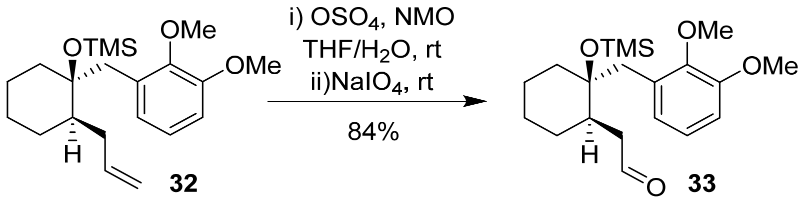

To a solution of compound **32** (0.85 g, 2.3 mmol) and NMO (0.57 g, 4.8 mmol) in THF/H_2O (3:1, 40 mL) under stirring at room temperature was added of 0.15 M OsO_4 (1.5 mL, 0.22 mmol) and allowed to stir for overnight. After addition of $NaIO_4$ (1.03 g, 4.8 mmol) the mixture stirred at room temperature for further 2 hours. To this reaction mixture 10% $Na_2S_2O_3$ (10 mL) solution was added and extracted with diethyl ether (3 x 10). The combined organic phase was washed with H_2O (10 mL) and saturated NaCl solution (10 mL), dried over anhydrous $MgSO_4$ and the solvent was evaporated. Column chromatography: 5-8% ethyl acetate/hexanes. Yield: 84% (0.69 g, 1.90 mmol). Yellowish oil. ^{1}H NMR (500 MHz, $CDCl_3$) δ: 9.76 (dd, *J*= 4.0, 1.5 Hz, 1H), 6.96 (dd, *J*= 8.0, 1.5 Hz, 1H), 6.77 (d, *J*= 8.0, 1.5 Hz, 1H), 3.86 (s, 3H). 3.79 (s, 3H), 3.05 (d, *J*= 13.0 Hz, 1H), 2.89 (d, *J*= 13 Hz, 1H), 2.76 (ddd, *J*= 16, 3.5, 1.5, 1H), 2.40 (ddd, *J*= 16, 8.5, 3.5 Hz, 1H), 1.90-1.86 (m, 1H), 1.77 (dt, *J*= 13.0, 4.0 Hz, 1H), 1.55-125 (m, 7H), 0.17 (s, 9H).

(4aS,10S,11aR)-6,7-dimethoxy-1,2,3,4,5,10,11,11a-octahydro-4a,10-epoxydibenzo[a,d][7]annulene (2)[2]

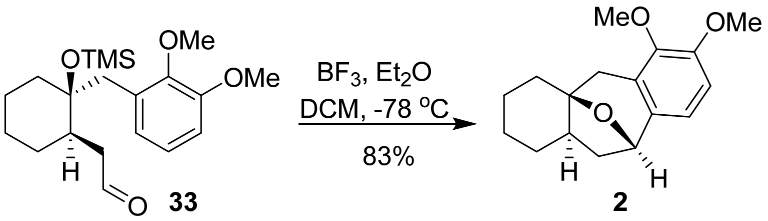

To a solution of aldehyde **33** (0.375 g, 1.03 mmol) dissolved in DCM (11 mL) was added BF3•OEt2 (0.25 mL, 2.06 mmol) dropwise under N_2 at -78 ºC until complete consumption of starting material was observed by TLC analysis (2 h). The reaction was then quenched with saturated aqueous ammonium chloride. The aqueous layer was then extracted (3 × 5 mL) with EtOAc. The combined organic extracts were dried with anhydrous $MgSO_4$, filtered, and concentrated under reduced pressure. Column chromatography: 5% ethyl acetate/hexanes. Yield: 83% (0.23 g, 0.84 mmol). White solid. ^{1}H NMR (500 MHz, $CDCl_3$) δ: 6.69 (d, *J*= 8.5 Hz, 1H), 6.68 (d, *J*= 8.5 Hz, 1H), 4.94 (d, *J*= 6.4 Hz, 1H), 3.83 (s, 3H). 3.80 (s, 3H), 2.82 (d, *J*= 17.5 Hz, 1H), 2.63 (d, *J*= 17.5 Hz, 1H), 2.05-1.87 (m, 4H), 1.73-1.58 (m, 5H), 1.29-1.21 (m, 2H).

(4aS,10S,11aR)-1,2,3,4,5,10,11,11a-octahydro-4a,10-epoxydibenzo[a,d][7]annulene-6,7-diol (4)

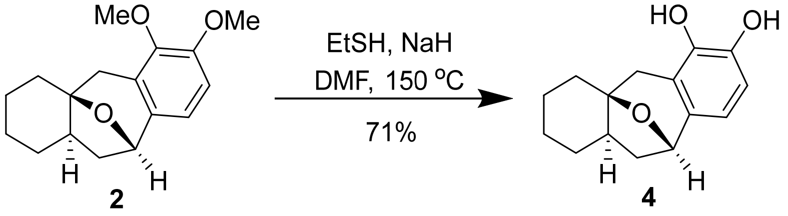

Under inert atmosphere N_2, NaH (0.212 g, 5.3 mmol, 60% in mineral oil) was washed with anhydrous hexanes (3 x 20 mL). After a few minutes, anhydrous DMF (15 mL) was added. To this mixture was slowly added a solution of EtSH (0.6 mL, 7.92 mmol) in anhydrous DMF (0.6 mL) at 0 °C and the resulting yellow solution was stirred for 20 min at rt. A solution of compound **2** (0.12 g, 0.44 mmol) in DMF (2 mL) was then added dropwise and the resulting mixture was stirred for 5 h at 150 °C. When the reaction became slightly brown, the mixture was cooled to the rt and a saturated solution of NH_4Cl (10 mL) was added. The mixture was extracted with Et_2O (3 x 10 mL). The organic phase was washed with H_2O, saturated NaCl solution and dried over anhydrous $MgSO_4$. The solvent was removed under reduced pressure. Column chromatography: 20-40% ethyl acetate/hexanes. Compound **4** yield: 81% (0.87 g, 0.35 mmol). White solid. ^1H NMR (500 MHz, $CDCl_3$) δ: 6.59 (d, *J*= 8.0 Hz, 1H), 6.44 (d, *J*= 8.0 Hz, 1H), 4.92 (d, *J*= 7.0 Hz, 1H), 2.77 (d, *J*= 17.0 Hz, 1H), 2.53 (d, *J*= 17.0 Hz, 1H), 2.05-2.01 (m, 2H), 1.98-1.95 (m, 1H), 1.92-1.87 (m, 1H), 1.73-1.54 (m, 7H). ^{13}C NMR (125 MHz, $CDCl_3$) d: 142.2, 141.5, 136.3, 119.4, 114.8, 112.3, 79.5, 75.9, 44.1, 42.2, 37.6, 33.4, 30.6, 21.7, 19.7. HRMS (ESI/TOF) m/z: $[M + H]^+$ Calculated for $C_{15}H_{19}O_3$ 247.1329; Found 247.1344.

(1S,2R)-2-allyl-1-(2,5-dimethoxybenzyl)cyclohexan-1-ol (34)

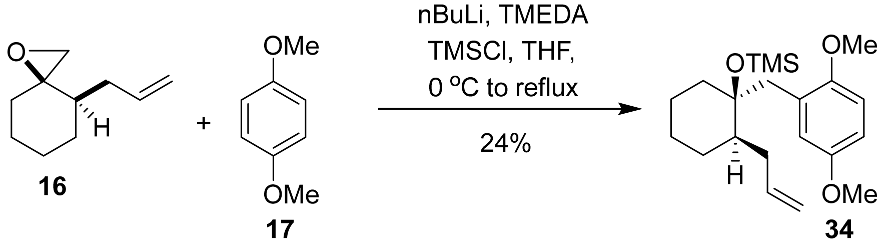

To a solution of 1,4-dimethoxybenzene (**17**) (0.41 g, 3.0 mmol) in dry THF (11 mL) was added 1.7M nBuLi (1.7 mL, 3.0 mmol) dropwise under stirring at ^0C. It was allowed to stir at room temperature for 2.5 hours. After addition of TMEDA (0.35 g, 0.45 mL, 5.9 mmol) solution was warmed to reflux. A solution of epoxide **16** (0.15 g, 1.0 mmol) in THF (1 mL) was added under N_2 at -78 ^0C and kept under stirring for 4 hours at rt. After addition of aqueous NH_4Cl solution (15 mL) it was extracted with DCM (3 x 10 mL). The combined organic phase was dried over anhydrous $MgSO_4$ and the solvent was evaporated. Column chromatography: 5-10% ethyl acetate/hexanes. Yield: 24% (0.07 g, 0.24 mmol). Colorless viscous liquid. ^1H NMR (500 MHz, $CDCl_3$) δ: 6.81 (d, *J*= 9.0 Hz, 1H), 6.73 (dd,

J= 8.5, 3.0 Hz, 1H), 6.70 (d, J= 3.5 Hz, 1H), 5.89-5.81 (m, 1H), 5.08-5.0 (m, 2H), 3.80 (s, 3H), 3.76 (s, 3H), 3.32 (d, J= 13.5 Hz, 1H), 2.87 (s, 1H), 2.62- 2.57 (m, 1H), 2.50 (d, J= 13.5 Hz, 1H), 2.07-2.01 (m, 1H), 1.65-1.62 (m, 2H), 1.46-1.34 (m, 5H), 1.25-1.16 (m, 2H). ^{13}C NMR (125 MHz, $CDCl_3$) δ: 153.6, 151.8. 138.8, 127.9, 119.1, 115.5, 111.8, 111.5, 73.7, 56.1, 55.8, 45.7, 41.7, 36.9, 34.2, 27.5, 25.5, 22.0. νmax (cm^{-1}): 3030, 2951, 2851, 1649, 1521, 1479, 1256, 1073. HRMS (ESI/TOF) m/z: [M + Na]$^+$ Calculated for $C_{18}H_{26}NaO_3$ 313.1774; Found 313.1749.

2-((1R,2S)-2-(2,5-dimethoxybenzyl)-2-((trimethylsilyl)oxy)cyclohexyl)acetaldehyde (18)

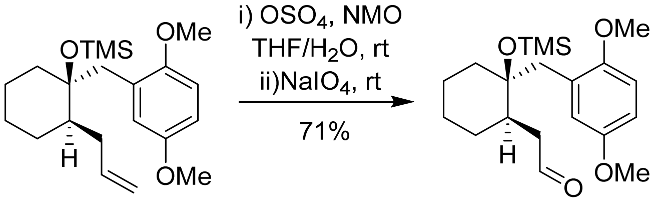

To a solution of alkene **34** (0.23 g, 1.3 mmol) and NMO (0.31 g, 2.6 mmol) in THF/H_2O (3:1, 20 mL) under stirring at room temperature was added of 0.15 M OsO_4 (0.17 mL, 2.0 mol%) and allowed to stir for overnight. After addition of $NaIO_4$ (0.58 g, 2.7 mmol) the mixture stirred at room temperature for further 2 hours. To this reaction mixture 10% $Na_2S_2O_3$ (10 mL) solution was added and extracted with diethyl ether (3 x 10). The combined organic phase was washed with H_2O (10 mL) and saturated NaCl solution (10 mL), dried over anhydrous $MgSO_4$ and the solvent was evaporated. Column chromatography: 5-8% ethyl acetate/hexanes. Yield: 71% (0.06 g, 0.16 mmol). Colorless viscous liquid. ^{1}H NMR (500 MHz, $CDCl_3$) δ: 9.77 (dd, J= 3.5 1.5 Hz, 1H), 6.78-6.73 (m 2H), 3.76 (s, 3H). 3.72 (s, 3H), 3.06 (d, J= 13.0 Hz, 1H), 2.86 (ddd, J= 16.5, 3.5, 1.5 1H), 2.80 (d, J= 13 Hz, 1H), 2.38 (ddd, J= 17, 8.5, 3.5 Hz 1H), 1.86-1.82 (m, 1H), 1.77-173 (m, 1H), 1.54-149 (m, 4H), 1.46-1.39 (m, 3H), 0.16 (s, 9H). ^{13}C NMR (125 MHz, $CDCl_3$) δ: 204.2, 153.0, 152.2, 127.2, 118.7, 111.8, 111.2, 78.6, 55.7, 55.6, 46.1, 39.8, 38.0, 36.2, 28.8, 24.9, 21.9, 2.6. ν_{max} (cm^{-1}): 2933, 2851, 1715, 1586, 1479, 1256, 1073. HRMS (ESI/TOF) m/z: [M + H]$^+$ Calculated for $C_{20}H_{33}O_4Si$ 365.2143; Found 365.2129.

(4aS,10S,11aR)-6,9-dimethoxy-1,2,3,4,5,10,11,11a-octahydro-4a,10-epoxydibenzo[a,d][7]annulene (3)

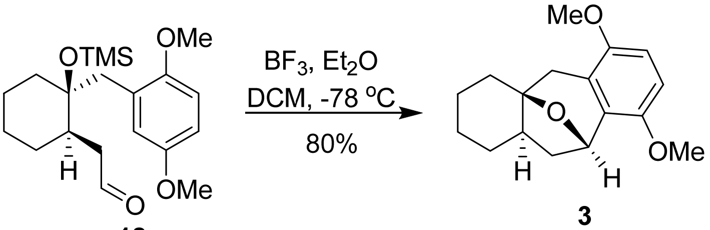

To a solution of aldehyde **18** (0.125 g, 0.34 mmol) dissolved in DCM (4 mL) was added BF_3•OEt_2 (0.08 mL, 0.69 mmol) dropwise under N_2 at -78 ºC until complete consumption of starting material was

observed by TLC analysis (2 h). The reaction was then quenched with saturated aqueous ammonium chloride. The aqueous layer was then extracted (3 × 5 mL) with EtOAc. The combined organic extracts were dried with anhydrous $MgSO_4$, filtered, and concentrated under reduced pressure. Column chromatography: 5% ethyl acetate/hexanes. Column chromatography: 5% ethyl acetate/hexanes. Yield: 80% (0.08 g, 0.28 mmol). White solid. ^1H NMR (500 MHz, $CDCl_3$) δ: 6.61 (s, 2H), 5.38 (d, *J*= 7.0 Hz, 1H), 3.764 (s, 3H), 3.761 (s, 3H), 2.73 (d, *J*= 17.5 Hz, 1H), 2.51 (d, *J*= 17.5 Hz, 1H), 2.08-2.04 (m, 1H), 2.01-1.86 (m, 3H), 1.73-162 (m, 3H), 1.58-1.53 (m, 1H), 1.29-123 (m, 2H). ^{13}C NMR (125 MHz, $CDCl_3$) δ: 151.5, 148.1, 132.4, 122.6, 107.8, 107.7, 79.3, 70.4, 56.0, 55.5, 43.4, 41.8, 38.0, 32.9, 29.9, 21.0, 19.1. IR ν_{max} (cm^{-1}): 2966, 2828, 1541, 1461, 1429, 1333, 1047, 755. HRMS (ESI/TOF) m/z: $[M]^{+\cdot}$ Calculated for $C_{17}H_{22}O_3$ 274.1569; Found 274.1542.

(4aS,10S,11aR)-1,2,3,4,5,10,11,11a-octahydro-4a,10-epoxydibenzo[a,d][7]annulene-6,9-diol (5)

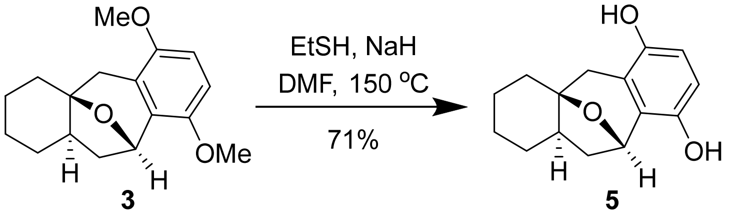

Under inert atmosphere N_2, NaH (0.11 g, 2.6 mmol, 60% in mineral oil) was washed with anhydrous hexanes (3 x 10 mL). After a few minutes, anhydrous DMF (8 mL) was added. To this mixture was slowly added a solution of EtSH (0.3 mL, 3.96 mmol) in anhydrous DMF (0.3 mL) at 0 °C and the resulting yellow solution was stirred for 20 min at rt. A solution of compound **3** (0.06 g, 0.22 mmol) in DMF (1 mL) was then added dropwise and the resulting mixture was stirred for 5 h at 150 °C. When the reaction became slightly brown, the mixture was cooled to the rt and a saturated solution of NH_4Cl (10 mL) was added. The mixture was extracted with Et_2O (3 x 10 mL). The organic phase was washed with H_2O, saturated NaCl solution and dried over anhydrous $MgSO_4$. The solvent was removed under reduced pressure. Column chromatography: 20-40% ethyl acetate/hexanes. Yield: 71% (0.04 g, 0.16 mmol). White solid. ^1H NMR (500 MHz, CD_3OD) δ: 6.47 (d, *J*= 8.5 Hz, 1H), 6.44 (d, *J*= 9.0 Hz, 1H), 5.34 (d, *J*= 7.0 Hz, 1H), 2.68 (d, *J*= 17.5 Hz, 1H), 2.53 (d, *J*= 17.5 Hz, 1H), 2.08 (ddd, *J*= 11.5, 8.0, 1.0 Hz, 1H), 2.01-1.91 (m, 3H), 1.78-1.59 (m, 6H), 1.38-1.28 (m, 2H). ^{13}C NMR (125 MHz, CD_3OD) δ: 149.1, 145.3, 131.1, 121.2, 114.1, 113.6, 81.1, 72.2, 44.3, 43.0, 39.3, 34.0, 31.1, 22.1, 20.3. IR ν_{max} (cm^{-1}): 3358, 2949, 2881, 1611, 1570, 1444, 1366, 1155, 1086, 1009, 984. HRMS (ESI/TOF) m/z: $[M + H]^+$ Calculated for $C_{15}H_{19}O_3$ 247.1329; Found 247.1341.

(4aS,10S,11aR)-1,2,3,4,5,10,11,11a-octahydro-4a,10-epoxydibenzo[a,d][7]annulene-6,9-dione (6′)

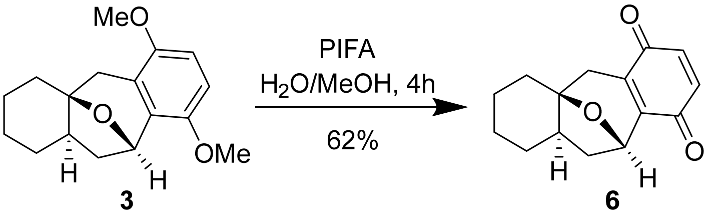

To a round bottom flask containing H_2O (0.5 mL) and MeOH (13 μL) was added compound **3** (0.03 g, 0.1 mmol) and PIFA (0.17 g, 0.4 mmol). Reaction was run at room temperature for 4 h. The mixture was extracted with EtOAC (5 mL) and dried over anhydrous $MgSO_4$. Column chromatography: 20-40% ethyl acetate/hexanes. Yield: 62% (0.0154 g, 0.065 mmol). Yellow solid. ^1H NMR (500 MHz, $CDCl_3$) δ: 6.67 (s, 2H), 5.50 (d, *J*= 5 Hz, 1H), 2.55 (d, *J*= 19.5 Hz, 1H), 2.29 (d, *J*= 19.5 Hz, 1H), 2.10 (dd, *J*= 12.0, 8 Hz, 1H), 1.97-1.87 (m, 3H), 1.71-1.52 (m, 5H), 1.26-1.22 (m, 2H). ^{13}C NMR (125 MHz, $CDCl_3$) δ: 187.1, 185.1, 146.8, 138.6, 136.3, 135.8, 79.8, 69.0, 43.1, 42.1, 37.2, 32.5, 29.9, 20.7, 18.9. ν_{max} (cm^{-1}): 2944, 2871, 1659, 1609, 1451, 1380, 1091. HRMS (ESI/TOF) m/z: $[M + H]^+$ Calculated for $C_{15}H_{17}O_3$ 245.1772; Found 245.1783.

2-(2,3-dimethoxyphenyl)propan-2-ol (36)[3]

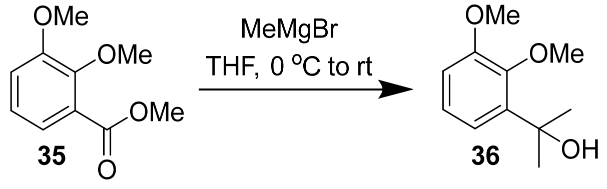

To a solution of methyl 2,3-dimethoxybenzoate (**35**) (1.76 g, 9.0 mmol) in THF (20 mL) was added dropwise MeMgBr (9.0 mL, 27 mmol, 3M solution in Et_2O) at 0°C and stirred for 5 h at rt. The reaction was quenched with NH_4Cl (20 mL). The mixture was extracted with diethyl ether (20 x 3 mL), washed by brine (20 x 2 mL), dried over anhydrous $MgSO_4$ and concentrated under reduced pressure. Column chromatography: 15-20% ethyl acetate/hexanes. Yield: 96% (1.70 g, 8.70 mmol). Viscous colorless liquid. ^1H NMR (500 MHz, $CDCl_3$) δ: 7.07 (t, *J*= 8.0 Hz, 1H), 6.94 (dd, *J*= 8.0, 1.5 Hz, 1H), 6.86 (dd, *J*= 8.0, 1.5 Hz, 1H), 4.29 (bs, 1H), 3.98 (s, 3H), 3.87 (s, 3H), 1.60 (s, 6H).

1-isopropyl-2,3-dimethoxybenzene (20)[4]

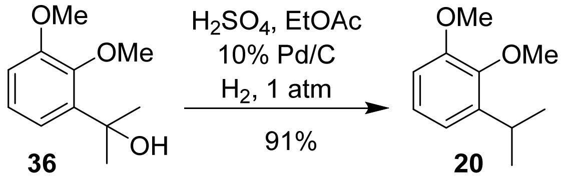

A mixture of 2-(2,3-dimethoxyphenyl)propan-2-ol (**36**) (1.50 g, 7.67 mmol), 10% Pd/C (173 mg) and H_2SO_4 (0.43 mL) in EtOAc (22 mL) was maintained under H_2 atmosphere at room temperature for 24 hours. The reaction mixture was filtered through celite and the solvent was evaporated. The residue was dissolved in diethyl ether (50 mL), washed with saturated solution of $NaHCO_3$, dried over anhydrous $MgSO_4$ and concentrated under reduced pressure. Column chromatography: 3-5% ethyl acetate/hexanes. Yield: 91% (1.25 g, 6.94 mmol). Colorless liquid. ^1H NMR (500 MHz, $CDCl_3$) δ: 7.03 (t, *J*= 8.0 Hz, 1H), 6.85 (dd, *J*= 8.0, 1.5 Hz, 1H), 6.76 (t, *J*= 8.0, 1.5 Hz, 1H), 3.86 (s, 3H), 3.82 (s, 3H), 3.36 (quint, *J*= 7.0 Hz, 1H), 1.22 (d, *J*= 7.0 Hz, 1H).

1-bromo-4-isopropyl-2,3-dimethoxybenzene (23)[5]

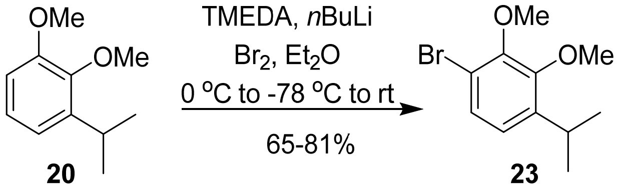

2M BuLi in hexane (4.0 mL, 8.0 mmol) was added dropwise, at 0 °C under argon, to a solution of compound **20** (1.08 g, 6.0 mmol) and TMEDA (1.2 mL, 8.0 mmol) in anhydrous Et_2O (32 ml). After stirring the resulting yellow suspension at rt for 4 h, it was cooled at -78 °C and bromine (0.38 mL, 7.0 mmol) was added. The resulting mixture was stirred at rt for 15 h and then water (5 mL) was added. The aqueous layer was extracted with Et_2O and the combined organic phase was washed with aqueous saturated $NaHSO_3$, water, brine (10 mL), dried over $MgS0_4$ and concentrated under reduced pressure. Column chromatography: 1% ethyl acetate/hexanes. Yield: 65% (1.06 g, 3.94 mmol). Colorless oil. ^{1}H NMR (500 MHz, $CDCl_3$) δ: 7.23 (d, *J*= 8.5 Hz, 1H), 6.86 (d, *J*= 8.5 Hz, 1H), 3.88 (s, 3H), 3.87 (s, 3H), 3.28 (quint, *J*= 7.0 Hz, 1H), 1.2 (d, *J*= 7.0 Hz, 6H).

(S)-2-allyl-3,3-dimethylcyclohexan-1-one (38)[4]

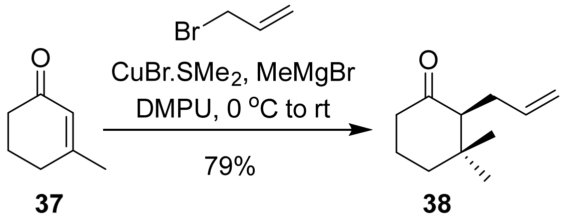

To a solution of $CuBr.SMe_2$ (0.05 g, 0.21 mmol) in dry THF (6 mL) in N_2 atmosphere was added methylmagnesium bromide (3.0M in diethyl ether, 3.0 mL, 9.0 mmol) at 0 °C. After stirring at 0 °C for 30 min, enone **37** (0.58 g, 0.6 mL, 5.29 mmol,) was added slowly. The solution was stirred at 0 °C for further 35 min, before a solution of DMPU (4.0 mL, 32.2 mmol) and allyl bromide (2.22 g, 1.6 mL, 18.5 mmol) was added dropwise at 0 °C. Stirring was continued at 0 °C for 40 min, then the mixture was allowed to reach room temperature and stirred for further 4.5 h. After quenching the reaction by addition of saturated NH_4Cl solution (15 mL), the aqueous layer was extracted with diethyl ether (3 x 15 mL). The combined organic layers were washed with saturated NH_4Cl solution (2 x 10 mL) and brine (15 mL), dried with $MgSO_4$, filtered, and concentrated in vacuo. Column chromatography: 3-5% ethyl acetate/hexanes. Yield: 79% (0.69 g, 4.18 mmol). Colorless oil. ^{1}H NMR (400 MHz, $CDCl_3$) δ: 5.80-5.70 (m, 1H), 5.01-4.89 (m, 2H), 2.48-2.40 (m, 1H), 2.34-2.20 (m, 3H), 2.09-2.02 (m, 1H), 1.92-1.77 (m, 2H), 1.68-1.56 (m, 2H), 1.06 (s, 3H), 0.78 (s, 3H).

(3R,4S)-4-allyl-5,5-dimethyl-1-oxaspiro[2.5]octane (39)[4]

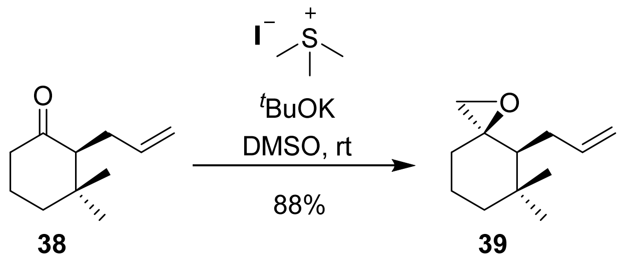

38 39

Trimethylsulfonium iodide (2.41 g, 11.8 mmol) was added to dry DMSO (25 mL) and stirred until complete dissolution of the salt. Addition of allyl cyclohexanone **38** (1.18 g, 7.15 mmol), followed by addition of solution of potassium *tert*-butoxide (1.33 g, 11.8 mmol) in DMSO (17 mL). The resulting solution was kept under stirring at room temperature for 2 hours. After this time, H_2O (25 mL) was added, the aqueous phase was extracted with ethyl ether (3 x 20 ml), washed with H_2O (17 ml), dried over anhydrous $MgSO_4$ and the solvent was evaporated. Column chromatography: 3% ethyl acetate/hexanes. Yield: 88% (1.14 g, 6.33 mmol). Colorless oil. ^1H NMR (400 MHz, $CDCl_3$) δ: 5.91-5.81 (m, 1H), 5.03-4.92 (m, 2H), 2.66 (dd, *J*= 4.8, 0.8 Hz, 1H), 2.49 (d, *J*= 4.8, 1H), 2.23-2.16 (m, 1H), 2.14-2.05 (m, 1H), 1.77-1.67 (m, 1H), 1.66-1.56 (m, 2H), 1.53-1.45 (m, 1H), 1.39-1.34 (m, 1H), 1.29-1.23 (m, 1H), 1.02 (s, 3H), 0.92 (s, 3H).

2-((3R,4S)-5,5-dimethyl-1-oxaspiro[2.5]octan-4-yl)acetaldehyde (19)

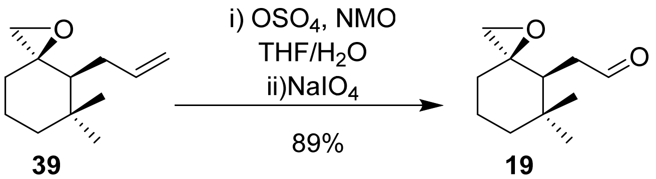

39 19

To a solution of epoxy-alkene **19** (0.47 g, 2.6 mmol) and NMO (0.61 g, 5.2 mmol) in THF/H_2O (3:1, 40 mL) under stirring at room temperature was added of 0.15 M OsO_4 (0.34 mL, 2.0 mol%) and allowed to stir for overnight. After addition of $NaIO_4$ (1.16 g, 5.4 mmol) the mixture stirred at room temperature for further 2 hours. To this reaction mixture 10% $Na_2S_2O_3$ (10 mL) solution was added and extracted with diethyl ether (3 x 10). The combined organic phase was washed with H_2O (10 mL) and saturated NaCl solution (10 mL), dried over anhydrous MgSO4 and the solvent was evaporated. Column chromatography: 5-8% ethyl acetate/hexanes. Yield: 89% (0.43 g, 2.36 mmol). colourless oil. ^1H NMR (500 MHz, $CDCl_3$) δ: 9.71 (t, *J*= 2.0 Hz, 1H), 2.54 (dd, *J*= 16.0, 4.5 Hz, 2H), 2.45 (td, *J*= 4.0, 1.5Hz, 2H), 1.91 (t, *J*= 6.0 Hz, 1H), 1.71 (m, 1H), 1.59 (m, 2H), 1.40 (m, 2H), 1.29 (m, 1H), 1.00 (s, 3H), 0.85 (s, 3H). ^{13}C NMR (125 MHz, $CDCl_3$) δ: 202.2, 59.2, 54.8, 44.1, 41.3, 36.2, 35.3, 31.3, 28.4, 26.2, 20.6. IR ν_{max} (cm^{-1}): 3043, 2929, 2868, 2724, 1721, 1455, 1410, 1389, 1366, 1299, 1268, 1210, 1172, 1148, 950, 911, 826. HRMS (ESI/TOF) m/z: $[M + CH_3CN + H]^+$ Calculated for $C_{13}H_{22}NO_2$ 224.1645; Found 224.1623.

(3aS,7aS)-7a-(4-isopropyl-2,3-dimethoxybenzyl)-4,4-dimethyloctahydrobenzofuran-2-ol (40)

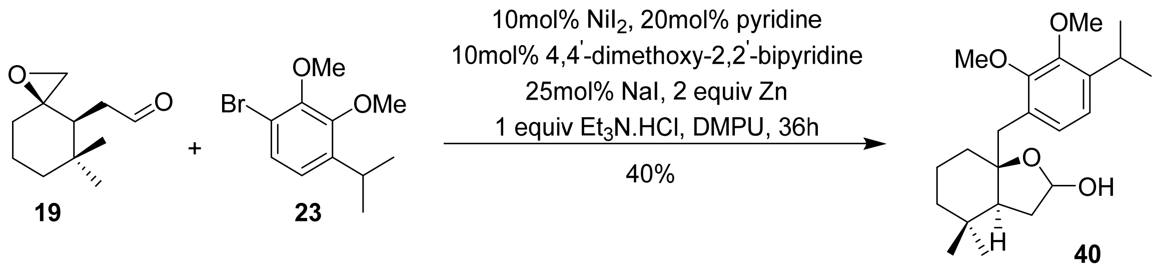

To an oven-dried 1-dram vial containing a teflon-coated stir-bar was sequentially added: triethylamine hydrohalide (0.07 g, 0.50 mmol), Zn^0 dust (0.065 g, 1.0 mmol), nickel pre-catalyst (16 mg of NiI_2, 10 mol%), 4,4′-diemthoxy-2,2′-bipyridine ligand (0.011 g, 0.05 mmol, 10 mol%), sodium iodide (0.02 g, 0.125 mmol), DMPU (2.5 mL), pyridine (10 µL, 0.1 mmol), aryl bromide **23** (0.55 mmol in three portion: 3 x 0.275 mmol at 0 h, 3 h and 6 h) (0.047 g, 0.275 mmol), and epoxide **19** (0.091 g, 0.5 mmol). The reaction vials were capped with a PTFE-faced silicone septum, and the head space of the vial was purged vigorously with nitrogen gas for one minute, then stirred (800 RPM) in a reaction block on the benchtop at room temperature. After 3 h and 6 h of reaction time, second and third portion of aryl halide **23** (each time 0.047 g, 0.275 mmol, dissolved in 0.25 mL of DMPU) was transferred via syringe. After 36 h, the reaction mixture was poured into 50 mL of 0.1 M pH 8 phosphate buffer. This aqueous mixture was then extracted with diethyl ether (3 × 50 mL). The organic layers were combined, washed with 50 mL of brine, and dried over anhydrous Na_2SO_4. After decantation of the organic layer, volatile materials were removed on a rotary evaporator. Column chromatography: 5-10% ethyl acetate/hexanes. Yield: recovered epoxide **10,** 47% (0.043 mg, 0.24 mmol) and acetal **40**, 40% (0.058 g, 0.26 mmol). Colorless oil. ^{1}H NMR (500 MHz, $CDCl_3$) δ: 7.02 (d, *J*= 8.0 Hz, 1H), 6.92 (d, *J*= 8.0 Hz, 1H), 5.31 (Brs 1H), 3.85 (s, 3H), 3.84 (s, 3H), 3.30 (quint, *J*= 7.0 Hz, 1H), 3.15 (d, *J*= 13.5 Hz, 1H), 3.00 (Brs 1H), 2.88 (d, *J*= 13.5 Hz, 1H), 2.17 (dd, *J*= 11.0, 8.5 Hz, 1H), 1.97-1.94 (m, 2H), 1.80-1.74 (m, 2H), 1.56-1.48 (m, 2H), 1.36-1.28 (m, 2H), 1.22 (d, *J*= 7.0 Hz, 3H), 1.21 (d, *J*= 7.0 Hz, 3H), 1.17 (s, 3H), 0.96 (s, 3H). ^{13}C NMR (125 MHz, $CDCl_3$) δ: 151.7, 150.4, 141.2, 129.6, 127.0, 120.7, 96.3, 84.8, 60.7, 60.0, 50.7, 40.8, 37.7, 35.1, 33.5, 32.0, 30.9, 29.7, 26.9, 23.8, 23.6, 19.3. IR ν_{max} (cm^{-1}): 3398, 2956, 2931, 2868, 1490, 1455, 1411, 1364, 1332, 1274, 1250, 1214, 1153, 1050, 1022, 956, 852, 821. HRMS (ESI/TOF) m/z: $[M + Na]^+$ Calculated for $C_{22}H_{34}O_4Na$ 385.2349; Found 385.2335.

(4aS,10S,11aS)-8-isopropyl-6,7-dimethoxy-1,1-dimethyl-1,2,3,4,5,10,11,11a-octahydro-4a,10-epoxydibenzo[a,d][7]annulene (7)[6]

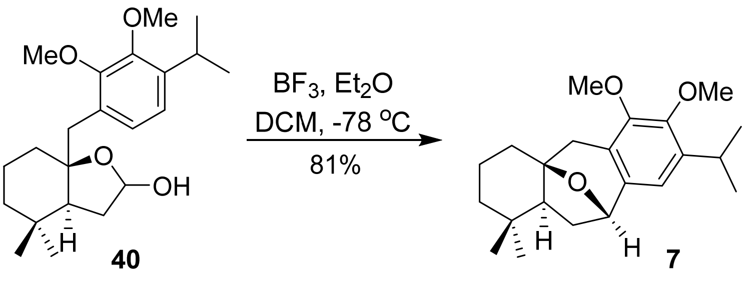

To a solution of acetal **40** (0.217 g, 0.60 mmol) dissolved in DCM (10 mL) was added BF_3•OEt_2 (0.15 mL, 1.2 mmol) dropwise under argon at -78 ºC until complete consumption of starting material was observed by TLC analysis (1.5 h). The reaction was then quenched with saturated aqueous ammonium chloride. The aqueous layer was then extracted (3 × 5 mL) with EtOAc. The combined organic extracts were dried with anhydrous MgSO4, filtered, and concentrated under reduced pressure. Column chromatography: 5% ethyl acetate/hexanes. Yield: 81% (0.17 g, 0.49 mmol). White solid. ^1H NMR (500 MHz, $CDCl_3$) δ: 6.63 (s, 1H), 4.85 (d, *J*= 6.5 Hz, 1H), 3.82 (s, 3H), 3.81 (s, 3H). 3.27 (quint, *J*= 7.0 Hz, 1H), 2.78 (d, *J*= 17.0 Hz, 1H), 2.49 (d, *J*= 17.0 Hz, 1H), 2.11 (td, *J*= 12.0, 7.0 Hz, 1H), 2.00 (dd, *J*= 7.5, 3.0 Hz, 1H), 1.91 (ddd, *J*= 12.0, 8.5, 0.5 Hz, 1H), 1.83-1.77 (m, 3H), 1.65-1.60 (m, 1H), 1.52 (m, 1H), 1.20 (d, *J*= 7.0 Hz, 3H), 1.19 (d, *J*= 7.0 Hz, 3H), 1.19-1.13 (m, 1H), 0.96 (s, 3H), 0.84 (s, 3H). ^{13}C NMR (125 MHz, $CDCl_3$) δ: 151.0, 148.9, 140.1, 137.9, 124.2, 116.6, 80.2, 76.1, 60.8, 59.8, 51.1, 39.7, 39.5, 32.3, 32.0, 30.8, 30.7, 26.9, 26.8, 23.9, 23.7, 16.3.[6]

(4aS,10S,11aS)-8-isopropyl-1,1-dimethyl-1,2,3,4,5,10,11,11a-octahydro-4a,10-epoxydibenzo[a,d][7]annulene-6,7-diol (1')[6,7]

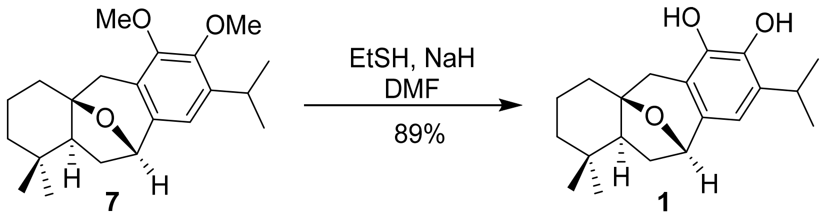

Under inert atmosphere N_2, NaH (0.04 g, 1.0 mmol, 60% in mineral oil) was washed with anhydrous hexanes (3 x 20 mL). After a few minutes, anhydrous DMF (3 mL) was added. To this mixture was slowly added a solution of EtSH (0.09 g, 0.11 mL, 1.48 mmol) in anhydrous DMF (0.1 mL) at 0 °C and the resulting yellow solution was stirred for 20 min at rt. A solution of compound **7** (0.028 g, 0.082 mmol) in DMF (2 mL) was then added dropwise and the resulting mixture was stirred for 5 h at 150 °C. When the reaction became slightly brown, the mixture was cooled to the rt and a saturated solution of NH_4Cl (5 mL) was added. The mixture was extracted with Et_2O (3 x 5 mL). The organic phase was washed with H_2O, saturated NaCl solution and dried over anhydrous $MgSO_4$. The solvent was removed under reduced pressure. Column chromatography: 20-40% ethyl acetate/hexanes.

Compound **1** yield: 89% (0.023 g, 0.073 mmol). White solid. ^{1}H NMR (400 MHz, $CDCl_3$) δ: 6.44 (s, 1H), 5.21 (s, 1H), 5.11 (s, 1H), 4.85 (d, *J*= 6.8 Hz, 1H), 3.11 (septet, *J*= 6.8 Hz, 1H), 2.71 (d, *J*= 16.4 Hz, 1H), 2.38 (d, *J*= 16.4 Hz, 1H), 2.11 (dt, *J*= 12.0, 6.8 Hz, 1H), 2.03-1.95 (m, 1H), 1.89 (dd, *J*= 12.0, 8.4 Hz, 1H), 1.83-1.73 (m, 3H), 1.65-1.57 (m, 1H), 1.55-1.47 (m, 1H), 1.23 (d, *J*= 6.8 Hz, 3H), 1.22 (d, *J*= 6.8 Hz, 3H), 1.20-1.12 (m, 1H), 0.96 (s, 3H), 0.84 (s, 3H). ^{13}C NMR (100 MHz, $CDCl_3$) δ: 141.7, 139.7, 134.3, 132.1, 116.7, 112.9, 80.3, 76.3, 51.2, 39.8, 38.9, 32.3, 32.0, 30.81, 30.76, 27.3, 26.8, 22.9, 22.8, 16.3.

(4aS,10S,11aS)-9-bromo-8-isopropyl-6,7-dimethoxy-1,1-dimethyl-1,2,3,4,5,10,11,11a-octahydro-4a,10-epoxydibenzo[a,d][7]annulene (10)

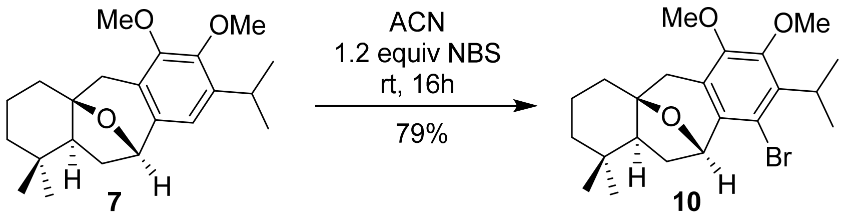

N-bromosuccinimide (NBS) (0.032 g, 0.18 mmol) was added to an MeCN solution (1.5 mL) of icetaxane **7** (0.051 g, 0.15 mmol) and the resulting mixture was stirred at room temperature for 16 h[8]. After evaporation of MeCN under reduced pressure, the residue was dissolved in CH_2Cl_2 and treated with a saturated solution of $NaHCO_3$. The mixture was extracted with CH_2Cl_2 (3 x 5 mL). The organic phase was washed with H_2O and dried over anhydrous $MgSO_4$. The solvent was removed under reduced pressure. Column chromatography: 2-4% ethyl acetate/hexanes. Compound **10** yield: 79% (0.051 g, 0.012 mmol). White solid. ^{1}H NMR (500 MHz, $CDCl_3$) δ: 5.30 (d, *J*= 6.5 Hz, 1H), 3.84 (s, 3H), 3.78 (s, 3H), 3.68-3.61 (m, 1H), 2.77 (d, *J*= 17.0 Hz, 1H), 2.45 (d, *J*= 17 Hz, 1H), 2.12 (dt, *J*= 13.5, 7.0 Hz, 1H), 1.99-1.94 (m, 2H), 1.88-1.79 (m, 2H), 1.73 (t, *J*= 8.0 Hz, 1H), 1.66-1.60 (m, 1H), 1.56-1.50 (m, 1H), 1.34 (d, *J*= 7.5 Hz, 3H), 1.32 (d, *J*= 7.5 Hz, 3H), 1.19-1.16 (m, 1H), 0.97 (s, 3H), 0.85 (s, 3H). ^{13}C NMR (125 MHz, $CDCl_3$) δ: 151.4, 150.9, 138.1, 136.7, 126.8, 116.4, 80.2, 76.4, 60.4, 59.3, 50.7, 39.1, 38.2, 31.8, 31.6, 30.5, 30.1, 27.0, 21.2, 21.0, 16.0. IR ν_{max} (cm^{-1}): 2961, 2938, 2873, 1619, 1449, 1411, 1332, 1255, 1062, 1055, 1018, 809, 649. HRMS (ESI/TOF) m/z: [M + H]$^+$ Calculated for $C_{22}H_{32}BrO_3$ 423.1529; Found 423.1515.

(4aS,10S,11aS)-8-isopropyl-6,7-dimethoxy-1,1-dimethyl-9-phenyl-1,2,3,4,5,10,11,11a-octahydro-4a,10-epoxydibenzo[a,d][7]annulene (11)

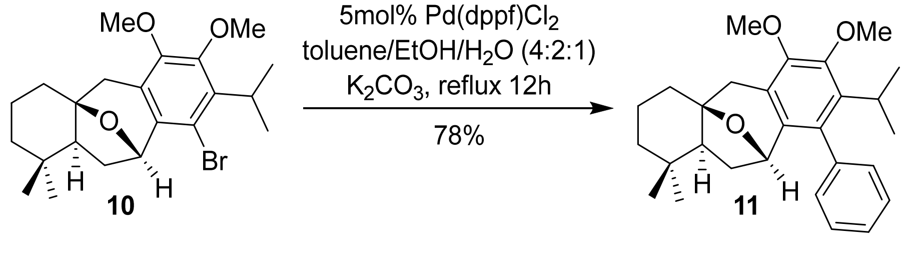

To a stir solution of Bromo-icetexane **10** (0.002 g, 0.047 mmol) and phenyl boronic acid (0.0087 g, 0.07 mmol) in toluene/ethanol/water (4:2:1) (1.5 mL) was added K_2CO_3 (0.02 g, 0.14 mmol). The solution was stirred and degassed with argon three times, and catalyst (dppf)$PdCl_2$ (0.0025 g, 5% mol.) was added[9]. The dark solution was warmed to reflux for 16 h with the total consumption of starting material. Then the solution was cooled to room temperature, water (2 mL) was added, and the solution was extracted with EtOAc (3 x 2 mL). The organic phase was washed with H_2O, brine and dried over anhydrous $MgSO_4$. The solvent was removed under reduced pressure. Column chromatography: 2-4% ethyl acetate/hexanes. Compound **11** yield: 78% (0.015 g, 0.037 mmol). White solid. ^1H NMR (400 MHz, $CDCl_3$) δ: 7.40-7.30 (m, 3H), 7.19-7.16 (m, 1H), 7.06-7.03 (m, 1H), 4.42 (t, *J*= 4.0 Hz, 1H), 3.89 (s, 1H), 3.84 (s, 1H), 2.84 (d, *J*= 17.2 Hz, 1H), 2.64 (quint, *J*= 7.2 Hz, 1H), 2.52 (d, *J*= 17.2 Hz, 1H), 1.20-1.95 (m, 1H), 1.85-1.82 (m, 2H), 1.80-1.68 (m, 3H), 1.57-1.51 (m, 1H), 1.42 (dd, *J*= 13.6, 6.8 Hz, 1H), 1.21-1.10 (m, 1H), 1.16 (d, *J*= 7.2 Hz, 1H), 1.15 (d, *J*= 7.2 Hz, 1H), 0.92 (m, 3H), 0.75 (m, 3H). ^{13}C NMR (100 MHz, $CDCl_3$) δ: 150.7, 150.6, 139.7, 137.1, 136.0, 132.8, 129.9, 129.3, 128.2, 127.9, 126.7, 124.5, 80.0, 73.6, 60.0, 59.3, 51.3, 39.4, 38.9, 33.0, 31.9, 31.1, 30.7, 30.2, 26.2, 21.9, 21.8, 16.4. IR ν_{max} (cm^{-1}): 2959, 2929, 2877, 1609, 1432, 1413, 1339, 1256, 1064, 1055, 1009, 819. HRMS (ESI/TOF) m/z: $[M + H]^+$ Calculated for $C_{28}H_{37}O_3$ 421.2737; Found 421.2747.

2-(2,3,6-trimethoxyphenyl)propan-2-ol (41)[10]

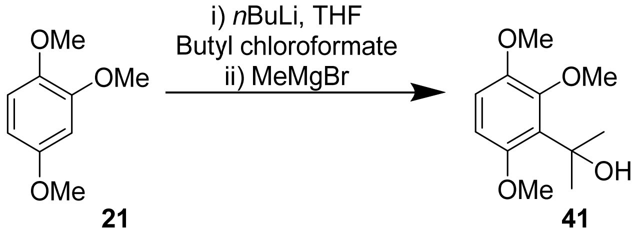

In an oven dried flask 1,2,4-Trimethoxybenzene **21** (0.672 g, 4.0 mmol) was dissolved in anhydrous THF (10 mL). To this mixture *n*-Butyllithium (2.2 mL, 4.4 mmol, 2M in hexanes) was slowly added at 0 °C and the reaction mixture was allowed to warm to rt. After 2 h, the mixture was cooled to 0 °C and a solution of buthyl chloroformate (0.62 mL, 4.8 mmol) in THF (2 mL) was transferred. The resulting mixture was stirred at rt for 4 h. After 4 h it was cooled once again to 0 °C and 3 M methylmagnesium bromide solution in diethyl ether (5.3 mL, 16.0 mmol, 3.0 equiv) was gradually transferred. The resulting mixture was allowed to for further 5 h at rt and then quenched by the dropwise addition of saturated aqueous NH_4Cl (15 mL) followed by water (15 mL). The aqueous layer was extracted with Et_2O (3 x 25 mL) and the combined organic phase was washed with water, brine (10 mL), dried over $MgSO_4$ and concentrated under reduced pressure. Column chromatography: 10-15% ethyl acetate/hexanes. Yield: 86% (0.78 g, 3.45 mmol). Colorless oil. ^1H NMR (500 MHz, $CDCl_3$) δ: 6.77 (d, *J*= 9.0 Hz, 1H), 6.64 (d, *J*= 9.0 Hz, 1H), 5.89 (s, 1H), 3.85 (s, 3H), 3.82 (s, 6H), 1.67 (s, 6H).

2-isopropyl-1,3,4-trimethoxybenzene (42)[10]

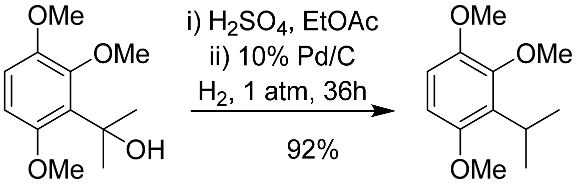

41 **21**

A mixture of 2-(2,3,6-trimethoxyphenyl)propan-2-ol **41** (2.35 g, 10.4 mmol), 10% Pd/C (0.234 g) and H_2SO_4 (0.59 mL) in EtOAc (30 mL) was maintained under H_2 atmosphere at room temperature for 24 hours. The reaction mixture was filtered through celite and the solvent was evaporated. The residue was dissolved in diethyl ether (50 mL), washed with saturated solution of $NaHCO_3$, dried over anhydrous $MgSO_4$ and concentrated under reduced pressure. Column chromatography: 3-5% ethyl acetate/hexanes. Yield: 92% (2.02 g, 9.62 mmol). Colorless liquid. ^{1}H NMR (500 MHz, $CDCl_3$) δ: 6.70 (d, *J*= 9.0 Hz, 1H), 6.55 (d, *J*= 9.0 Hz, 1H), 3.81 (s, 3H), 3.80 (s, 3H), 3.76 (s, 3H), 3.54 (quint, *J*= 7.5 Hz, 1H), 1.31 (d, *J*= 7.5 Hz, 6H).

1-bromo-4-isopropyl-2,3,5-trimethoxybenzene (24)

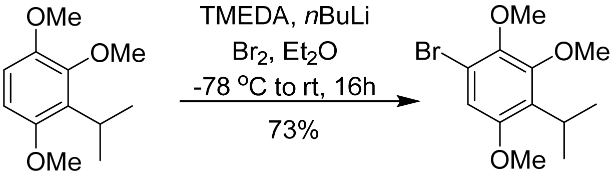

21 **24**

2M BuLi in hexane (1.65 mL, 3.3 mmol) was added dropwise, at 0 °C under argon, to a solution of compound **42** (0.63 g, 3.0 mmol) and TMEDA (0.5 mL, 3.3 mmol) in anhydrous Et_20 (18 ml). After stirring the resulting yellow suspension at rt for 4 h, it was cooled at -78 °C and bromine (0.19 mL, 3.5 mmol) was added. The resulting mixture was stirred at rt for 15 h and then water (5 mL) was added. The aqueous layer was extracted with Et_2O and the combined organic phase was washed with aqueous saturated $NaHSO_3$, water, brine (10 mL), dried over $MgS0_4$ and concentrated under reduced pressure. Column chromatography: 1% ethyl acetate/hexanes. Yield: 73% (0.63 g, 2.19 mmol). Yellowish Colorless oil. ^{1}H NMR (500 MHz, $CDCl_3$) δ: 6.76 (s, 1H), 3.84 (s, 3H), 3.82 (s, 3H), 3.76 (s, 3H), 3.47 (quint, *J*= 7.0 Hz, 1H), 1.28 (d, *J*= 7.0 Hz, 1H). ^{13}C NMR (125 MHz, $CDCl_3$) δ: 155.2, 152.7, 145.0, 130.5, 114.1, 110.8, 61.3, 60.7, 56.0, 25.4, 21.1. IR ν_{max} (cm^{-1}): 2980, 2955, 2934, 1575, 1460, 1430, 1390, 1375, 1340, 1277, 1228, 1205, 1170, 1033, 1071, 1053, 1011, 922, 849, 790. HRMS (ESI/TOF) m/z: $[M + H]^+$ Calculated for $C_{12}H_{18}BrO_3$ 289.0434; Found 289.0453.

(3aS,7aS)-7a-(4-isopropyl-2,3,5-trimethoxybenzyl)-4,4-dimethyloctahydrobenzofuran-2-ol (42)

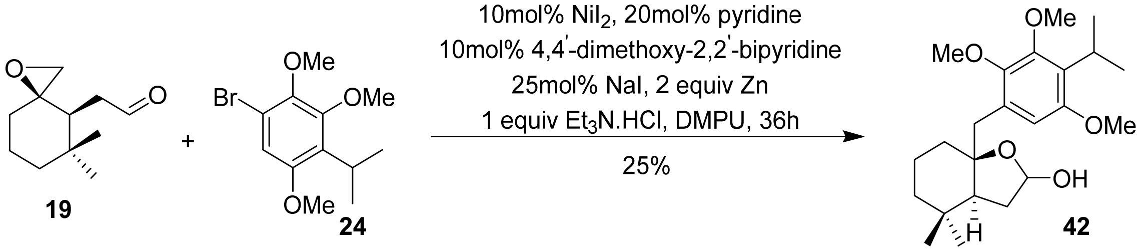

To an oven-dried 1-dram vial containing a teflon-coated stir-bar was sequentially added: triethylamine hydrohalide (0.07 g, 0.50 mmol), Zn^0 dust (0.065 g, 1.0 mmol), nickel pre-catalyst (32 mg of NiI_2, 20 mol%), 4 4'-dimethoxy-2 2'-bipyridine (0.011 g, 0.05 mmol, 10 mol%), sodium iodide (0.02 g, 0.125 mmol), DMPU (3 mL), pyridine (10 μL, 0.1 mmol), aryl bromide **24** (0.144 g, 0.5 mmol), and epoxide **19** (0.118 g, 0.65 mmol). The reaction vials were capped with a PTFE-faced silicone septum, and the head space of the vial was purged vigorously with nitrogen gas for one minute, then stirred (800 RPM) in a reaction block on the benchtop at room temperature for 36 h. After 36 h reaction time, the reaction mixture was poured into 50 mL of 0.1 M pH 8 phosphate buffer. This aqueous mixture was then extracted with diethyl ether (3 × 50 mL). The organic layers were combined, washed with 50 mL of brine, and dried over anhydrous Na_2SO_4. After decantation of the organic layer, volatile materials were removed on a rotary evaporator. Column chromatography: 5-10% ethyl acetate/hexanes. Yield: 25% (0.048 g, 0.124 mmol). Colorless oil. ^{1}H NMR (400 MHz, $CDCl_3$) δ: 6.56 (s, 1H), 5.31 (Brs 1H), 3.80 (s, 3H), 3.78 (s, 3H), 3.76 (s, 3H), 3.47 (quint, *J*= 6.8 Hz, 1H), 3.07 (d, *J*= 13.6 Hz, 1H), 3.01 (d, *J*= 2.8 Hz, 1H), 2.90 (d, *J*= 13.2 Hz, 1H), 2.15 (dd, *J*= 11.6, 7.6 Hz, 1H), 1.98-1.93 (m, 2H), 1.79 (td, *J*= 13.2, 4.0 Hz, 1H), 1.58-1.47 (m, 3H), 1.40-1.32 (m, 1H), 1.31-1.26 (m, 1H), 1.29 (d, *J*= 7.2 Hz, 3H), 1.286 (d, *J*= 7.2 Hz, 3H), 1.14 (s, 3H), 0.95 (s, 3H). ^{13}C NMR (125 MHz, $CDCl_3$) δ: 154.1, 151.6, 146.1, 129.0, 128.98, 109.9, 96.4, 84.9, 60.9, 60.2, 55.9, 50.6, 41.0, 37.8, 35.0, 33.8, 32.0, 30.9, 29.7, 25.3, 21.46, 21.45, 19.3. IR ν_{max} (cm^{-1}): 3417, 2945, 2913, 2871, 1496, 1463, 1433, 1358, 1320, 1284, 1249, 1219, 1157, 1044, 1028, 945, 856, 817. HRMS (ESI/TOF) m/z: [M + Na]$^+$ Calculated for $C_{23}H_{36}O_5Na$ 415.2455; Found 415.2414.

(4aS,10S,11aS)-8-isopropyl-6,7,9-trimethoxy-1,1-dimethyl-1,2,3,4,5,10,11,11a-octahydro-4a,10-epoxydibenzo[a,d][7]annulene (8)

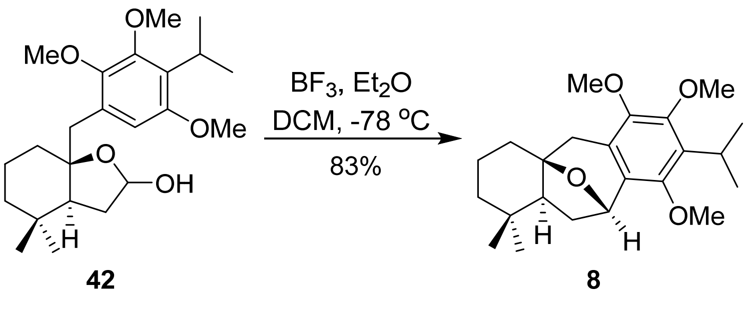

To a solution of acetal **42** (0.04 g, 0.10 mmol) dissolved in DCM (2 mL) was added BF_3•OEt_2 (0.025 mL, 0.003 mmol) dropwise under argon at -78 ºC until complete consumption of starting material was observed by TLC analysis (1.5 h). The reaction was then quenched with saturated aqueous ammonium chloride. The aqueous layer was then extracted (3 × 1 mL) with EtOAc. The combined organic extracts were dried with anhydrous $MgSO_4$, filtered, and concentrated under reduced pressure. Column chromatography: 5% ethyl acetate/hexanes. Yield: 83% (0.031 g, 0.083 mmol). Colorless viscous liquid. ^{1}H NMR (500 MHz, $CDCl_3$) δ: 5.29 (d, 7.5 Hz, 1H), 3.83 (s, 3H), 3.76 (s, 3H), 3.69 (s, 3H). 3.35 (heptet, *J*= 7.5 Hz, 1H), 2.77 (d, *J*= 17.5 Hz, 1H), 2.44 (d, *J*= 17.5 Hz, 1H), 2.17-2.13 (m, 1H), 2.04-1.98 (m, 1H), 1.84 (d, *J*= 15 Hz, 1H), 1.67-1.60 (m, 1H), 1.47 (dd, 12.5, 3.0 Hz, 1H), 1.46 (d, *J*= 14.5 Hz, 1H), 1.37-1.35 (m, 1H), 1.32 (d, *J*= 7.5 Hz, 3H), 1.31 (d, *J*= 7.5 Hz, 3H), 1.21-1.16 (m, 1H), 1.09 (s, 3H), 0.97 (s, 3H). ^{13}C NMR (100 MHz, $CDCl_3$) δ: 151.0, 148.3, 147.8, 132.0, 131.3, 124.7, 80.5, 70.8, 62.3, 60.2, 59.4, 46.4, 43.5, 41.7, 38.6, 35.3, 33.0, 29.2, 28.9, 27.7, 25.6, 22.2, 22.1. HRMS (ESI/TOF) m/z: $[M]^{+\cdot}$ Calculated for $C_{23}H_{34}O_4$ 374.2457; Found 374.2487.

1-bromo-2,3-dimethoxybenzene (25)[11]

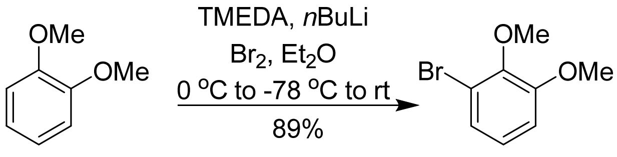

22 **25**

2.0 M BuLi in hexane (6.70 mL, 13.3 mmol) was added dropwise, at 0 °C under argon, to a solution of 1,2-Dimethylbenzene **22** (1.38 g, 10.0 mmol) and TMEDA (2.0 mL, 13.3 mmol) in anhydrous Et_2O (50 ml). After stirring the resulting yellow suspension at rt for 4 h, it was cooled at -78 °C and bromine (0.62 mL, 11.7 mmol) was added. The resulting mixture was stirred at rt for 15 h and then water (5 mL) was added. The aqueous layer was extracted with Et_2O and the combined organic phase was washed with aqueous saturated $NaHSO_3$, water, brine (10 mL), dried over $MgS0_4$ and concentrated under reduced pressure. Column chromatography: 1% ethyl acetate/hexanes. Yield: 89% (1.94 g, 8.96 mmol). Colorless oil. ^{1}H NMR (500 MHz, $CDCl_3$) δ: 7.13 (dd, *J*= 8.0, 1.5 Hz, 1H), 6.93 (t, *J*= 8.0 Hz, 1H), 6.86 (dd, *J*= 8.0, 1.5 Hz, 1H) 3.87 (s, 3H), 3.86 (s, 3H).

(3aS,7aS)-7a-(2,3-dimethoxybenzyl)-4,4-dimethyloctahydrobenzofuran-2-ol (43)

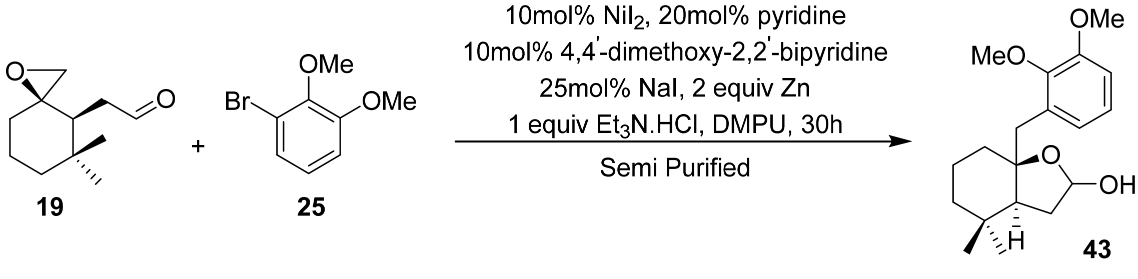

19 **25** **43**

To an oven-dried 1-dram vial containing a teflon-coated stir-bar was sequentially added: triethylamine hydrohalide (0.07 g, 0.50 mmol), Zn^0 dust (0.065 g, 1.0 mmol), nickel pre-catalyst (16 mg of NiI_2, 10 mol%), 4,4′-diemthoxy-2,2′-bipyridine ligand (0.011 g, 0.05 mmol, 10 mol%), sodium iodide (0.02 g, 0.125 mmol), DMPU (2.5 mL), pyridine (10 µL, 0.1 mmol), aryl bromide **25** (0.11 g, 0.5 mmol), and epoxide **19** (0.119 g, 0.65 mmol). The reaction vials were capped with a PTFE-faced silicone septum, and the head space of the vial was purged vigorously with nitrogen gas for one minute, then stirred (800 RPM) in a reaction block on the benchtop at room temperature. After 30 h, the reaction mixture was poured into 50 mL of 0.1 M pH 8 phosphate buffer. This aqueous mixture was then extracted with diethyl ether (3 × 50 mL). The organic layers were combined, washed with 50 mL of brine, and dried over anhydrous Na_2SO_4. After decantation of the organic layer, volatile materials were removed on a rotary evaporator. Column chromatography in 5-15% ethyl acetate/hexanes provided semi pure product **43** which is used in next step.

(4aS,10S,11aS)-6,7-dimethoxy-1,1-dimethyl-1,2,3,4,5,10,11,11a-octahydro-4a,10-epoxydibenzo[a,d][7]annulene (9)

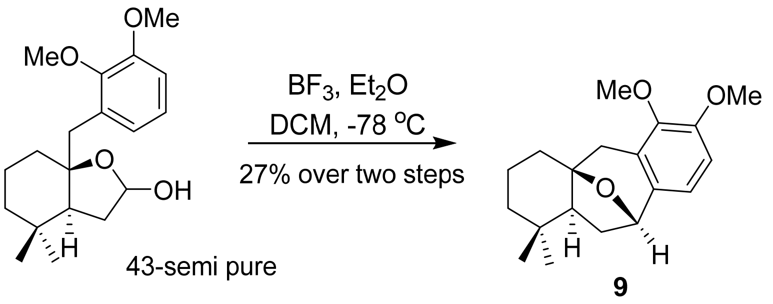

To a solution of semi pure acetal **44** (0.07 g) dissolved in DCM (4 mL) was added BF_3•OEt_2 (0.038 mL, 0.3 mmol) dropwise under argon at -78 °C until complete consumption of starting material was observed by TLC analysis (2 h). The reaction was then quenched with saturated aqueous ammonium chloride. The aqueous layer was then extracted (3 × 2 mL) with EtOAc. The combined organic extracts were dried with anhydrous $MgSO_4$, filtered, and concentrated under reduced pressure. Column chromatography: 5% ethyl acetate/hexanes. Yield over two steps: 27% (0.04 g, 0.14 mmol). White solid. ^1H NMR (400 MHz, $CDCl_3$) δ: 6.69 (d, *J*= 8.4 Hz, 1H), 6.66 (d, *J*= 8.4 Hz, 1H), 4.93 (d, *J*= 6.8 Hz, 1H), 3.82 (s, 3H), 3.78 (s, 3H). 2.80 (d, *J*= 17.6 Hz, 1H), 2.53 (d, *J*= 17.6 Hz, 1H), 2.10-1.91 (m, 3H), 1.83 (d, *J*= 14.8 Hz, 1H), 1.68-1.62 (m, 1H), 1.46 (d, *J*= 6.8 Hz, 1H), 1-46-1.42 (m, 1H), 1.34-1.31 (m, 1H), 1.20-1.13 (m, 1H), 1.08 (s, 3H), 0.96 (s, 3H). ^{13}C NMR (125 MHz, $CDCl_3$) δ: 151.1, 147.0, 135.8, 126.5, 118.4, 109.5, 80.6, 75.7, 59.8, 55.7, 46.2, 43.9, 41.2, 38.9, 35.0, 33.0, 29.3, 29.1, 27.5. IR ν_{max} (cm^{-1}): 2961, 2835, 1554, 1441, 1419, 1321, 1051, 741. HRMS (ESI/TOF) m/z: $[M + H]^+$ Calculated for $C_{19}H_{27}O_3$ 303.1955; Found 303.1927.

1-isopropyl-2,3-dimethoxy-4-methylbenzene (28)[4]

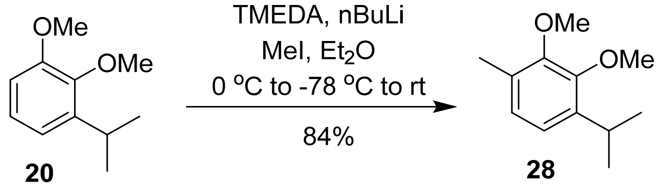

1.3 M BuLi in hexane (3.46 mL, 4.5 mmol) was added dropwise, at 0 °C under argon, to a solution of 1-isopropyl-2,3-dimethoxybenzene **20** (0.54 g, 3.0 mmol) and TMEDA (0.7 mL, 4.5 mmol) in anhydrous Et_2O (10 ml). After stirring the resulting yellow suspension at rt for 4 h, it was cooled at -78 °C and MeI (0.38 mL, 6.0 mmol) was added. The resulting mixture was stirred at rt for 3 h and then water (5 mL) was added. The aqueous layer was extracted with Et_2O and the combined organic phase was washed with aqueous saturated $NaHSO_3$, water, brine (10 mL), dried over $MgS0_4$ and concentrated under reduced pressure. Column chromatography: 1% ethyl acetate/hexanes. Yield: 84% (0.49 g, 2.53 mmol). Colorless oil. ^{1}H NMR (500 MHz, $CDCl_3$) δ: 6.89 (s, 1H), 3.86 (s, 3H), 3.84 (s, 3H), 3.30 (quint, *J*= 7.0 Hz, 1H), 2.25 (s, 3H), 1.22 (d, *J*= 7.0 Hz, 6H).

(1S,2R)-2-allyl-1-(4-isopropyl-2,3-dimethoxybenzyl)cyclohexan-1-ol (30)

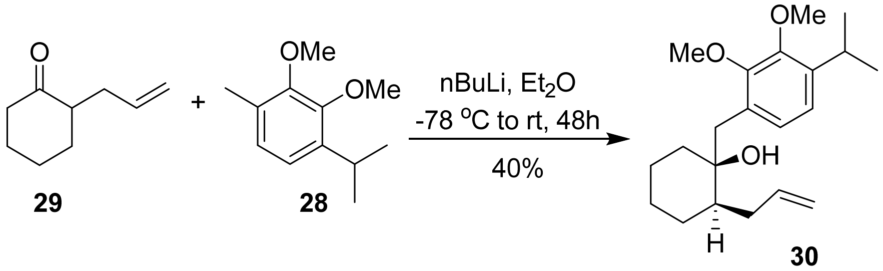

To a solution of aryl moiety **28** (0.38 g, 0.1.96 mmol) and TMEDA (0.48 mL, 2.94 mmol) dissolved in dry Et_2O (3.0 mL) was added 1.2 M (in hexanes) *n*BuLi (2.45 mL, 2.94 mmol, 1.5 equiv) dropwise under N_2 at -78 °C. The reaction mixture was then stirred at rt for 4 hours period and a bright yellow solution was observed. The solution was then re-cooled to -78 ºC followed by dropwise addition of allyl-ketone **29** (0.41 g, 2.94 mmol) in 2 mL dry Et_2O solvent. The reaction was allowed to reach rt and stirred for 48 h. The resulting dark red mixture was then quenched with H_2O. The aqueous layer was then extracted (3 × 10 mL) with EtOAc. The combined organic extracts were dried with anhydrous $MgSO_4$, filtered, and concentrated under reduced pressure. The residue was left in vacuo for overnight to remove the excess of allyl-ketone **29**. Column chromatography: 5% diethyl ether/hexanes. Yield: 40% (0.26 g, 0.78 mmol). Colorless oil. ^{1}H NMR (400 MHz, $CDCl_3$) δ: 6.90 (d, *J*= 8.0 Hz, 1H), 6.83 (*J*= 7.6.0 Hz, 1H), 5.91-5.81 (m, 1H), 5.09-4.99 (m, 2H), 3.86 (s, 3H), 3.83 (s, 3H), 3.29 (quint, *J*= 6.8 Hz, 1H), 3.24 (d, *J*= 13.6 Hz, 1H), 2.99 (bs, 1H), 2.62-2.57 (m, 1H), 2.48 (d, *J*= 13.6 Hz, 1H), 2.09-2.01 (m, 1H), 1.68-1.63 (m, 2H), 1.49-1.35 (m, 5H), 1.23-1.17 (m, 2H), 1.22 (d, *J*= 6.8 Hz, 3H), 1.20 (d, *J*= 6.8 Hz, 3H). ^{13}C NMR (125 MHz, $CDCl_3$) δ: 151.1, 150.5, 141.6, 138.9, 129.2, 127.4, 121.2, 115.5,

73.6, 60.8, 60.2, 45.6, 41.5, 36.9, 34.3, 27.6, 26.9, 25.5, 23.8, 23.6, 22.1. IR ν_{max} (cm^{-1}): 3487, 3079, 2954, 2941, 2879, 1632, 1476, 1418, 1380, 1331, 1286, 1214, 1059, 1027, 914, 843, 806. HRMS (ESI/TOF) m/z: $[M + Na]^+$ Calculated for $C_{21}H_{32}O_3Na$ 355.2244; Found 355.2266.

(3aR,7aS)-7a-(4-isopropyl-2,3-dimethoxybenzyl)octahydrobenzofuran-2-ol (27)

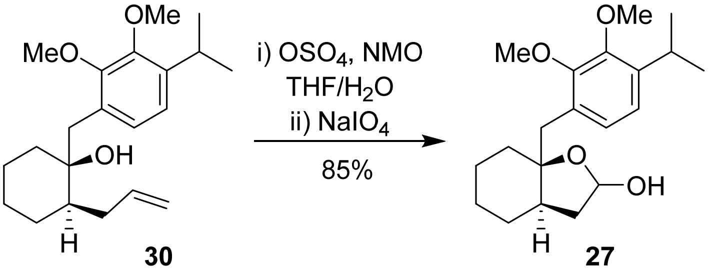

To a solution of alkene **30** (0.033 g, 0.10 mmol) and NMO (0.025 g, 0.21 mmol) in THF/H_2O (3:1, 1.5 mL) under stirring at room temperature was added of 0.15 M OsO_4 (13.0 μL, 2.0 mol%) and allowed to stir for 4 hours. After addition of $NaIO_4$ (0.052 g, 0.24 mmol) the mixture stirred at room temperature for further 2 hours. To this reaction mixture 10% $Na_2S_2O_3$ (10 mL) solution was added and extracted with diethyl ether (3 x 10). The combined organic phase was washed with H_2O (10 mL) and saturated NaCl solution (10 mL), dried over anhydrous $MgSO_4$ and the solvent was evaporated. Column chromatography: 5-8% ethyl acetate/hexanes. Yield: 85% (0.029 g, 0.085 mmol). colourless oil. ^1H NMR (500 MHz, $CDCl_3$) δ: 7.01 (d, *J*= 8.5 Hz, 1H), 6.90 (d, *J*= 8.5 Hz, 1H), 5.43-5.40 (m, 1H), 3.85 (s, 3H), 3.83 (s, 3H), 3.29 (quint, *J*= 7.0 Hz, 1H), 3.03 (d, *J*= 13.5 Hz, 1H), 3.02 (bs, 1H), 2.81 (d, *J*= 13.5 Hz, 1H), 2.16-2.13 (m, 1H), 2.09-2.04 (m, 1H), 1.92-1.88 (m, 1H), 1.77-1.34 (m, 1H), 1.71-1.67 (m, 1H), 1.62-1.69 (m, 1H), 1.52-1.47 (m, 2H), 1.43-1.37 (m, 3H), 1.20 (d, *J*= 7.0 Hz, 3H), 1.197 (d, *J*= 7.0 Hz, 3H). ^{13}C NMR (125 MHz, $CDCl_3$) δ: 151.7, 150.4, 141.3, 129.1, 127.3, 120.8, 98.0, 84.7, 60.8, 60.2, 39.6, 39.2, 38.6, 34.2, 26.8, 26.6, 23.7, 23.6, 22.7, 21.9. IR ν_{max} (cm^{-1}): 3392, 2966, 2943, 2864, 1487, 1457, 1423, 1359, 1342, 1279, 1251, 1218, 1149, 1045, 1024, 961, 847, 814. HRMS (ESI/TOF) m/z: $[M + H]^+$ Calculated for $C_{20}H_{31}O_4$ 335.2217; Found 335.2229.

(4aS,10S,11aR)-8-isopropyl-6,7-dimethoxy-1,2,3,4,5,10,11,11a-octahydro-4a,10-epoxydibenzo[a,d][7]annulene (12)

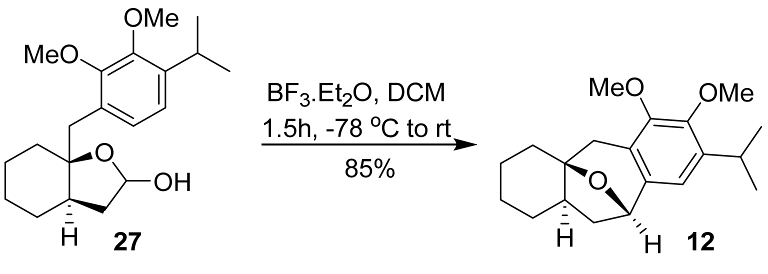

To a solution of acetal **27** (0.10 g, 0.32 mmol) dissolved in DCM (6 mL) was added $BF_3 \bullet OEt_2$ (0.08 mL, 0.63 mmol) dropwise under argon at -78 ºC until complete consumption of starting material was

observed by TLC analysis (2.5 h). The reaction was then quenched with saturated aqueous ammonium chloride. The aqueous layer was then extracted (3 × 3 mL) with EtOAc. The combined organic extracts were dried with anhydrous $MgSO_4$, filtered, and concentrated under reduced pressure. Column chromatography: 5% ethyl acetate/hexanes. Yield: 85% (0.09 g, 0.27 mmol). White solid. ^1H NMR (400 MHz, $CDCl_3$) δ: 6.62 (s, 1H), 4.92 (d, *J*= 7.2 Hz, 1H), 3.82 (s, 3H), 3.80 (s, 3H), 3.27 (quint, *J*= 6.8 Hz, 1H), 2.78 (d, *J*= 17.6 Hz, 1H), 2.56 (d, *J*= 17.6 Hz, 1H), 2.09-1.86 (m, 4H), 1.73-1.56 (m, 6H), 1.26-1.23 (m, 1H), 1.19 (d, *J*= 6.8 Hz, 3H), 1.19 (d, *J*= 6.8 Hz, 1H). ^{13}C NMR (125 MHz, $CDCl_3$) δ: 150.9, 148.9, 140.1, 138.9, 123.9, 116.1, 79.7, 76.1, 60.8, 59.8, 44.1, 42.2, 38.1, 33.5, 30.6, 26.8, 23.9, 23.7, 21.8, 19.8. IR ν_{max} (cm^{-1}): 2959, 2942, 2863, 1627, 1454, 1412, 1339, 1266, 1084, 1057, 1021, 812. HRMS (ESI/TOF) m/z: $[M + Na]^+$ Calculated for $C_{20}H_{28}O_3Na$ 339.1931; Found 339.1967.

(4aS,10S,11aR)-8-isopropyl-1,2,3,4,5,10,11,11a-octahydro-4a,10-epoxydibenzo[a,d][7]annulene-6,7-diol (13)

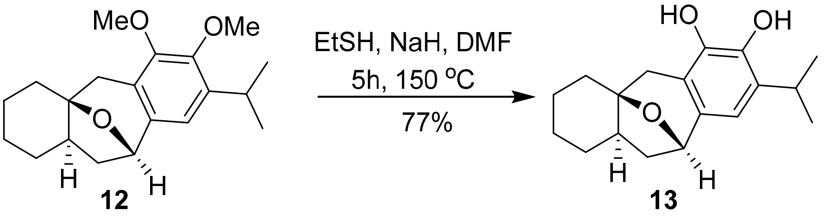

Under inert atmosphere N_2, NaH (0.02 g, 0.5 mmol, 60% in mineral oil) was washed with anhydrous hexanes (3 x 5 mL). After a few minutes, anhydrous DMF (1.5 mL) was added. To this mixture was slowly added a solution of EtSH (0.55 mL, 0.74 mmol) in anhydrous DMF (0.1 mL) at 0 °C and the resulting yellow solution was stirred for 20 min at rt. A solution of compound **12** (0.0125 g, 0.04 mmol) in DMF (1 mL) was then added dropwise and the resulting mixture was stirred for 5 h at 150 °C. When the reaction became slightly brown, the mixture was cooled to the rt and a saturated solution of NH_4Cl (5 mL) was added. The mixture was extracted with Et_2O (3 x 3 mL). The organic phase was washed with H_2O, saturated NaCl solution and dried over anhydrous $MgSO_4$. The solvent was removed under reduced pressure. Column chromatography: 20-40% ethyl acetate/hexanes. Compound **13** yield: 77% (0.009 g, 0.023 mmol). White solid. ^1H NMR (400 MHz, $CDCl_3$) δ: 6.44 (s, 1H), 5.08 (bs, 2H), 4.92 (d, *J*= 7.6 Hz, 1H), 3.10 (d, *J*= 6.8 Hz, 1H), 2.72 (d, *J*= 16.6 Hz, 1H), 2.46 (d, *J*= 16.8 Hz, 1H), 2.07-1.87 (m, 4H), 1.71-1.55 (m, 6H), 1.26-1.20 (m, 1H), 1.23 (d, *J*= 6.8 Hz, 3H), 1.22 (d, *J*= 6.8 Hz, 3H). ^{13}C NMR (100 MHz, $CDCl_3$) δ: 141.6, 139.5, 135.4, 131.9, 116.3, 112.4, 79.6, 76.3, 44.3, 42.4, 37.5, 33.6, 30.7, 27.3, 22.9, 22.8, 21.9, 19.9. IR ν_{max} (cm^{-1}): 3363 (br), 2959, 2865, 1627, 1567, 1449, 1371, 1318, 1273, 1167, 1092, 1012, 978. HRMS (ESI/TOF) m/z: $[M]^{+\cdot}$ Calculated for $C_{18}H_{24}O_3$ 288.1725; Found 288.1736.

(4aS,10S,11aR)-9-bromo-8-isopropyl-6,7-dimethoxy-1,2,3,4,5,10,11,11a-octahydro-4a,10-epoxydibenzo[a,d][7]annulene (14)

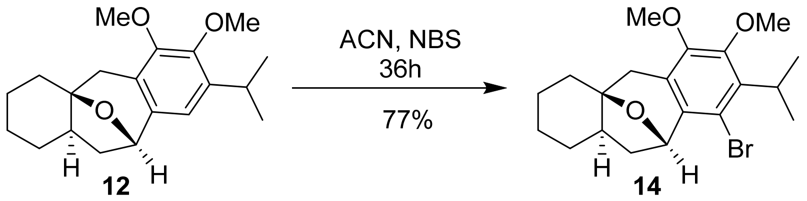

N-bromosuccinimide (NBS) (0.032 g, 0.18 mmol) was added to an MeCN solution (1.5 mL) of icetaxane **12** (0.044 g, 0.14 mmol) and the resulting mixture was stirred at room temperature for 36 h. After evaporation of MeCN under reduced pressure, the residue was dissolved in CH_2Cl_2 and treated with a saturated solution of $NaHCO_3$. The mixture was extracted with CH_2Cl_2 (3 x 5 mL). The organic phase was washed with H_2O and dried over anhydrous $MgSO_4$. The solvent was removed under reduced pressure. Column chromatography: 2-4% ethyl acetate/hexanes. Compound **14** yield: 77% (0.043 g, 0.011 mmol). White solid. ^{1}H NMR (500 MHz, $CDCl_3$) δ: 5.41 (d, *J*= 6.5 Hz, 1H), 3.83 (s, 3H), 3.78 (s, 3H), 3.68-3.58 (m, 1H), 2.78 (d, *J*= 17.5 Hz, 1H), 2.52 (d, *J*= 17.5 Hz, 1H), 2.11-2.16 (m, 1H), 2.00-1.89 (m, 3H), 1.74-1.63 (m, 4H), 1.59-1.53 (m, 1H), 1.33 (d, *J*= 7.0 Hz, 3H), 1.32 (d, *J*= 7.0 Hz, 3H), 1.27-1.19 (m, 2H). ^{13}C NMR (125 MHz, $CDCl_3$) δ: 151.4, 150.9, 138.2, 137.8, 126.7, 116.1, 79.9, 76.4, 60.6, 59.5, 42.7, 41.8, 37.7, 32.9, 29.9, 23.6, 21.4, 21.2, 21.1, 19.3. IR ν_{max} (cm^{-1}): 2973, 2935, 2867, 1611, 1448, 1415, 1344, 1269, 1067, 1051, 1011, 815, 654. HRMS (ESI/TOF) m/z: $[M + H]^+$ Calculated for $C_{20}H_{28}BrO_3$ 395.1216; Found 395.1188.

(4aS,10S,11aR)-8-isopropyl-6,7-dimethoxy-9-phenyl-1,2,3,4,5,10,11,11a-octahydro-4a,10-epoxydibenzo[a,d][7]annulene (15)

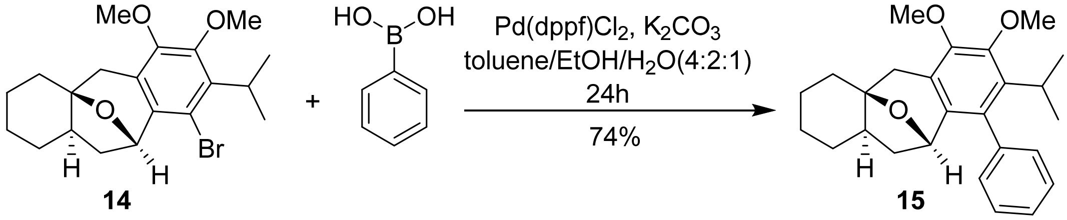

To a stir solution of Bromo-icetaxane **14** (0.002 g, 0.051 mmol) and phenyl boronic acid (0.0075 g, 0.061 mmol) in toluene/ethanol/water (4:2:1) (1.5 mL) was added K_2CO_3 (0.017 g, 0.12 mmol). The solution was stirred and degassed with argon three times, and catalyst (dppf)$PdCl_2$ (0.0022 g, 5% mol.) was added. The dark solution was warmed to reflux for 16 h with the total consumption of starting material. Then the solution was cooled to room temperature, water (2 mL) was added, and the solution was extracted with EtOAc (3 x 2 mL). The organic phase was washed with H_2O, brine and dried over anhydrous $MgSO_4$. The solvent was removed under reduced pressure. Column chromatography: 2-4% ethyl acetate/hexanes. Compound **15** yield: 74% (0.014 g, 0.037 mmol). White solid. ^{1}H NMR (500 MHz, $CDCl_3$) δ: 7.39-7.30 (m, 3H), 7.20-7.18 (m, 1H), 7.02-7.00 (m, 1H), 4.50 (dd, *J*= 7.5, 1.0 Hz, 1H), 3.88 (s, 3H), 3.84 (s, 3H), 2.86 (d *J*= 18.0 Hz, 1H), 2.67 (quint, *J*= 7.0 Hz, 1H), 2.56 (d, *J*= 18.0 Hz, 1H), 2.05-1.90 (m, 4H), 1.68-1.60 (m, 4H), 1.55-1.50 (m, 2H), 1.16 (d, *J*= 7.0 Hz, 3H), 1.15 (d,

J= 7.0 Hz, 3H), 1.12-1.07 (m, 1H). ^{13}C NMR (125 MHz, $CDCl_3$) δ: 150.8, 150.7, 139.9, 137.3, 137.1, 132.5, 130.1, 129.6, 128.4, 128.1, 126.9, 124.4, 79.4, 73.6, 60.2, 59.4, 43.7, 42.7, 38.1, 33.9, 31.2, 30.4, 22.5, 22.1, 22.0, 20.3. IR ν_{max} (cm^{-1}): 2964, 2928, 2866, 1621, 1444, 1409, 1334, 1259, 1071, 1061, 1016, 815. HRMS (ESI/TOF) m/z: $[M + H]^+$ Calculated for $C_{26}H_{33}O_3$ 393.2424; Found 393.2429.

2. Hemozoin Formation Inhibition Assay

The hemozoin formation inhibition assay was performed as described previously.12</sup> Briefly, serial 1:3 dilutions, in a 96-well plate, with brussonol were made in a final volume of 20 μL in a concentration 10 times higher than the final concentration of the assay. Secondly, 90 μL of freshly prepared hemin (at a final concentration of 25 μM) solution in 1M NaOAc/HOAc buffer pH 4.8 were added followed by addition of 90 μL of 5.4 mg/L Tween 20 solution freshly prepared. The plates were let to stand for 4 hours at 37 ºC followed by resuspension with multichannel pippete and read at 415 and 630 nm using a SpectraMAX Plus 384 plate reader (Molecular Devices Corp., Sunnyvale, CA). Due to the absence of enough compound, this experiment was performed only once, but with internal triplicate. Chloroquine (CQ) and pyrimethamine (PYR) were used as positive and negative control of inhibition, respectively.

$$\text{Inhibition (\%)} = 100 - 100 \times [(A_{control} - A_{sample}) / (A_{control} - A_{min})]$$

$A_{control}$ is the difference of the absorbance at 415 and 630 nm of the negative control (Hemin +, Tween 20 -, Compound -); A_{min} is the difference of the absorbance at 415 and 630 nm of the positive control (Hemin +, Tween 20 +, Compound -); A_{sample} is the difference of the absorbance at 415 and 630 nm of the sample. The hemozoin formation inhibition assay was performed to evaluate if brussonol act inhibiting hemozoin formation in *P. falciparum.* CQ, which is a well-known inhibitor of hemozoin formation, showed an IC_{50} = 80 μM. By contrast, PYR, an antimalarial not known to inhibit hemozoin formation, as expected, showed no inhibition profile up to 1.4 mM. Brussonol showed a similar behavior of PYR, leading to conclude that brussonol do not act inhibiting hemozoin formation (Figure S1).

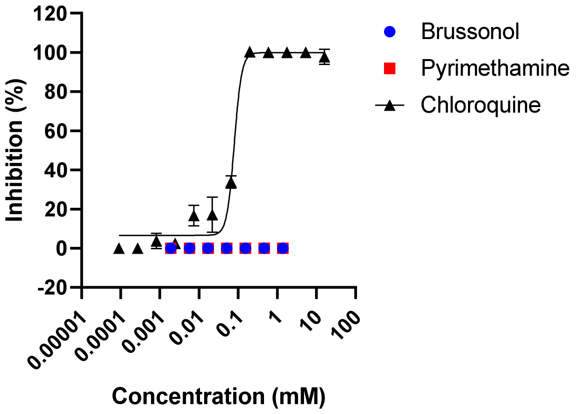

Figure S1. Hemozoin formation inhibition assay. Different concentrations of CQ (black triangles), PYR (red squares) and brussonol (blue circles) were incubated with heme in triplicates. The absorbance was recorded at 415/630 nm to determine the hemozoin formation inhibition. These data correspond to one experiment due to lack of material.

3. References

1. Williams, D. B. G. & Lawton, M. Drying of organic solvents: quantitative evaluation of the efficiency of several desiccants. *J. Org. Chem.* **75**, 8351–4 (2010).
2. Carita, A. & Burtoloso, A. C. B. An epoxide ring-opening approach for a short and stereoselective synthesis of icetexane diterpenoids. *Tetrahedron Letters* vol. 51 686–688 (2010).
3. Padwa, A., Chughtai, M. J., Boonsombat, J. & Rashatasakhon, P. A Rh(II)-catalyzed cycloaddition approach toward the synthesis of komaroviquinone. *Tetrahedron* **64**, 4758–4767 (2008).
4. Martinez-Solorio, D. & Jennings, M. P. Convergent formal syntheses of (±)-brussonol and (±)-abrotanone via an intramolecular Marson-type cyclization. *Org. Lett.* **11**, 189–192 (2009).
5. Stevens, R. V. & Bisacchi, G. S. Benzocyclobutenones as Synthons for the Synthesis of C-11 Oxygenated Diterpenoids. Application to the Total Synthesis of (±)-Taxodione. *J. Org. Chem.* **47**, 2396–2399 (1982).
6. Simmons, E. M. & Sarpong, R. Structure, biosynthetic relationships and chemical synthesis of the icetexane diterpenoids. *Nat. Prod. Rep.* **26**, 1195–1217 (2009).
7. Jiao, Z. W. *et al.* Tandem C-H oxidation/cyclization/rearrangement and its application to asymmetric syntheses of (-)-brussonol and (-)-przewalskine E. *Nat. Commun.* **6**, 1–7 (2015).
8. Della Ca', N., Sassi, G. & Catellani, M. A direct palladium-catalyzed route to selectively substituted carbazoles through sequential C-C and C-N bond formation: Synthesis of carbazomycin A. *Adv. Synth. Catal.* **350**, 2179–2182 (2008).
9. Zheng, Y., Liu, Y. & Wang, Q. Collective asymmetric synthesis of (-)-Antofine, (-)-cryptopleurine, (-)-tylophorine, and (-)-tylocrebrine with tert- butanesulfinamide as a chiral auxiliary. *J. Org. Chem.* **79**, 3348–3357 (2014).
10. Majetich, G. *et al.* Synthesis of (±)- and (+)-perovskone. *Tetrahedron* **67**, 10129–10146 (2011).
11. Fernandes, R. A., Mulay, S. V. & Chavan, V. P. A concise total synthesis of arizonins B1 and C1. *Tetrahedron Asymmetry* **24**, 1548–1555 (2013).
12. Huy, N. T. *et al.* Simple colorimetric inhibition assay of heme crystallization for high-throughput screening of antimalarial compounds. *Antimicrob. Agents Chemother.* **51**, 350–353 (2007).

4. NMR Spectra

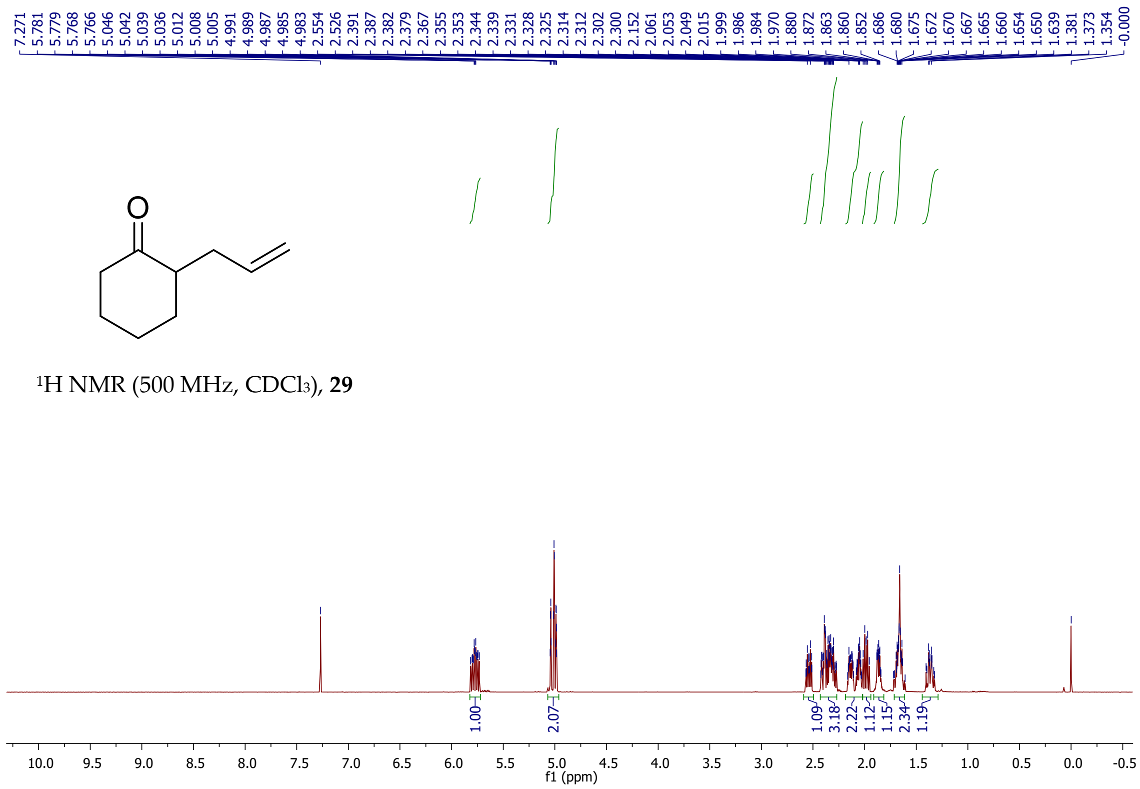

^{1}H NMR (500 MHz, $CDCl_3$), **29**

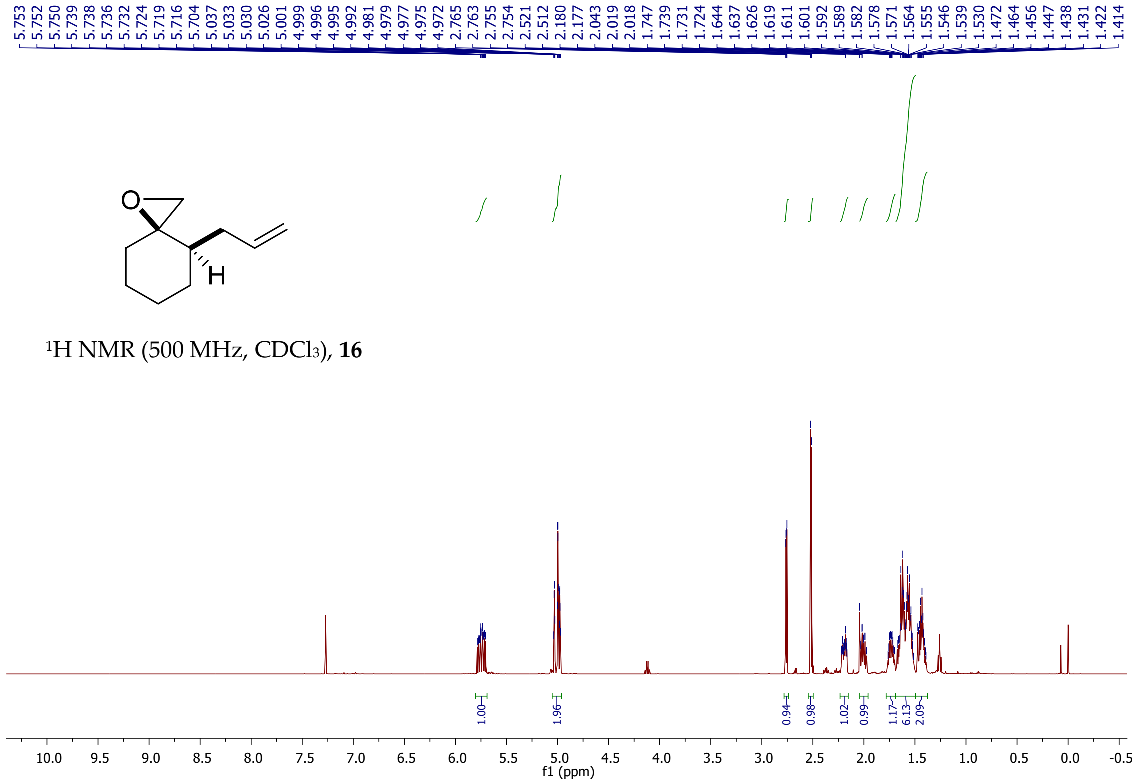

^{1}H NMR (500 MHz, $CDCl_3$), **16**

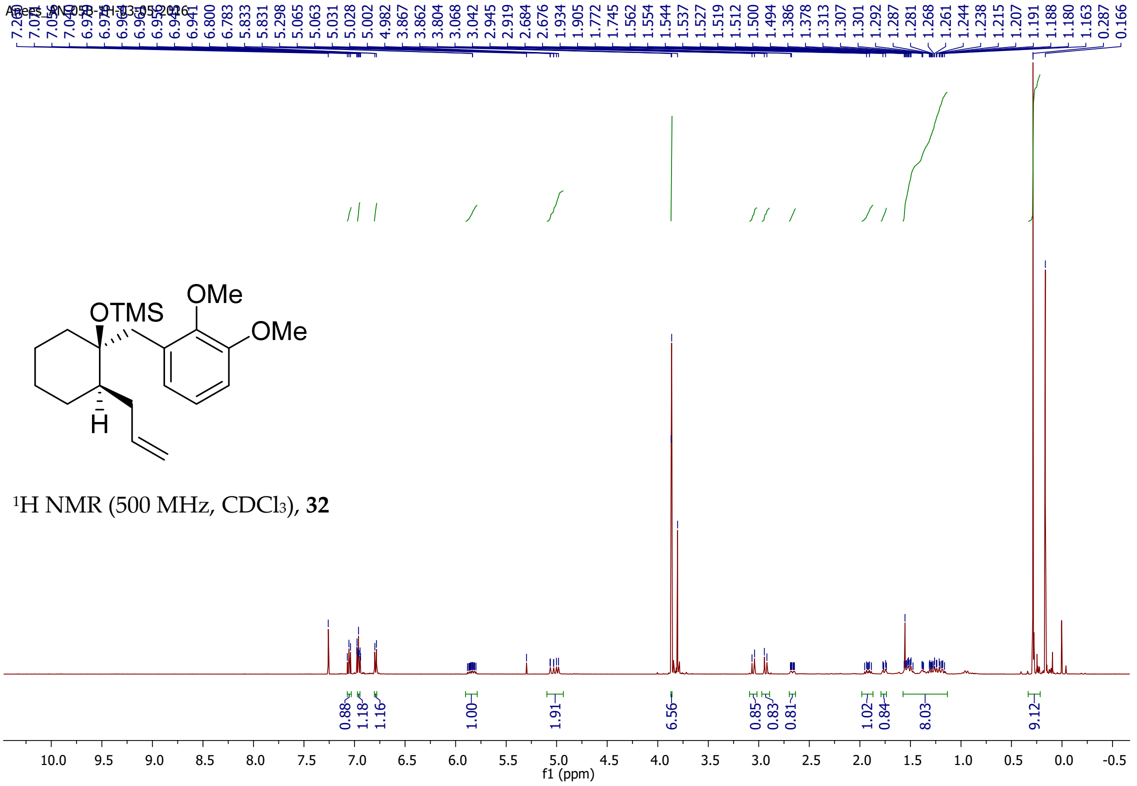

^{1}H NMR (500 MHz, $CDCl_3$), **32**

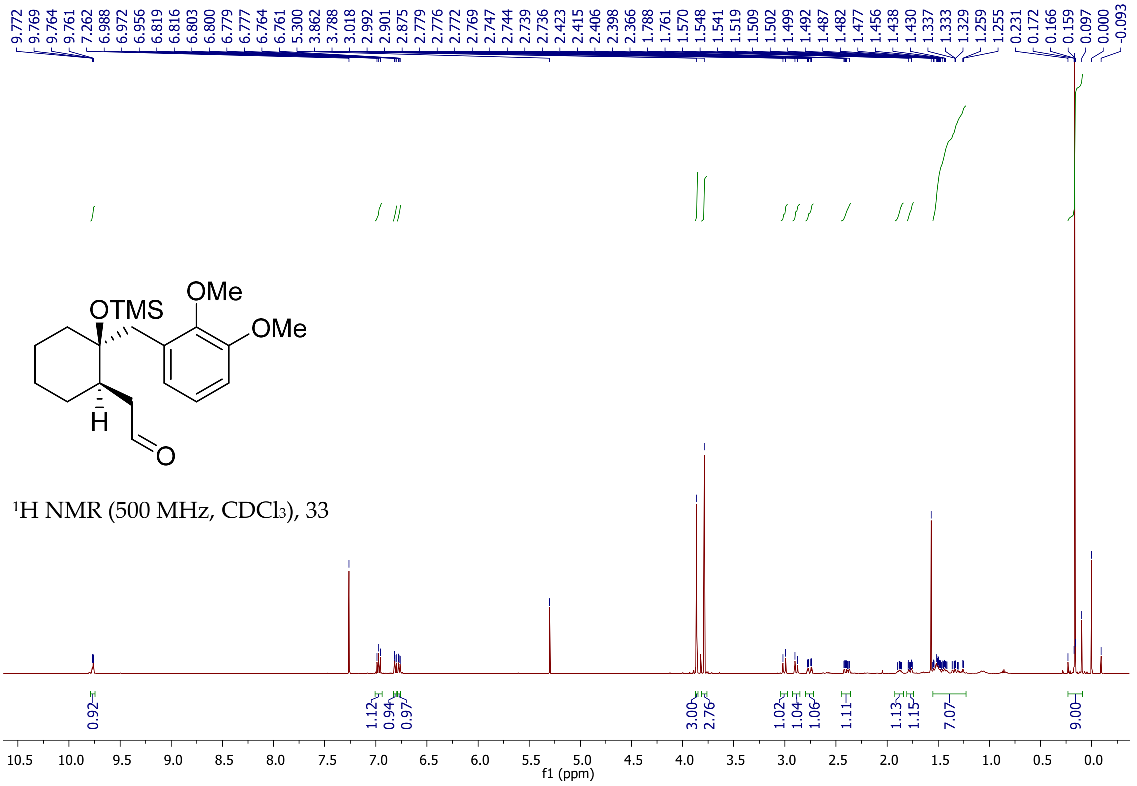

^{1}H NMR (500 MHz, $CDCl_3$), 33

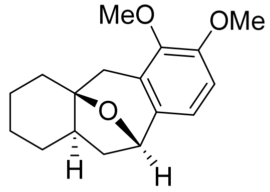

^{1}H NMR (500 MHz, CDCl$_3$), **2**

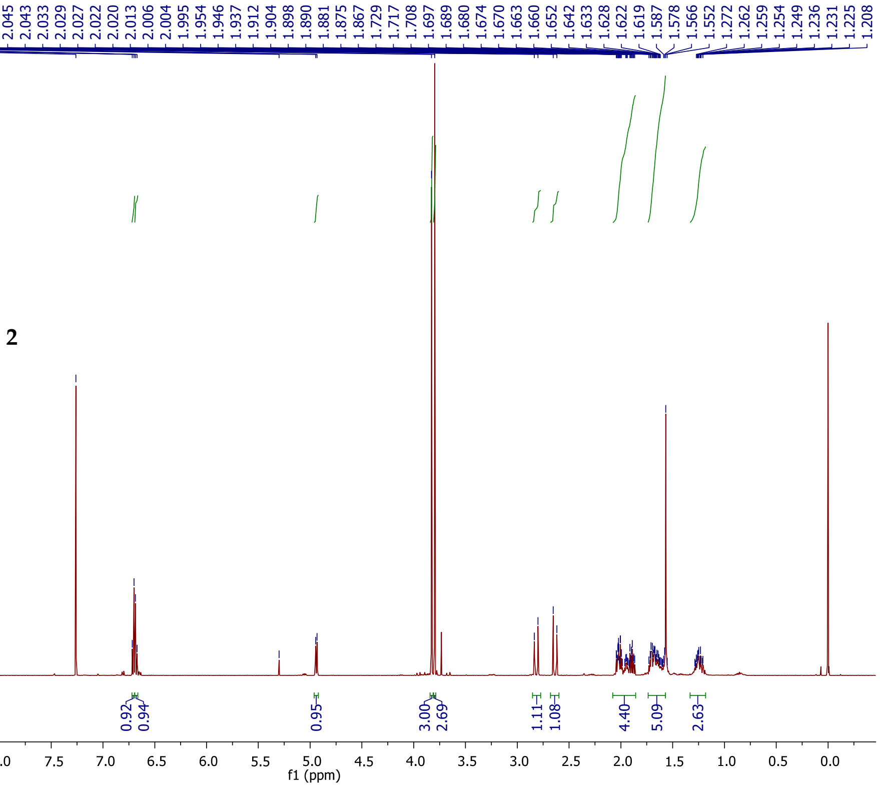

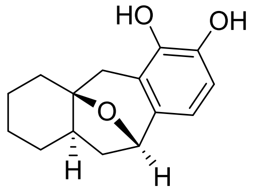

^{1}H NMR (500 MHz, CDCl$_3$), **4**

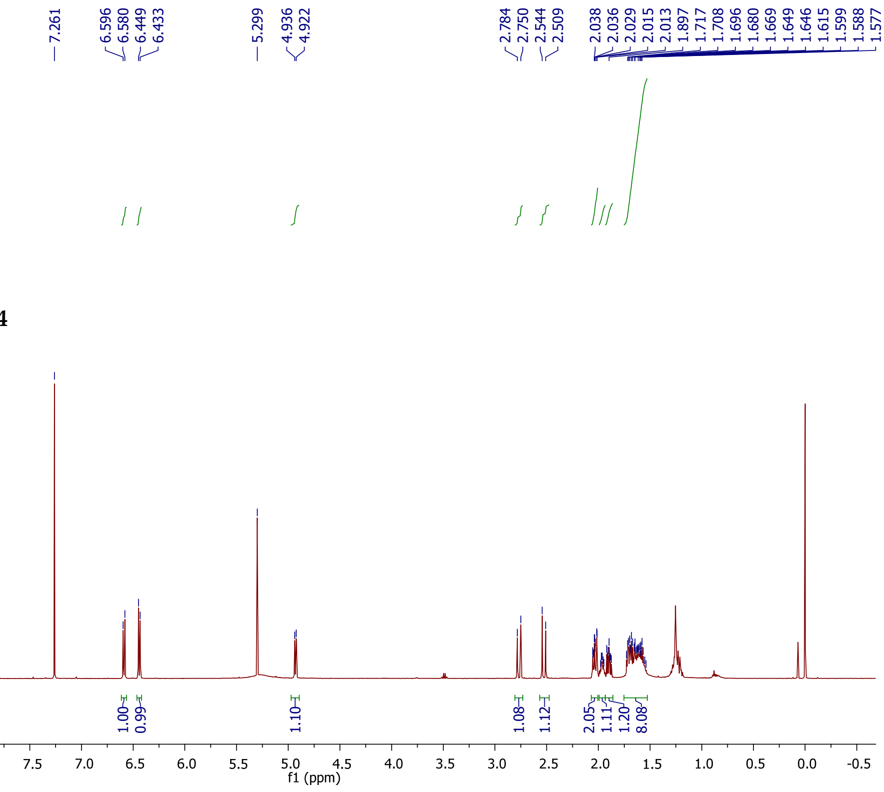

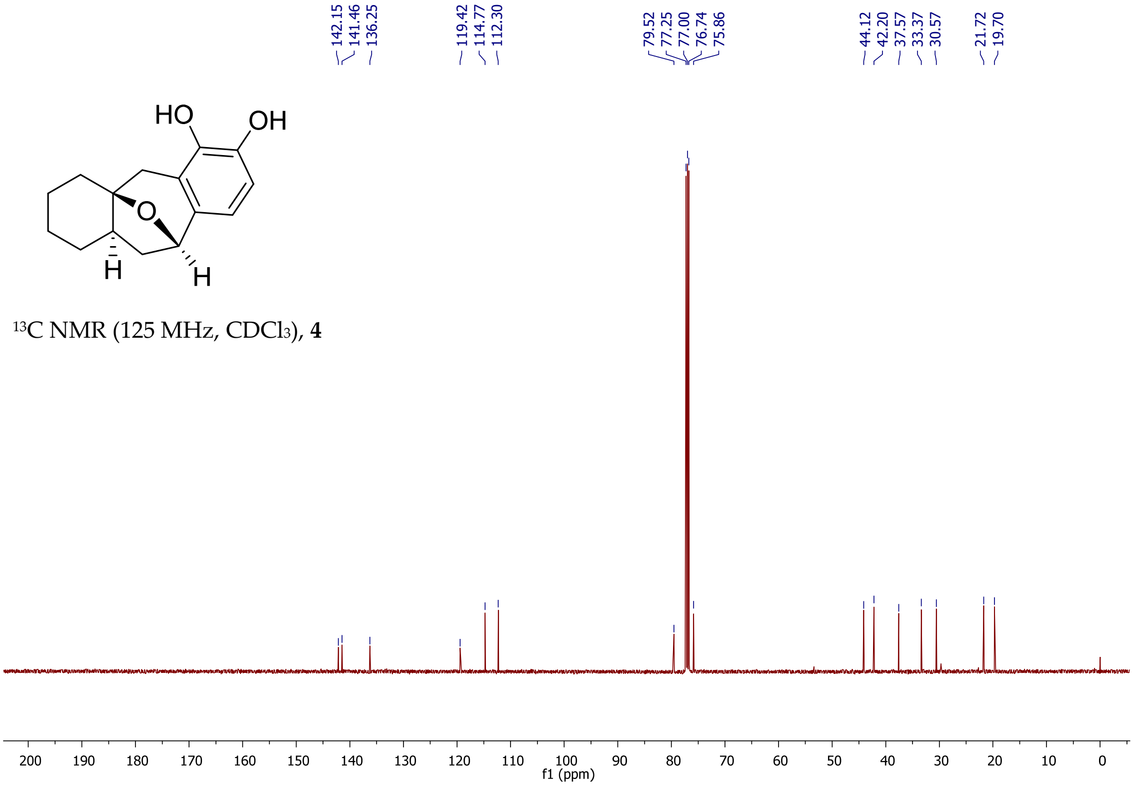

^{13}C NMR (125 MHz, $CDCl_3$), **4**

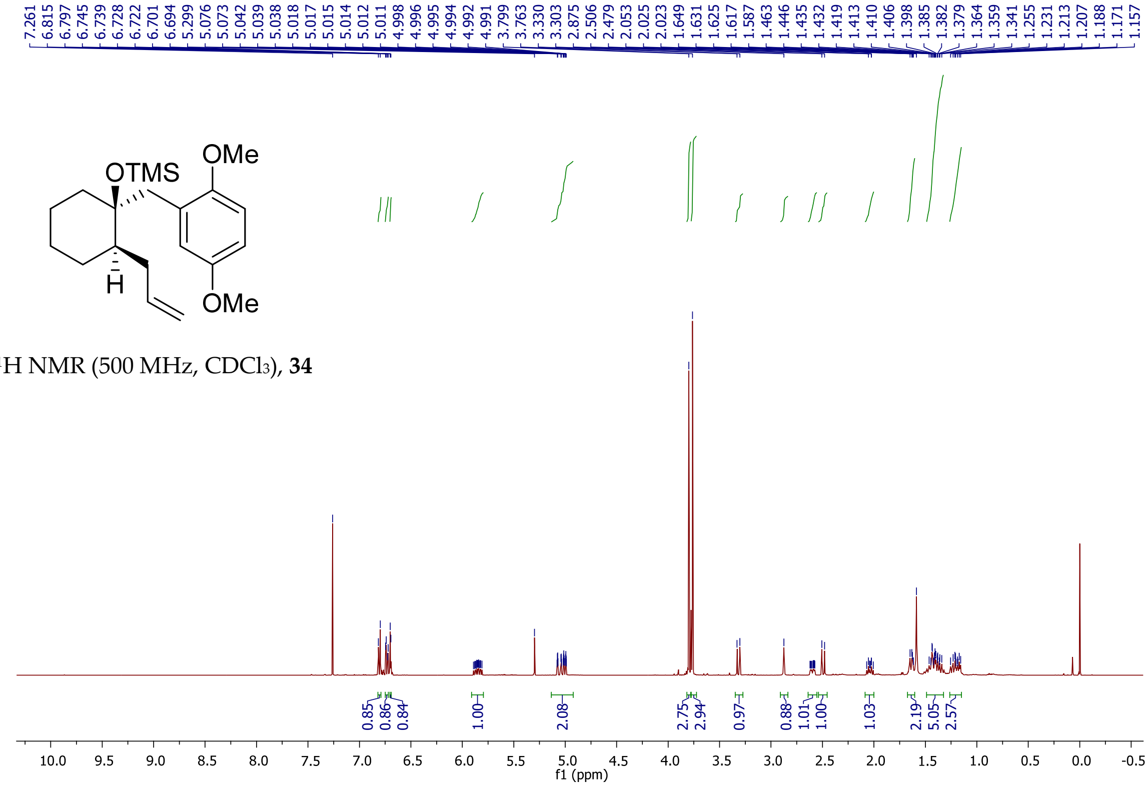

^{1}H NMR (500 MHz, $CDCl_3$), **34**

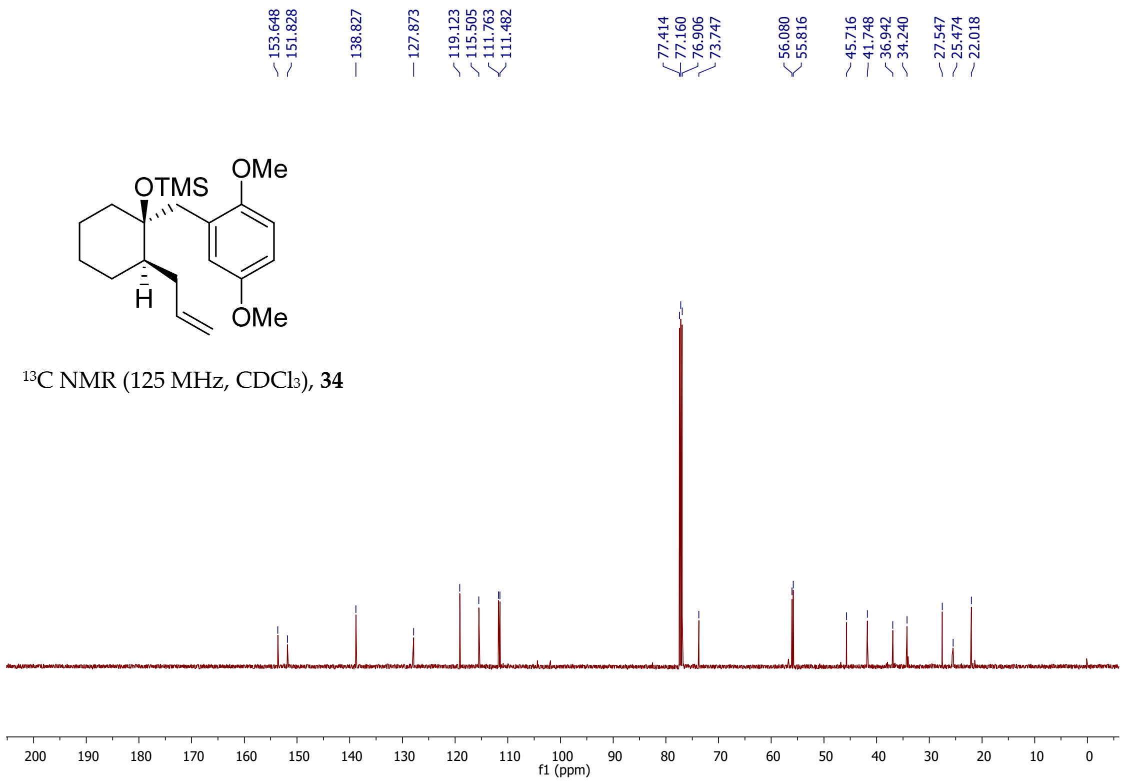

^{13}C NMR (125 MHz, $CDCl_3$), **34**

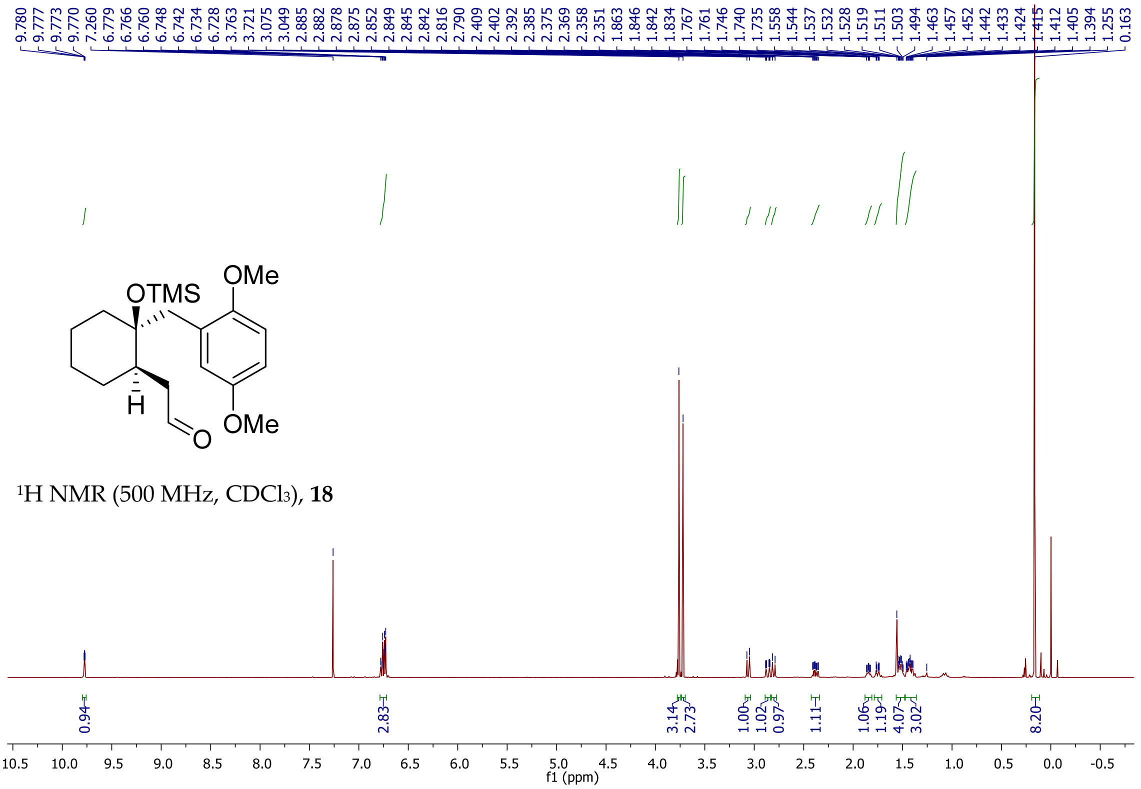

^{1}H NMR (500 MHz, $CDCl_3$), **18**

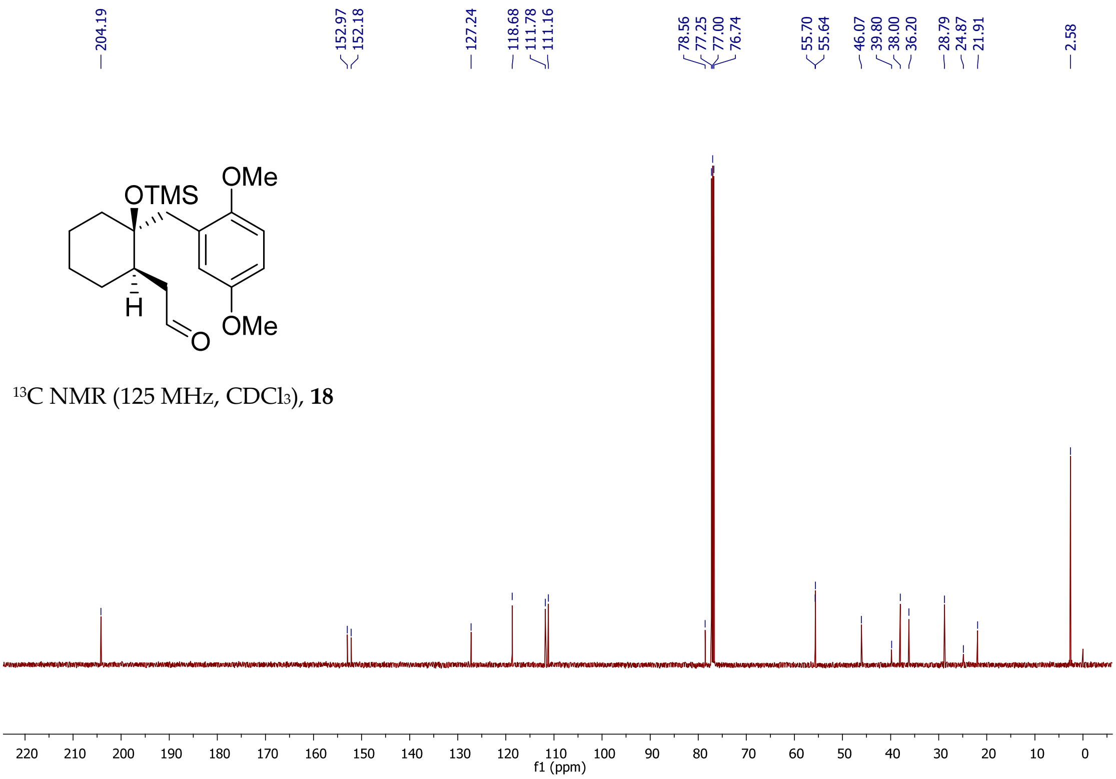

^{13}C NMR (125 MHz, CDCl$_3$), **18**

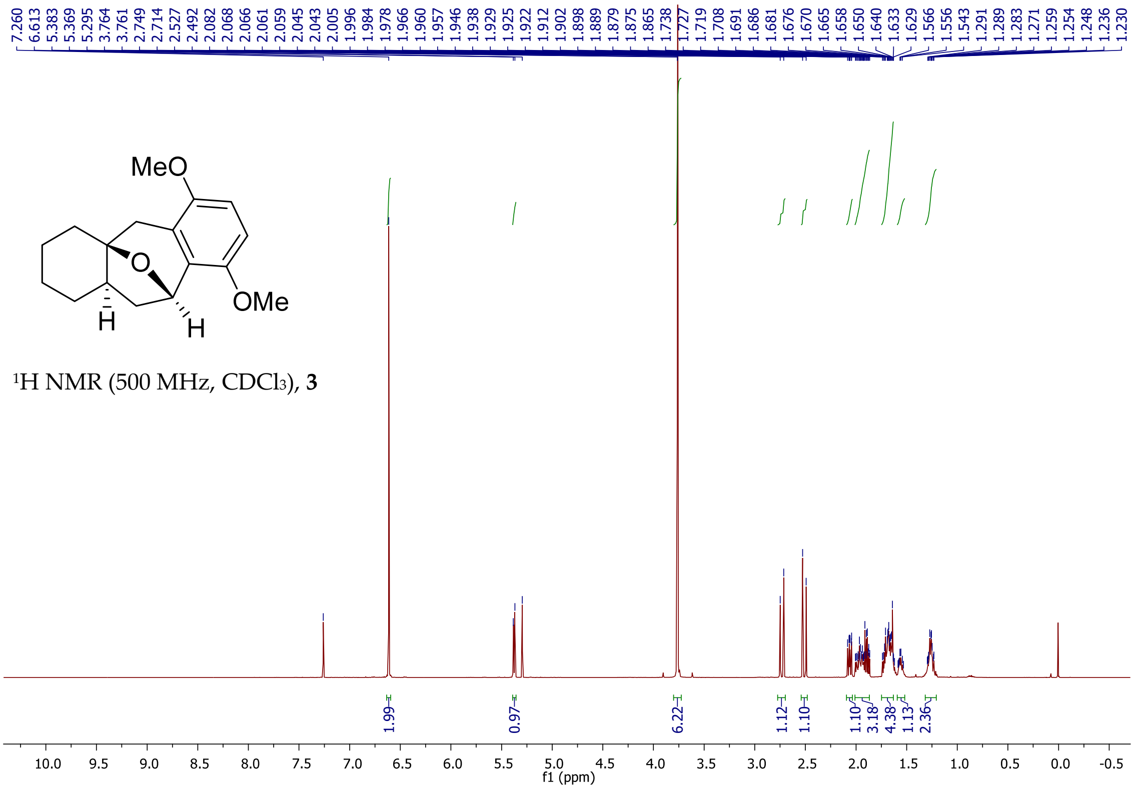

^{1}H NMR (500 MHz, CDCl$_3$), **3**

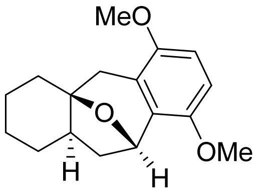

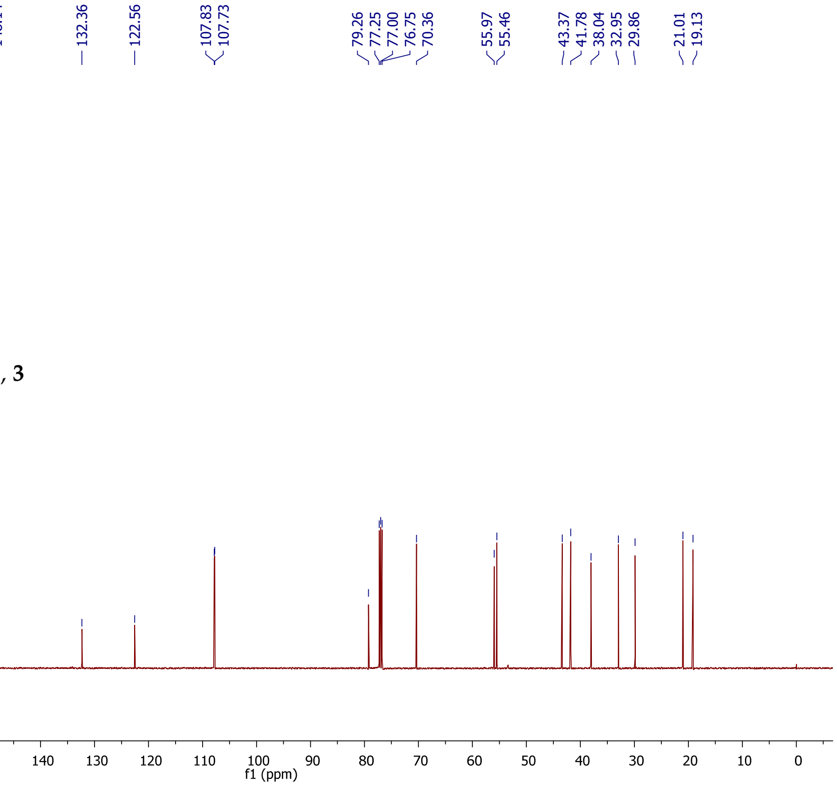

^{13}C NMR (125 MHz, $CDCl_3$), **3**

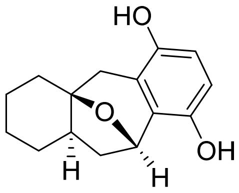

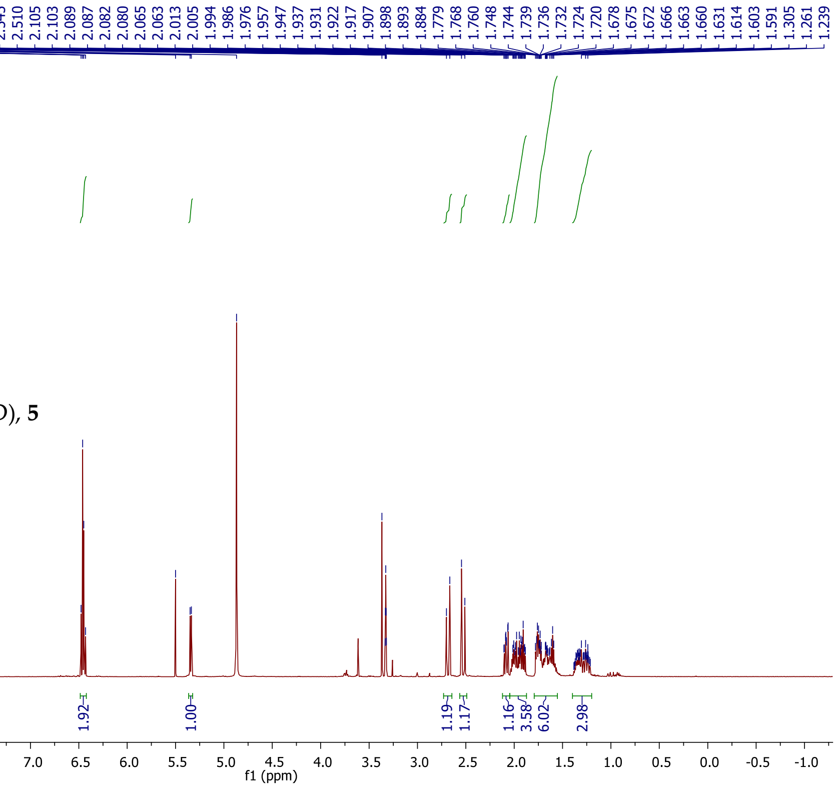

^{1}H NMR (500 MHz, CD_3 OD), **5**

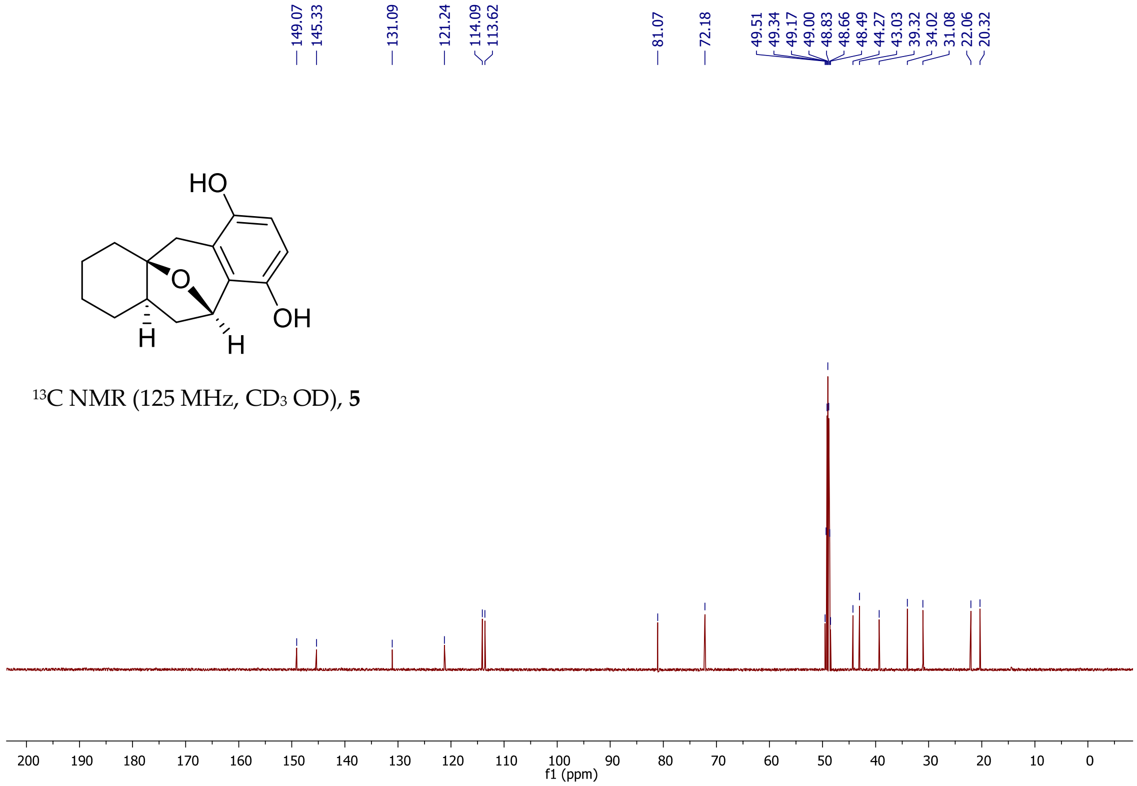

^{13}C NMR (125 MHz, CD_3 OD), **5**

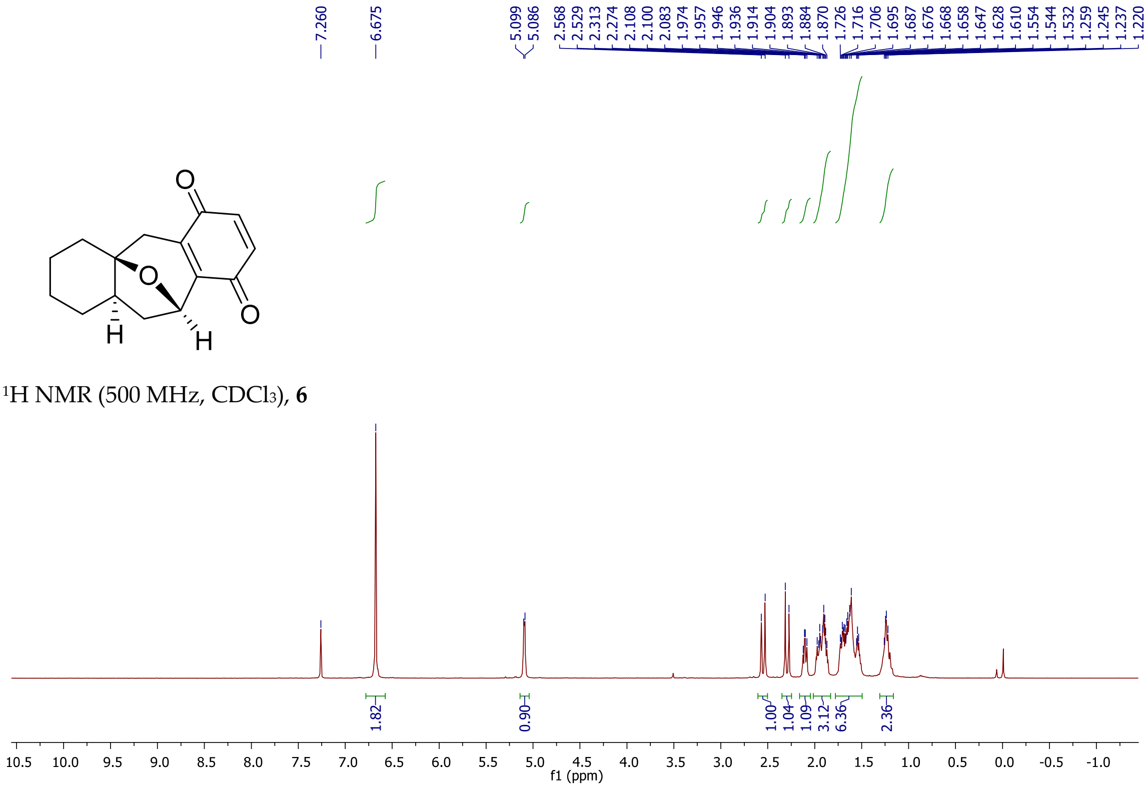

^{1}H NMR (500 MHz, $CDCl_3$), **6**

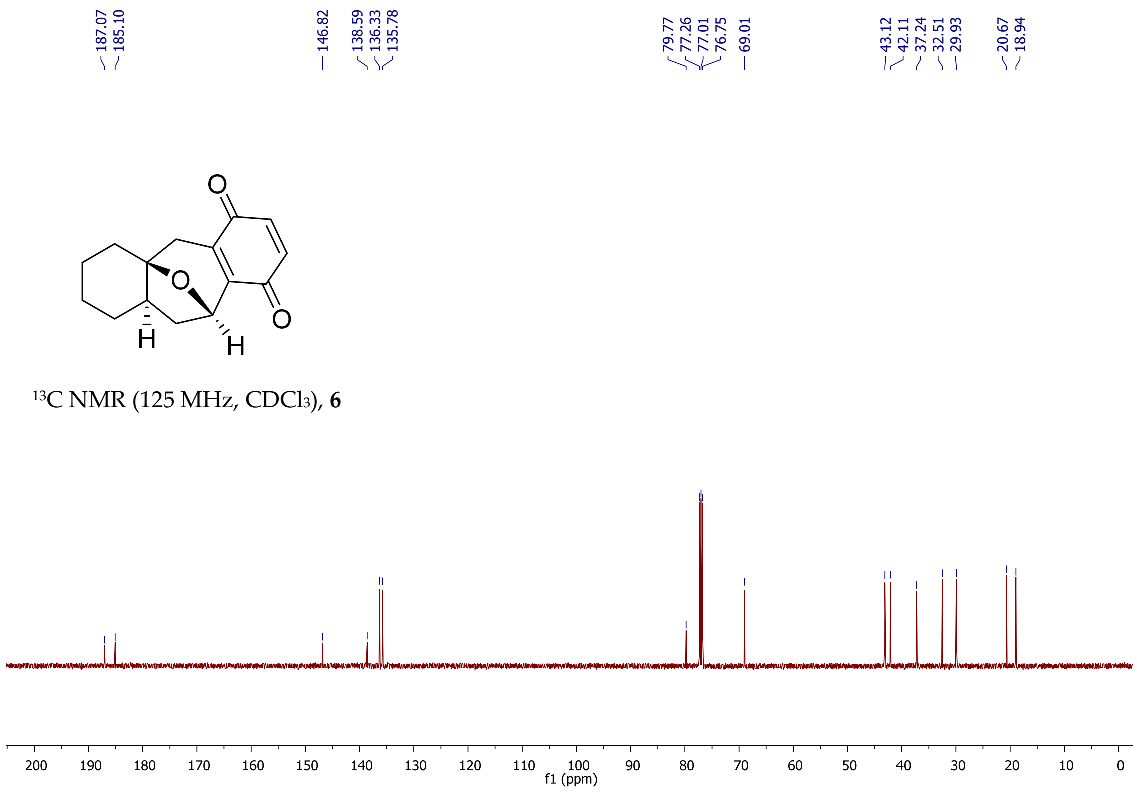

^{13}C NMR (125 MHz, $CDCl_3$), **6**

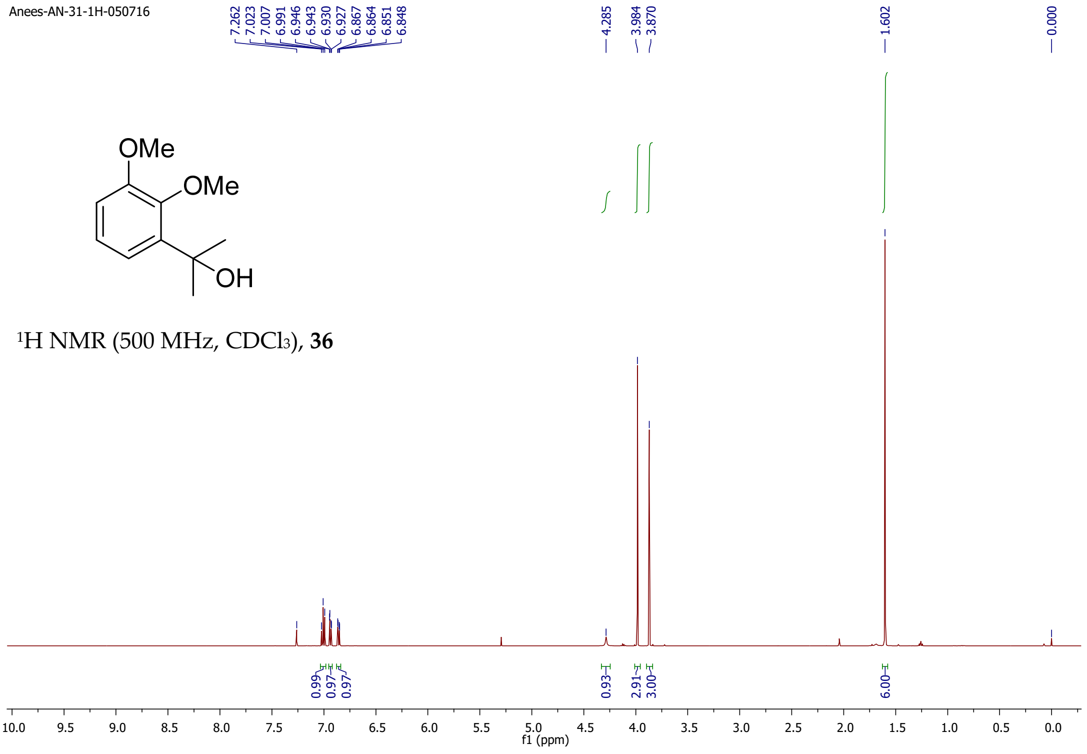

^{1}H NMR (500 MHz, $CDCl_3$), **36**

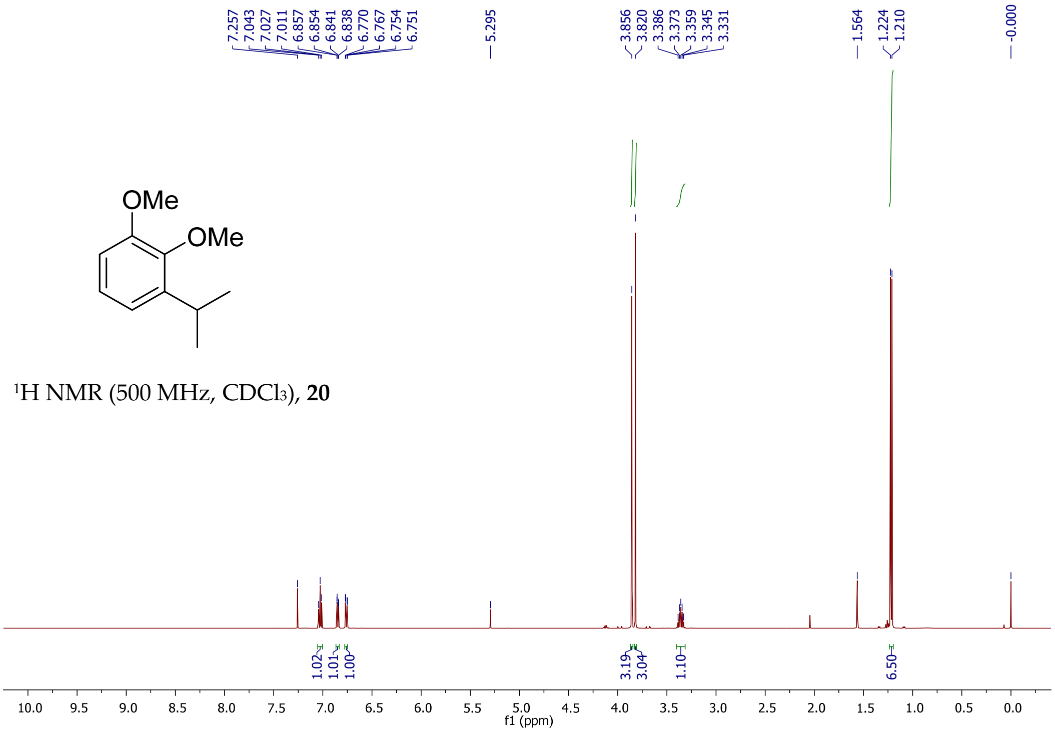

^{1}H NMR (500 MHz, $CDCl_3$), **20**

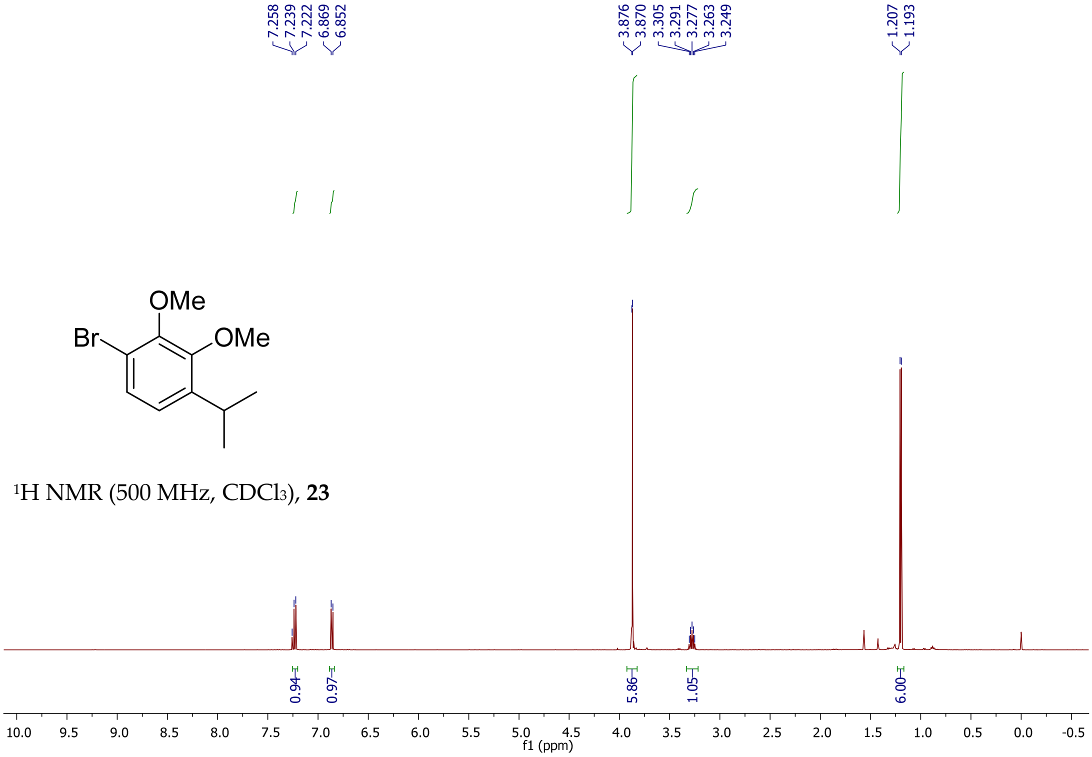

^{1}H NMR (500 MHz, $CDCl_3$), **23**

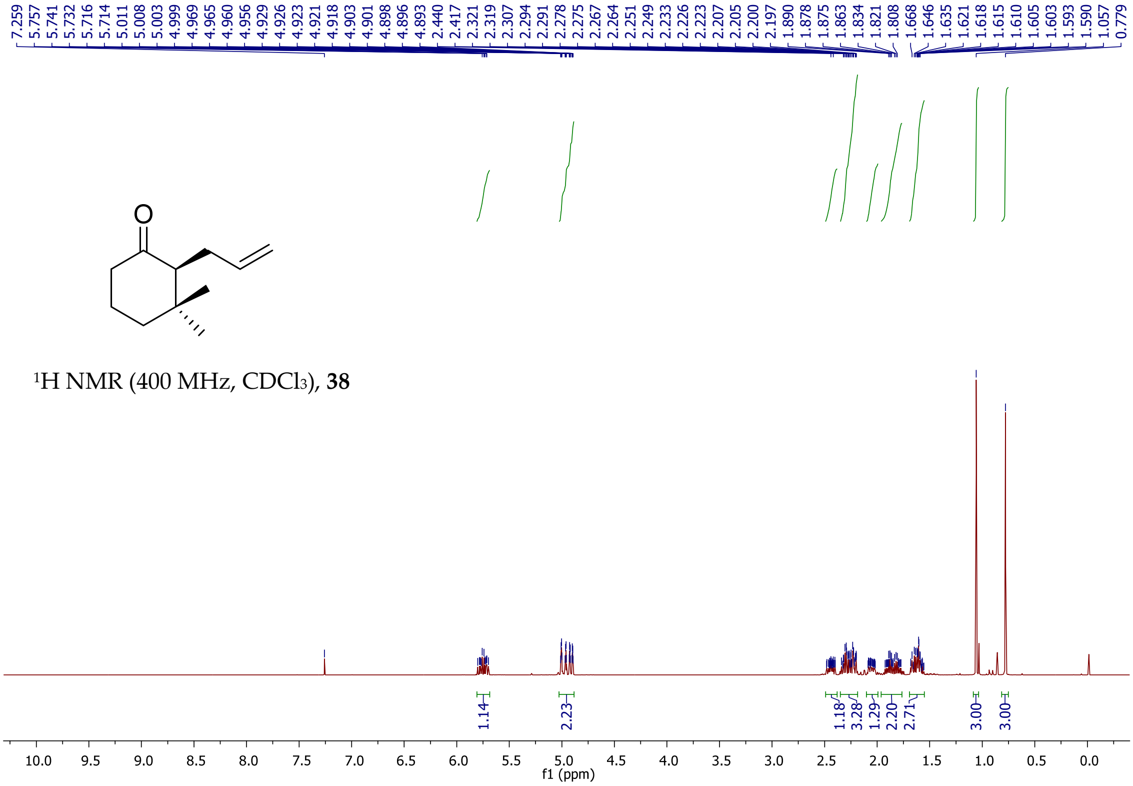

^{1}H NMR (400 MHz, $CDCl_3$), **38**

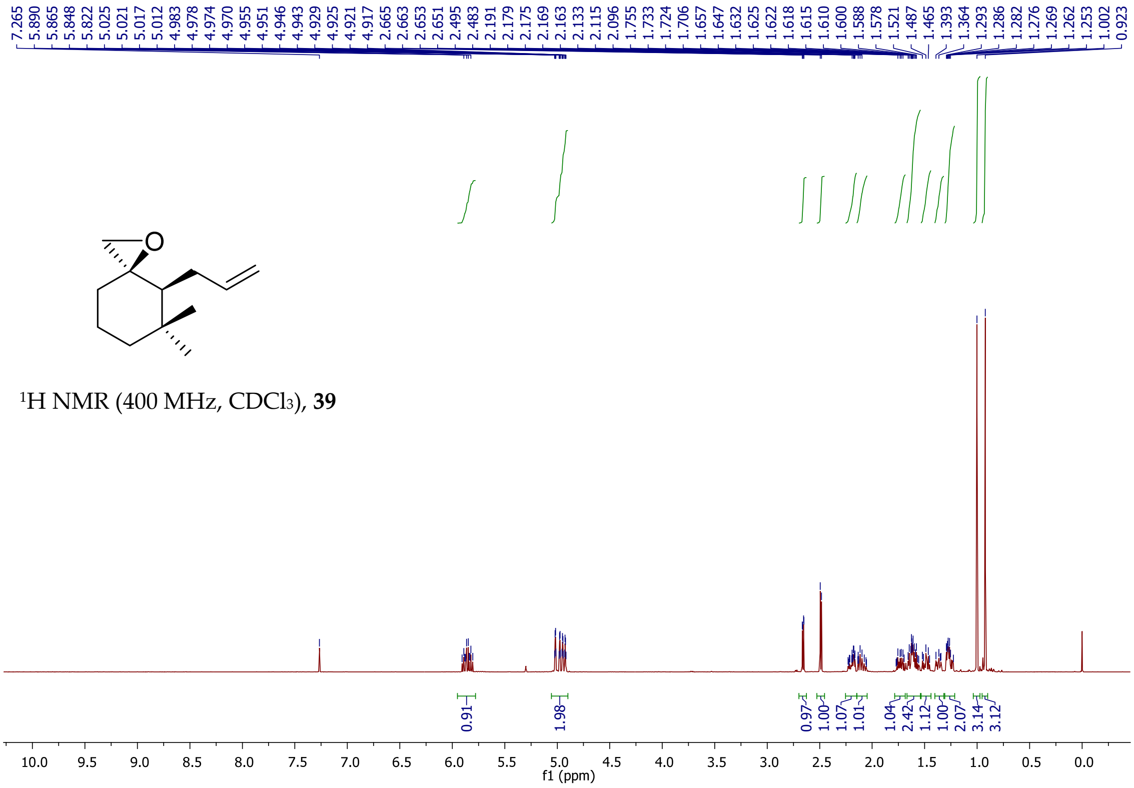

^{1}H NMR (400 MHz, $CDCl_3$), **39**

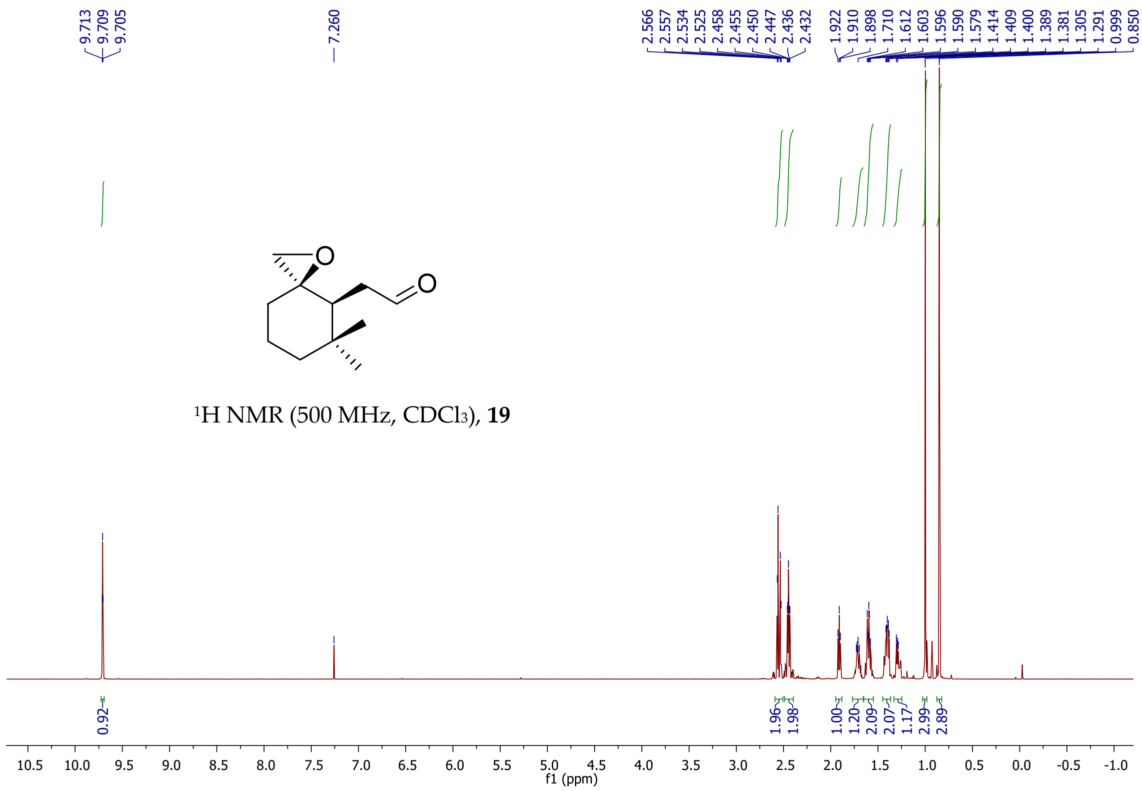

^{1}H NMR (500 MHz, $CDCl_3$), **19**

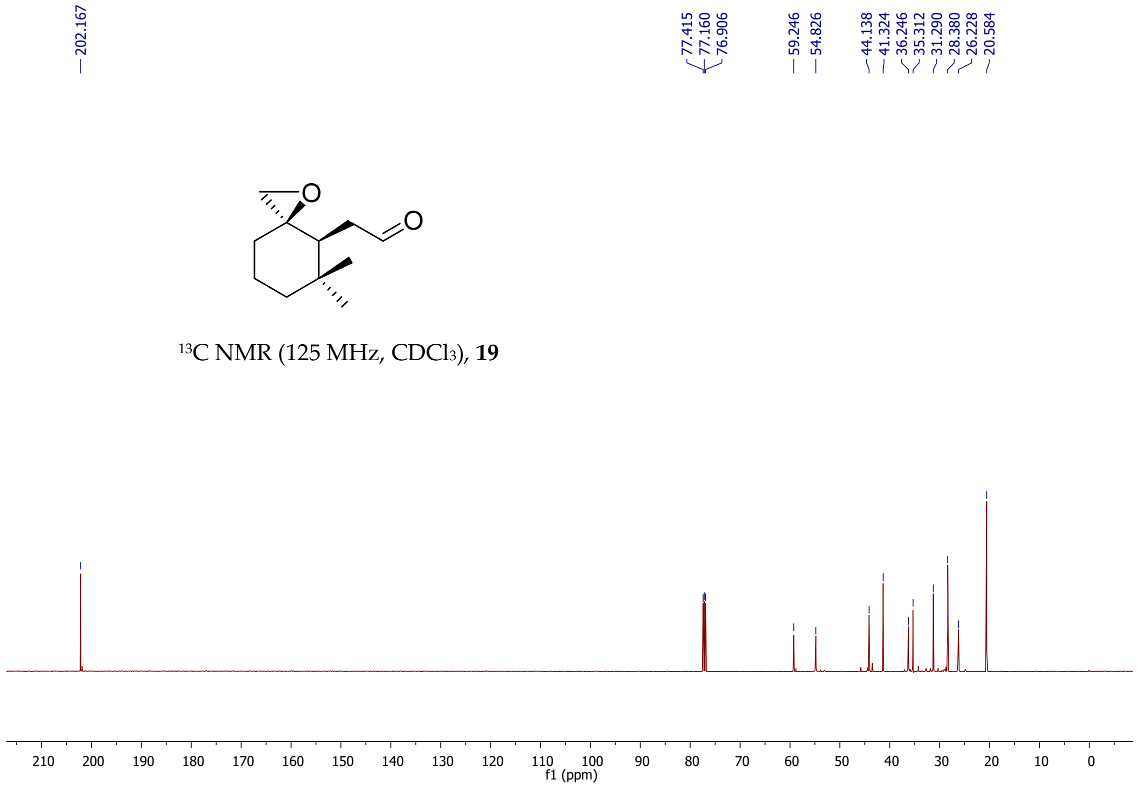

^{13}C NMR (125 MHz, $CDCl_3$), **19**

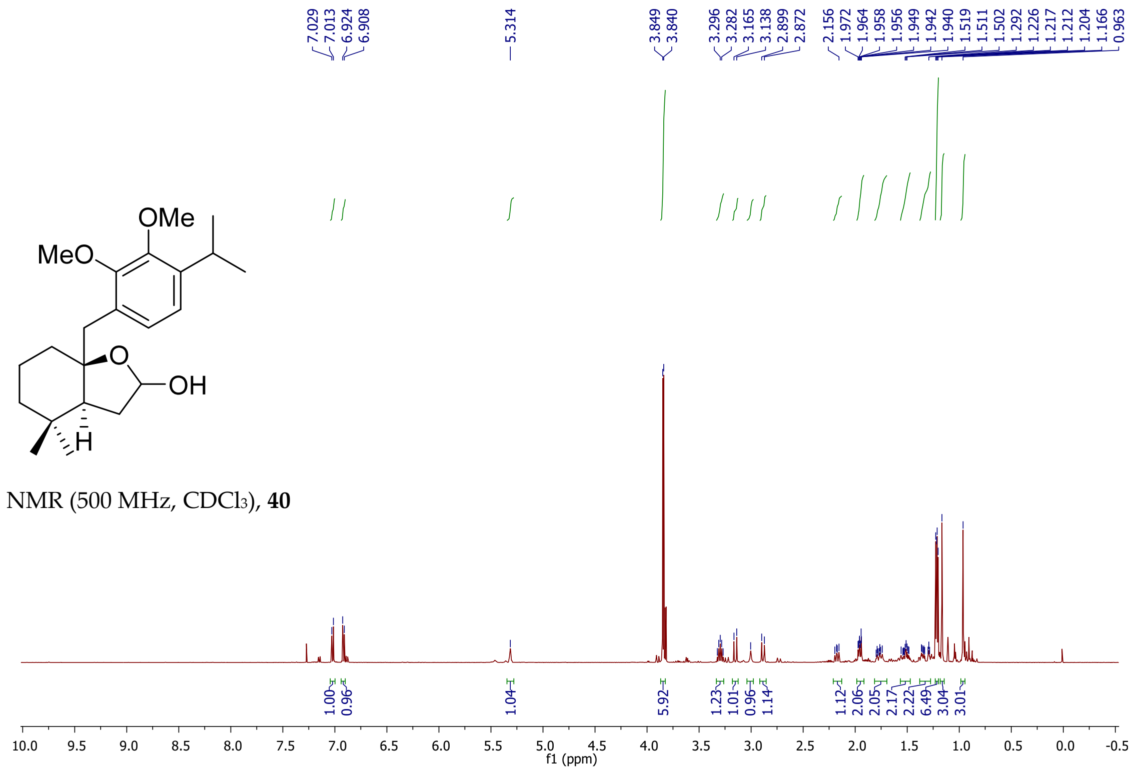

^{1}H NMR (500 MHz, $CDCl_3$), **40**

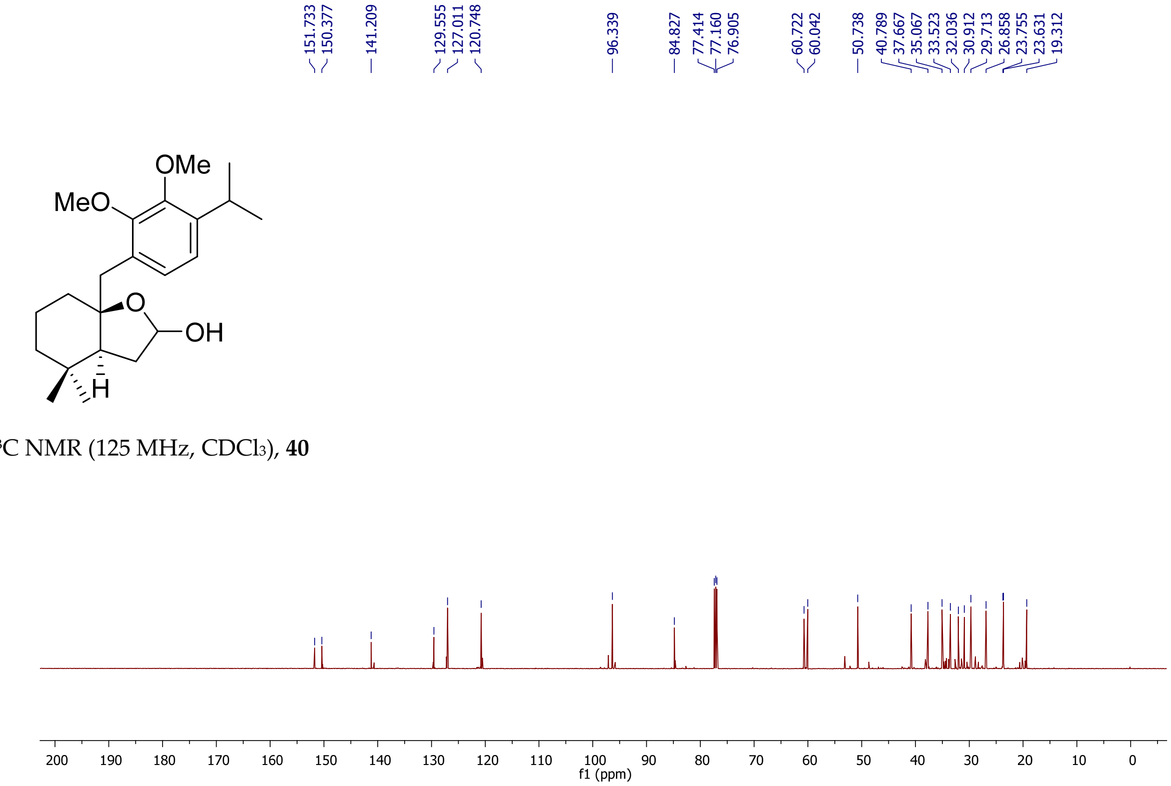

^{13}C NMR (125 MHz, $CDCl_3$), **40**

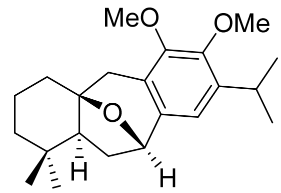

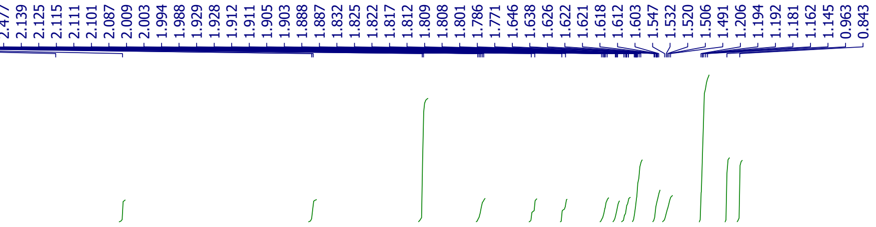

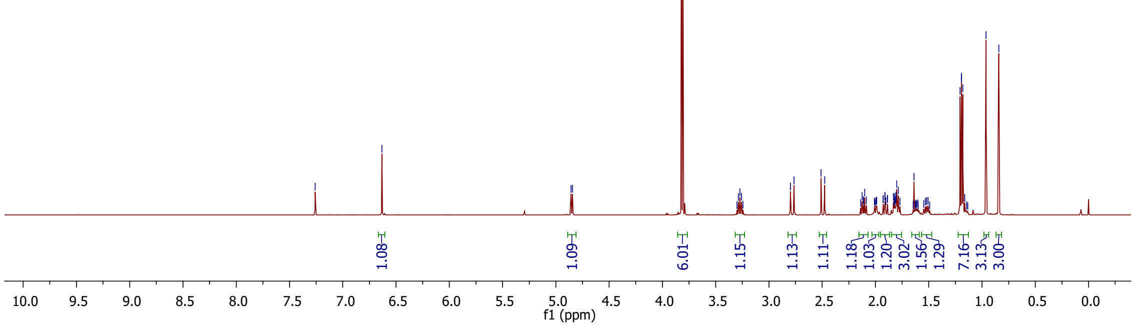

^{1}H NMR (500 MHz, $CDCl_3$), **7**

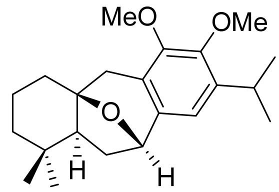

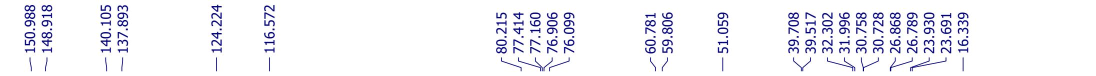

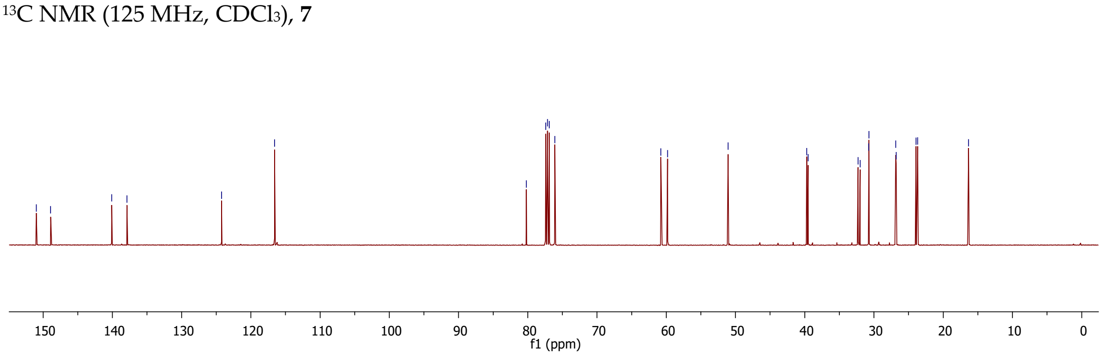

^{13}C NMR (125 MHz, $CDCl_3$), **7**

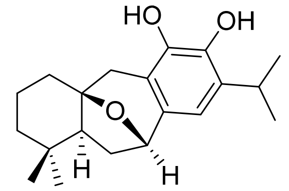

¹H NMR (400 MHz, $CDCl_3$), **1**

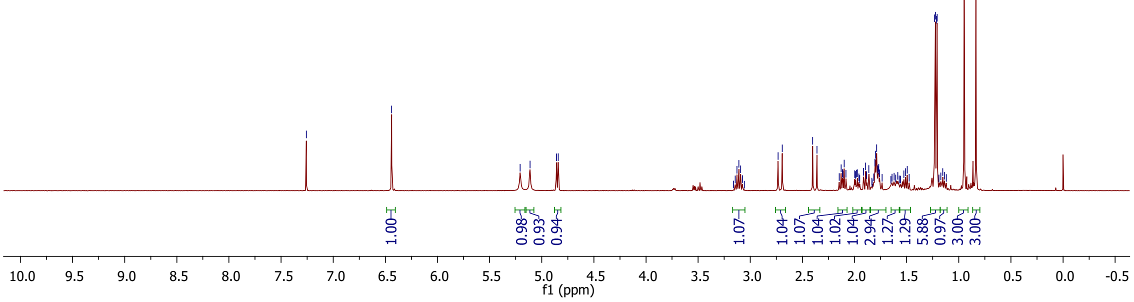

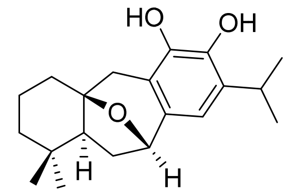

¹³C NMR (100 MHz, $CDCl_3$), **1**

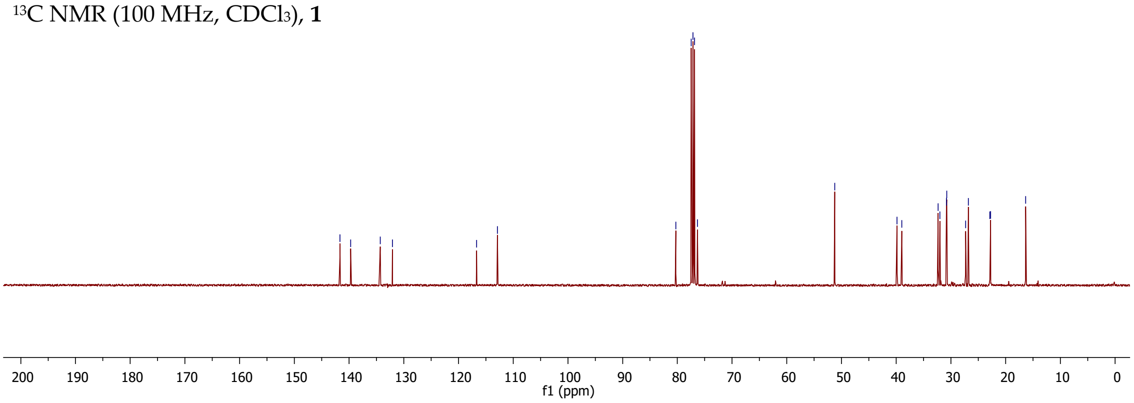

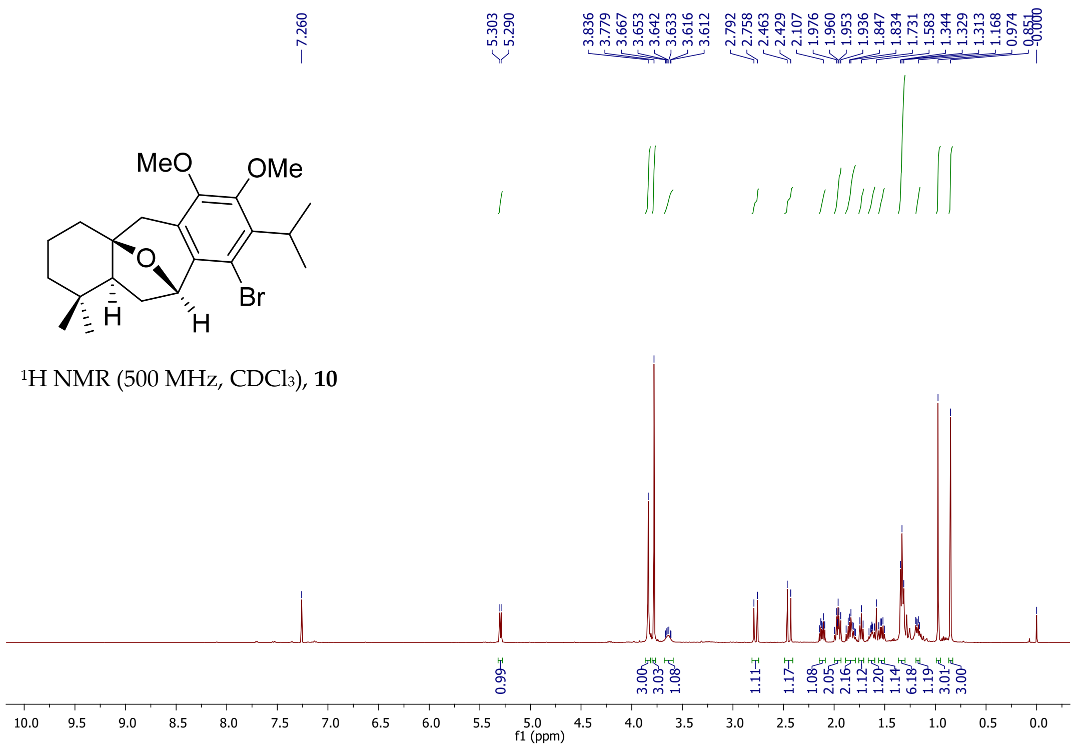

^{1}H NMR (500 MHz, CDCl$_3$), **10**

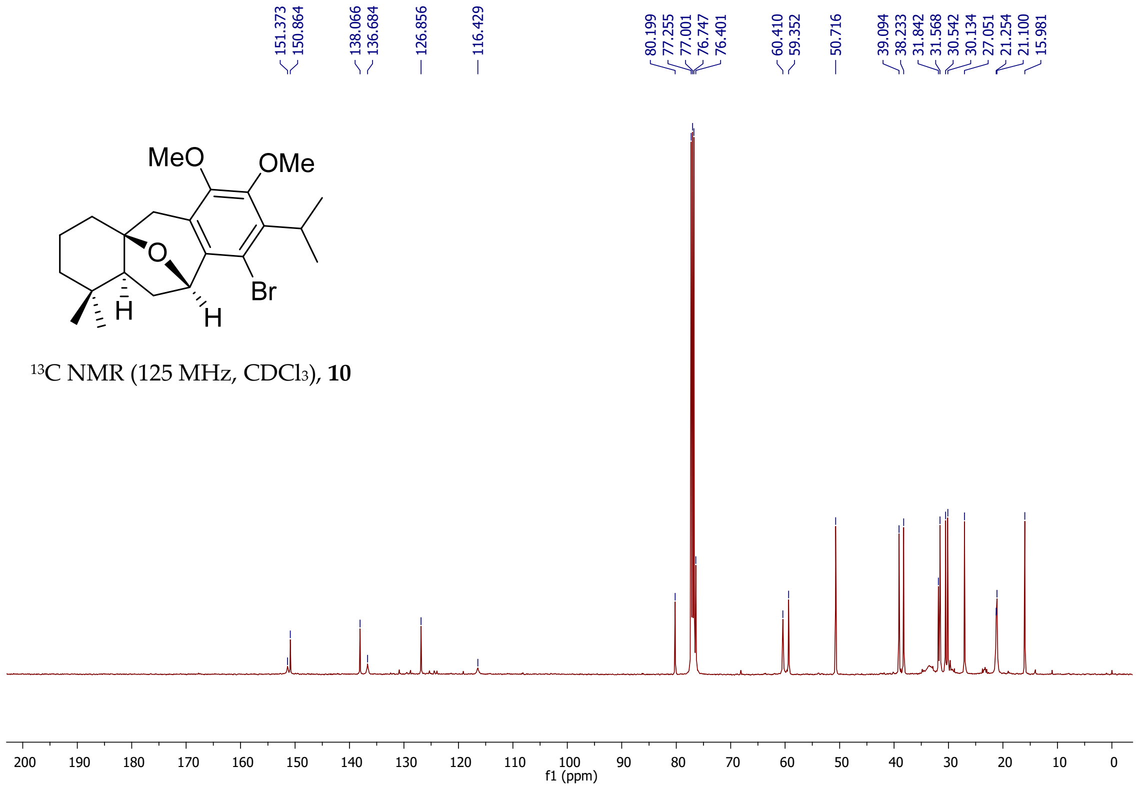

^{13}C NMR (125 MHz, CDCl$_3$), **10**

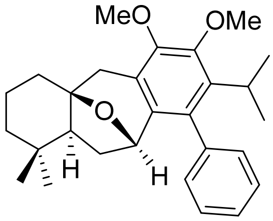

¹H NMR (400 MHz, $CDCl_3$), **11**

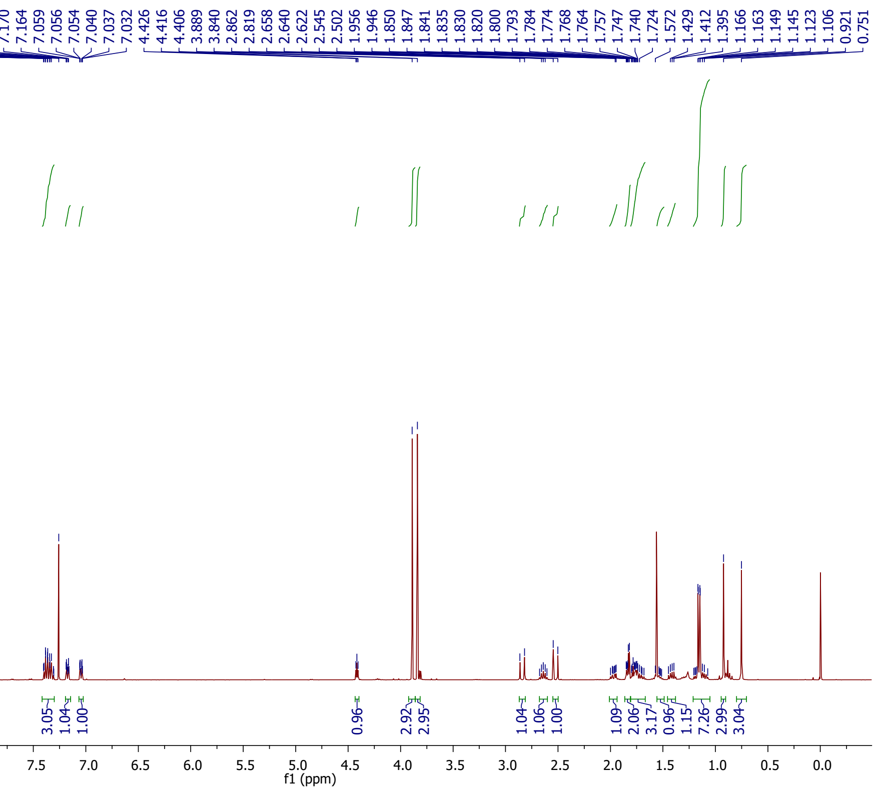

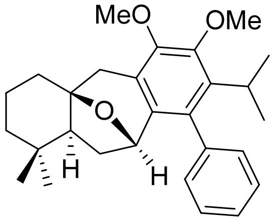

¹³C NMR (100 MHz, $CDCl_3$), **11**

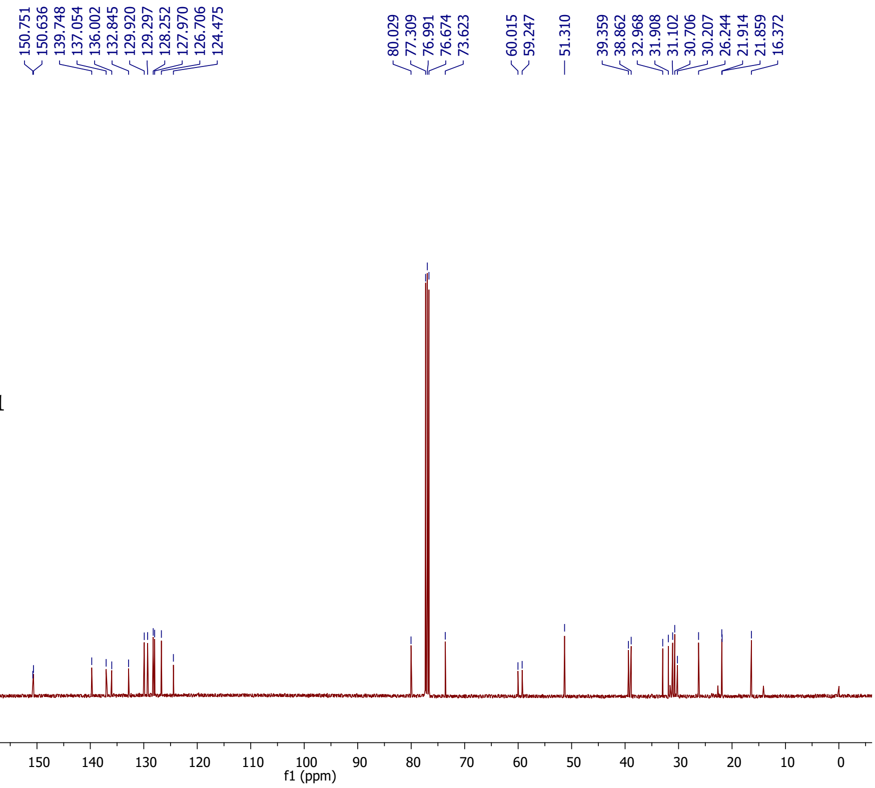

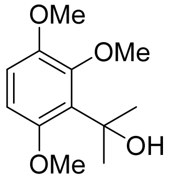

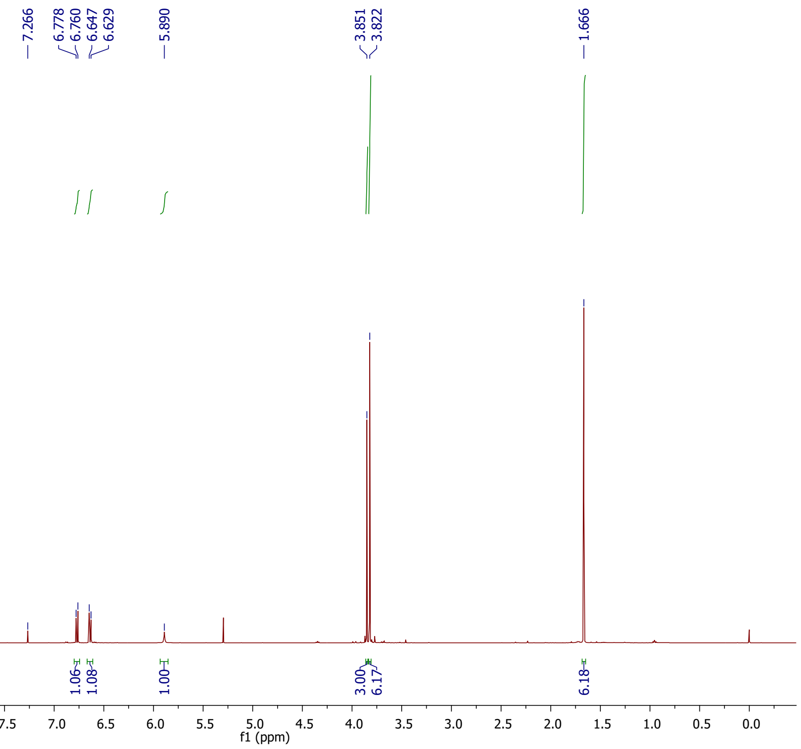

^{1}H NMR (500 MHz, $CDCl_3$), **41**

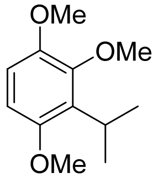

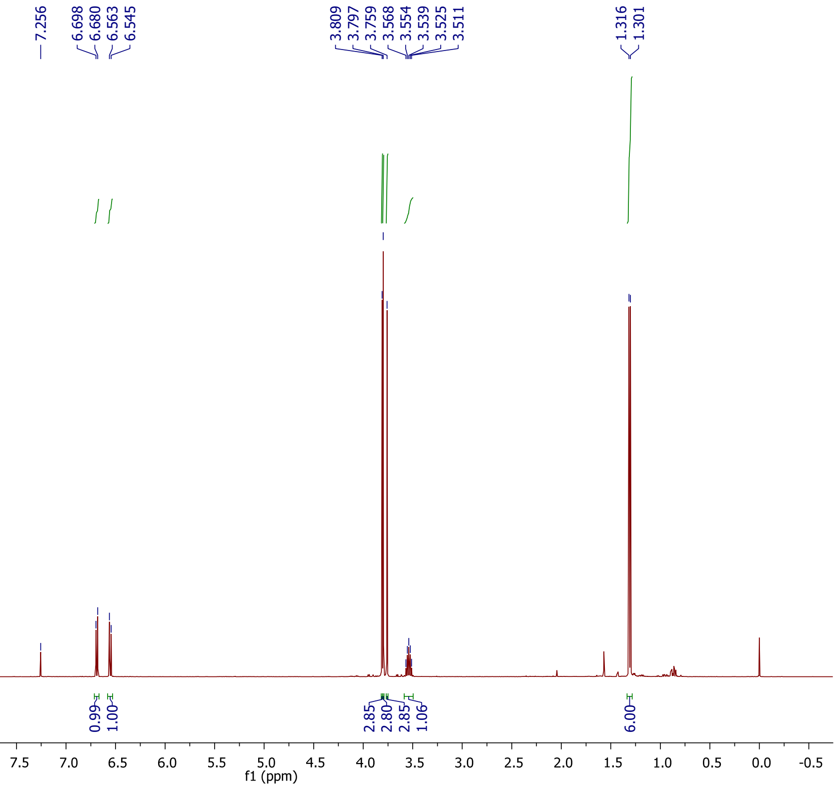

^{1}H NMR (500 MHz, $CDCl_3$), **21**

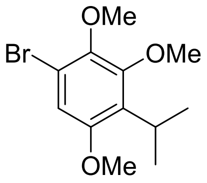

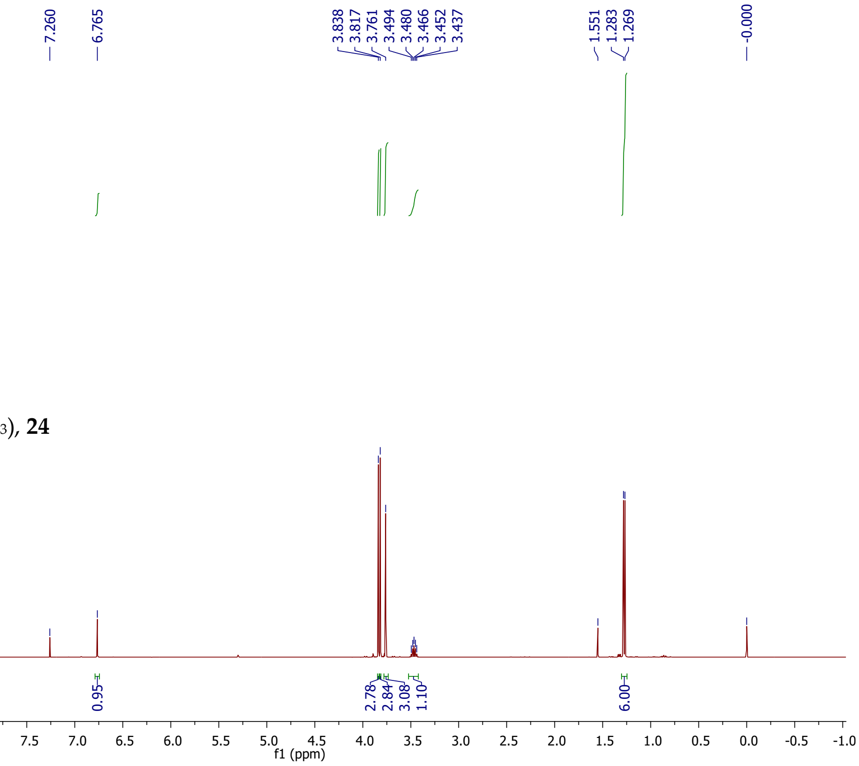

^{1}H NMR (500 MHz, $CDCl_3$), **24**

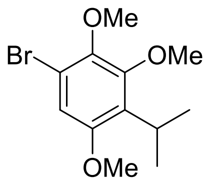

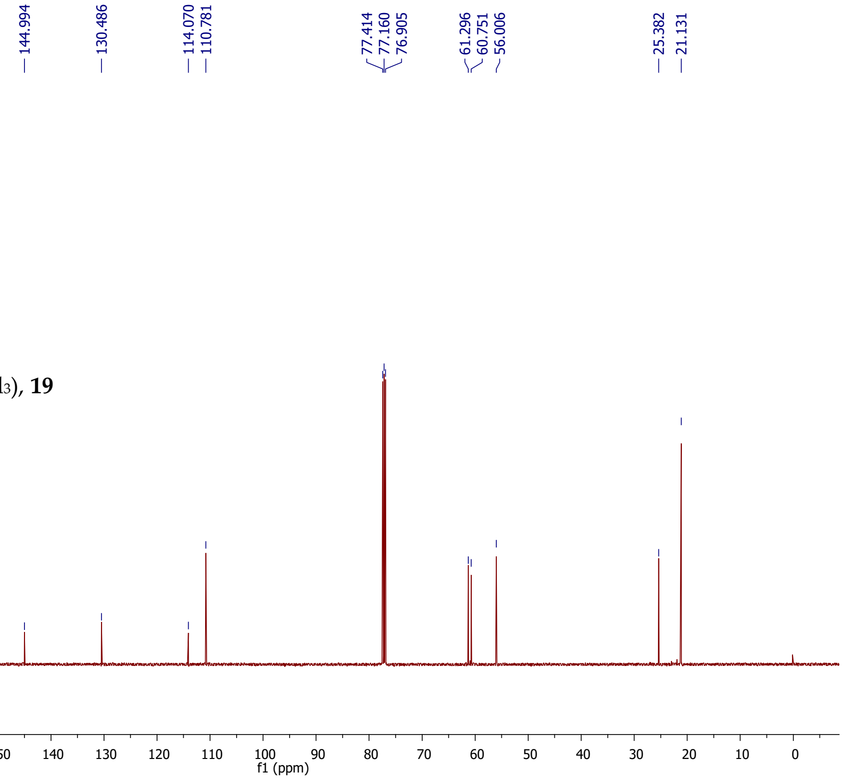

^{13}C NMR (125 MHz, $CDCl_3$), **19**

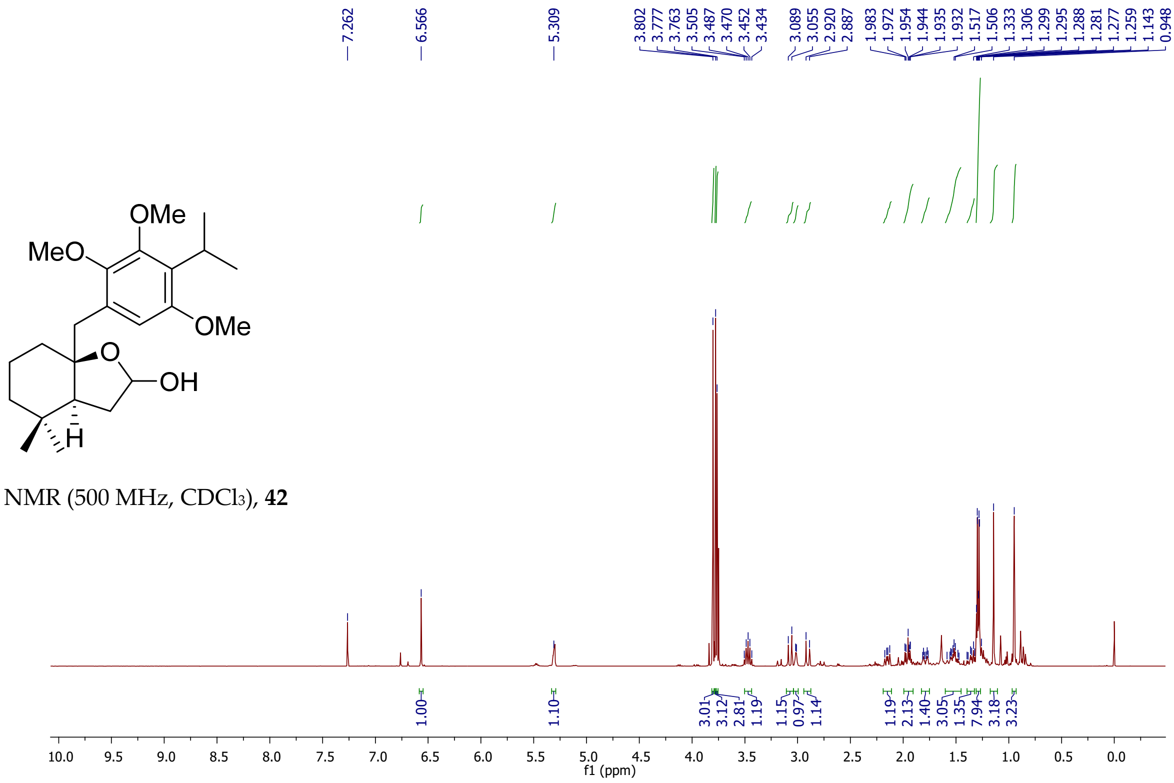

^{1}H NMR (500 MHz, $CDCl_3$), **42**

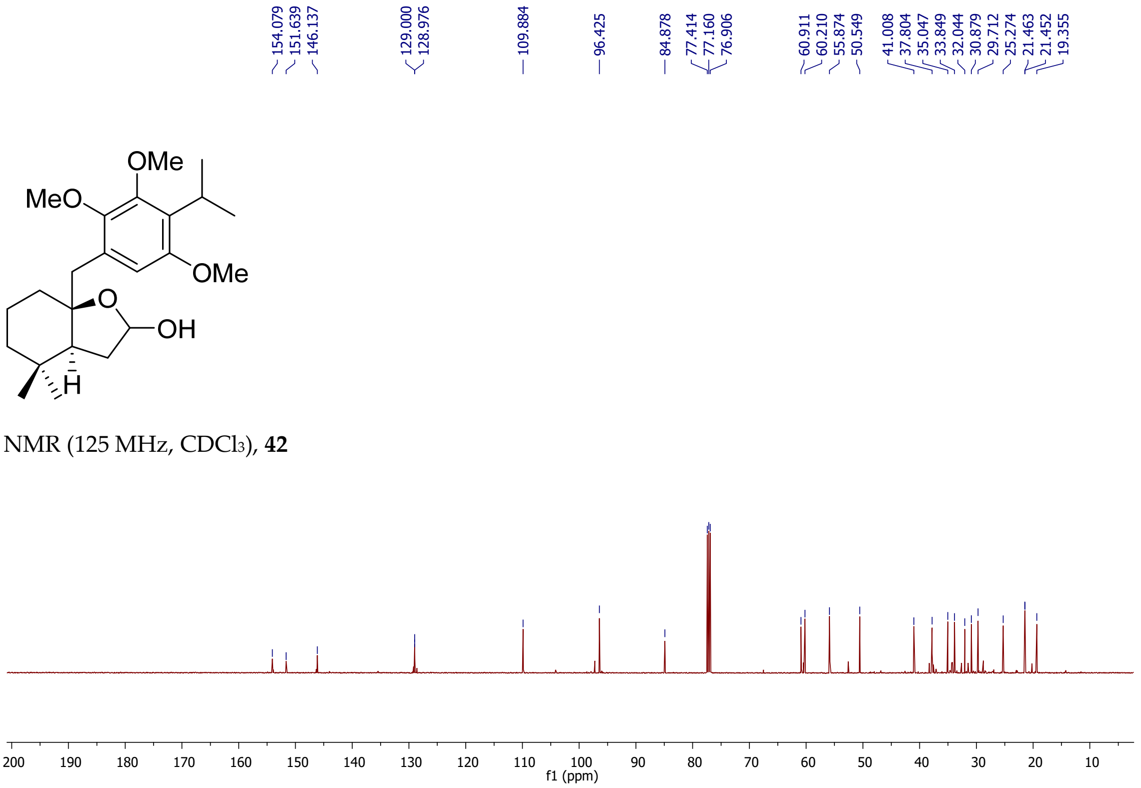

^{13}C NMR (125 MHz, $CDCl_3$), **42**

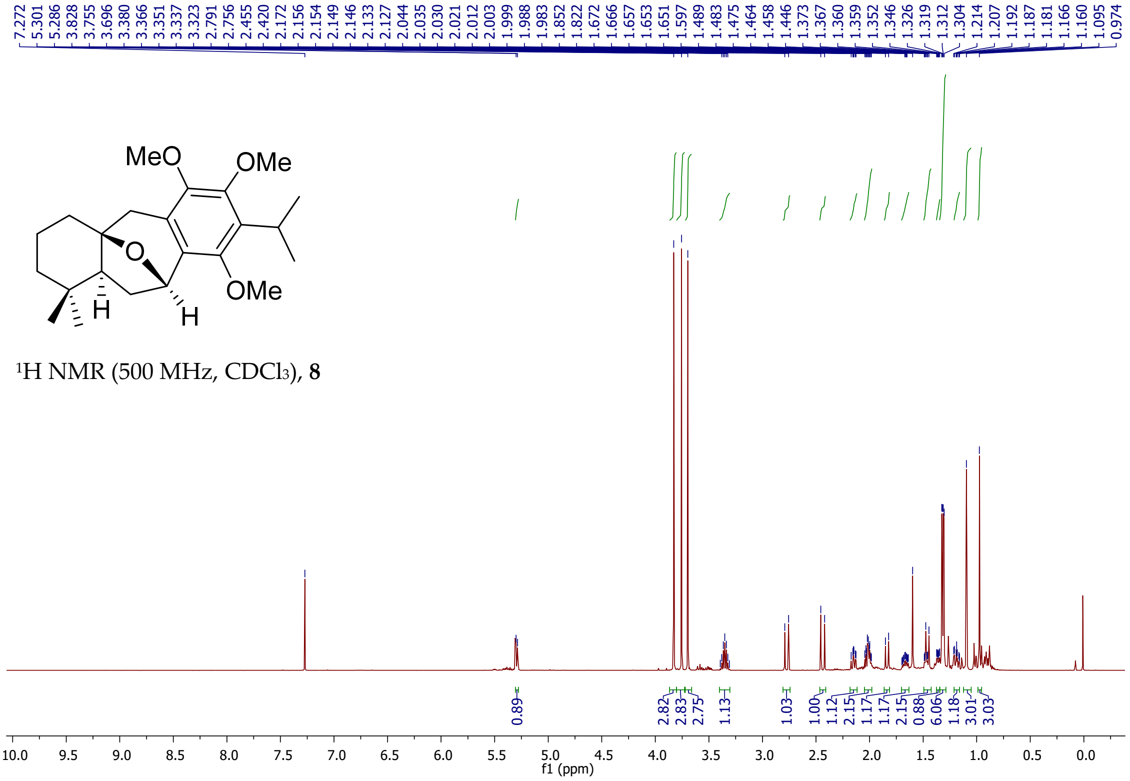

^{1}H NMR (500 MHz, $CDCl_3$), **8**

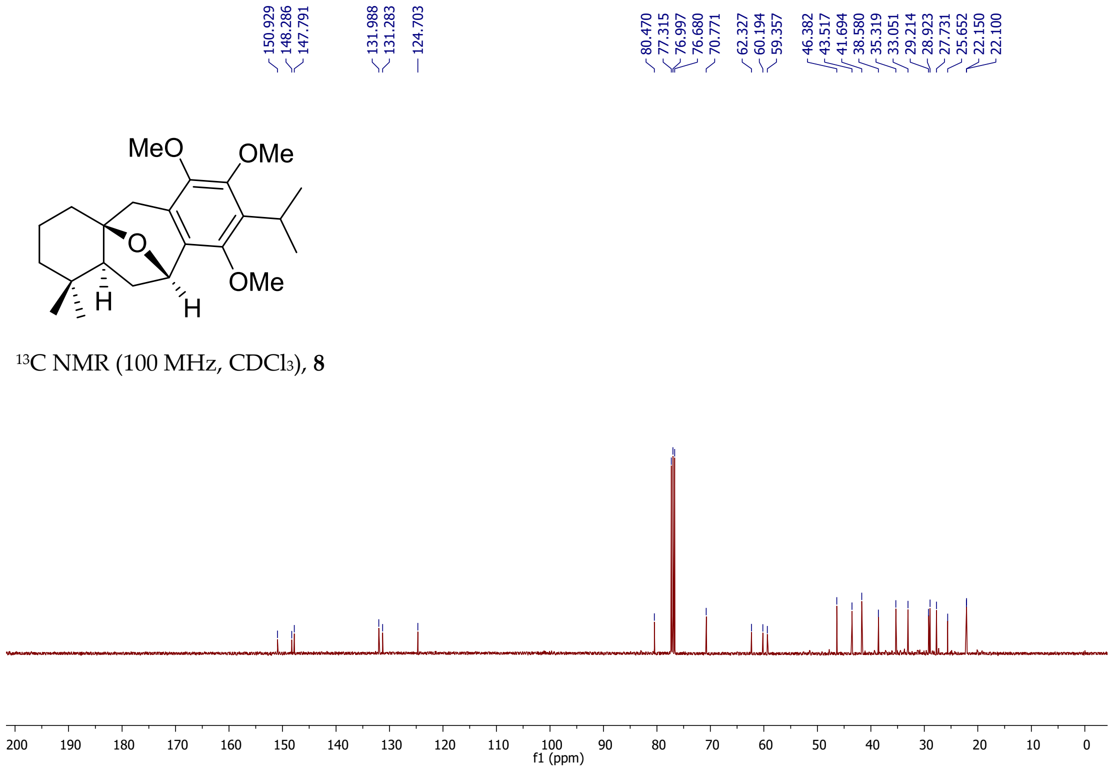

^{13}C NMR (100 MHz, $CDCl_3$), **8**

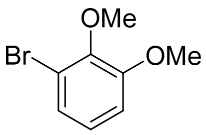

^{1}H NMR (500 MHz, CDCl3), **25**

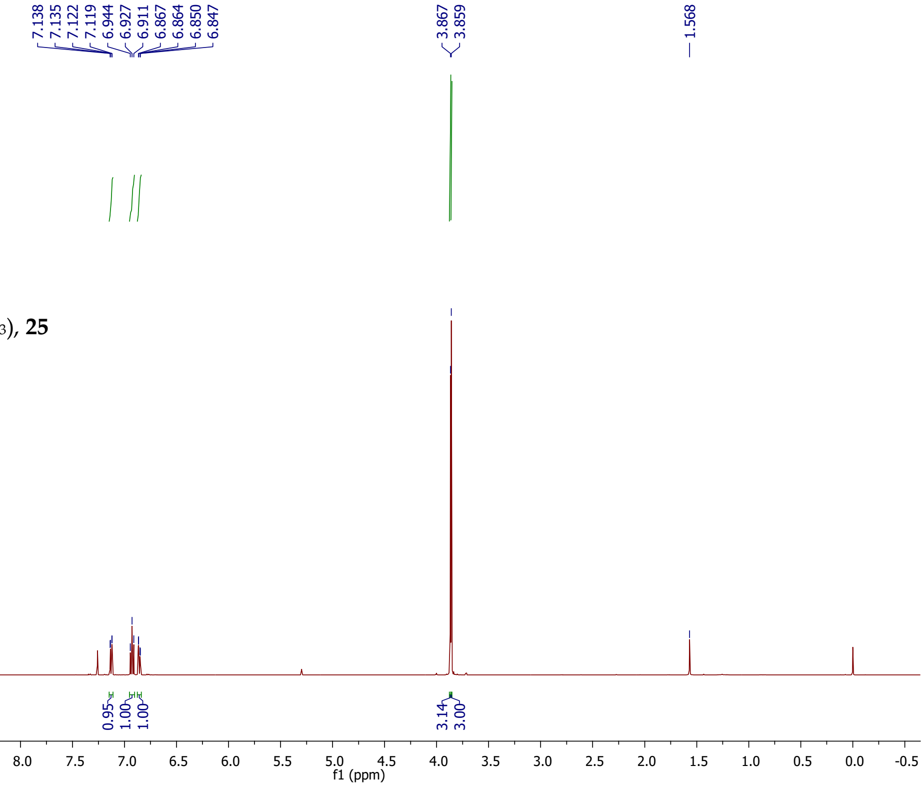

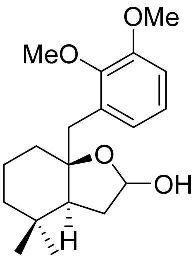

^{1}H NMR (500 MHz, CDCl3), **43**
Semi pure

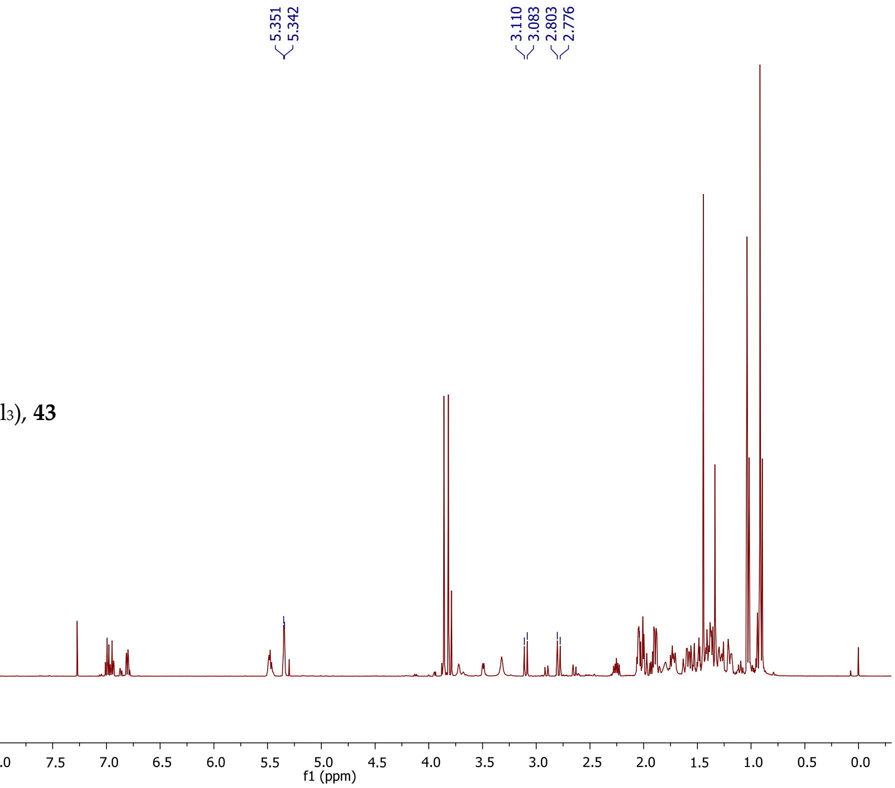

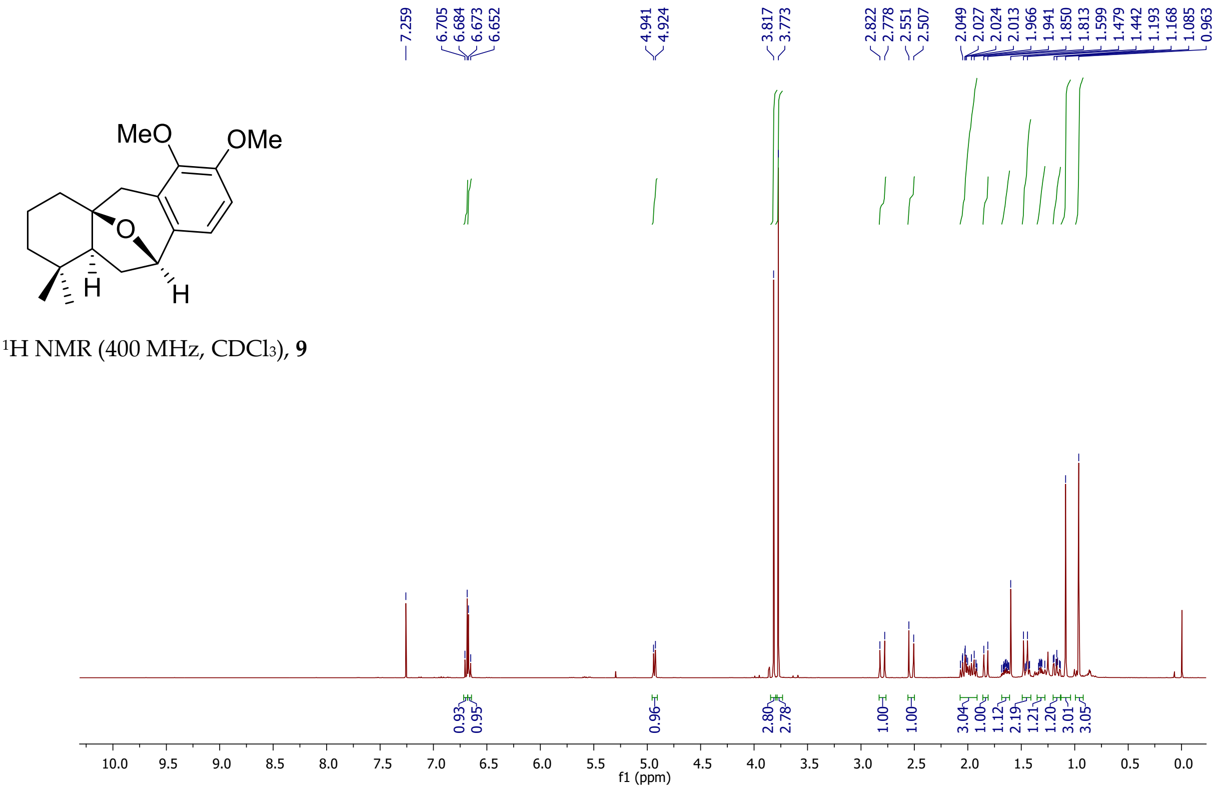

^{1}H NMR (400 MHz, $CDCl_3$), **9**

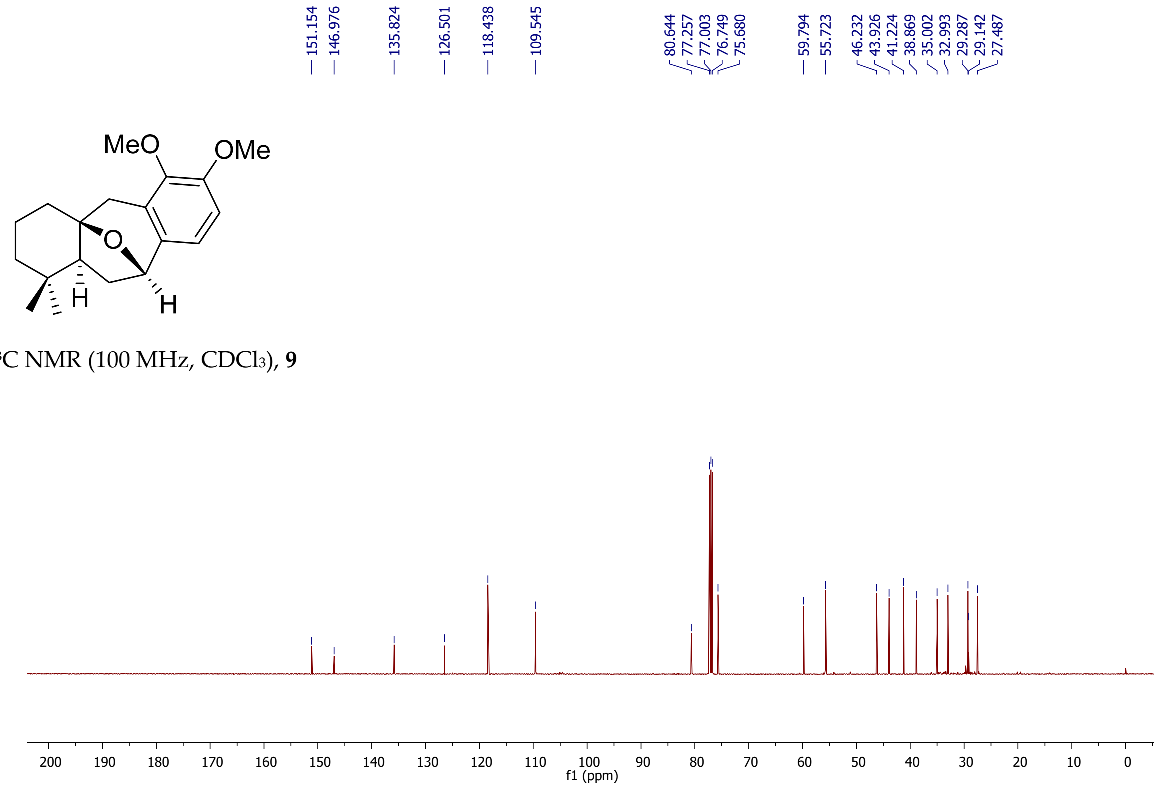

^{13}C NMR (100 MHz, $CDCl_3$), **9**

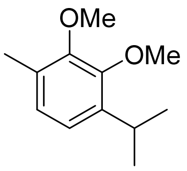

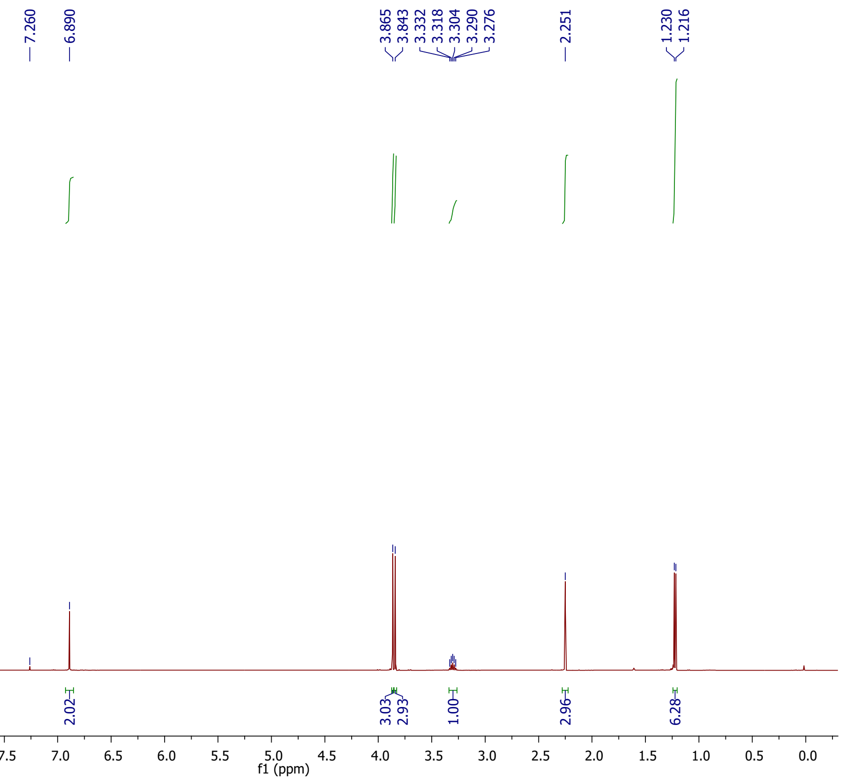

^{1}H NMR (500 MHz, CDCl3), **28**

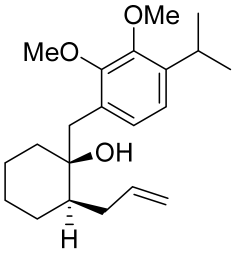

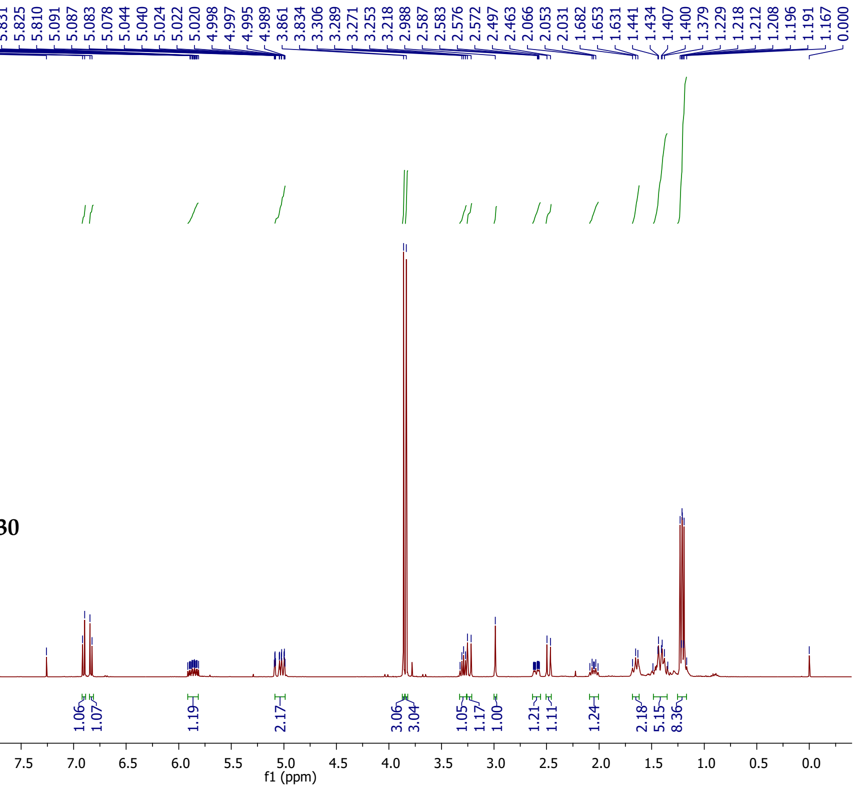

^{1}H NMR (400 MHz, CDCl3), **30**

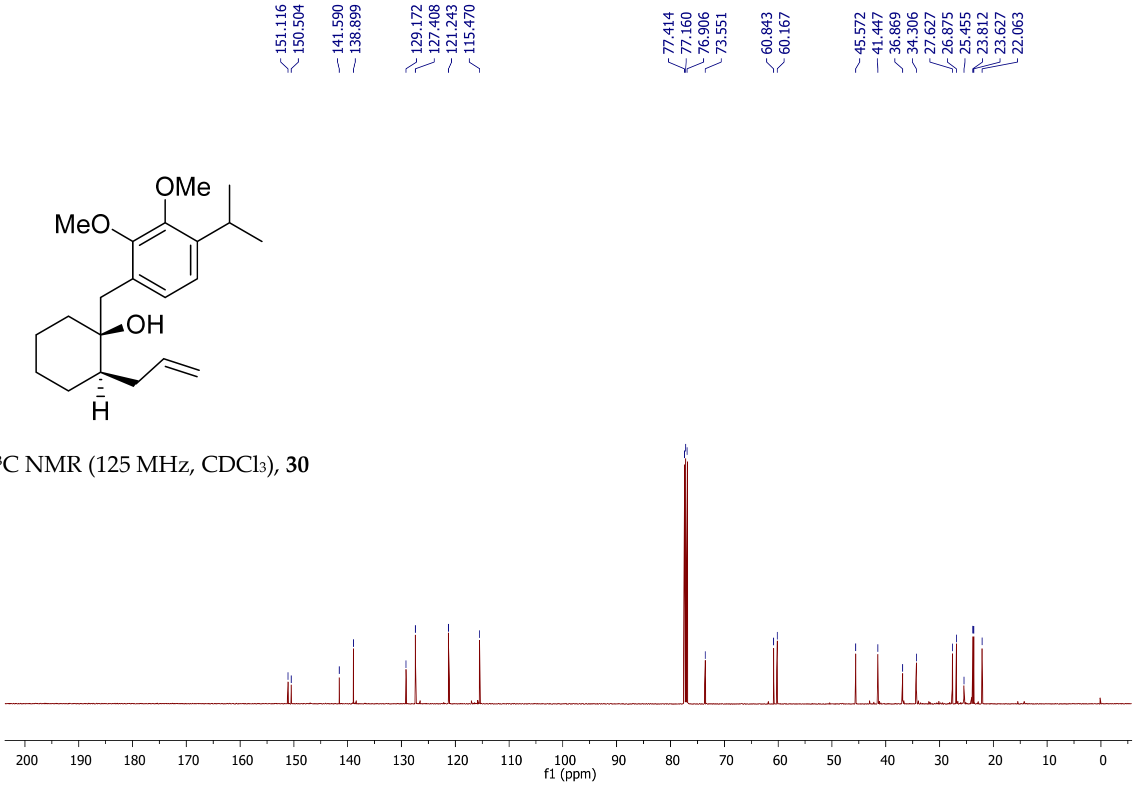

^{13}C NMR (125 MHz, $CDCl_3$), **30**

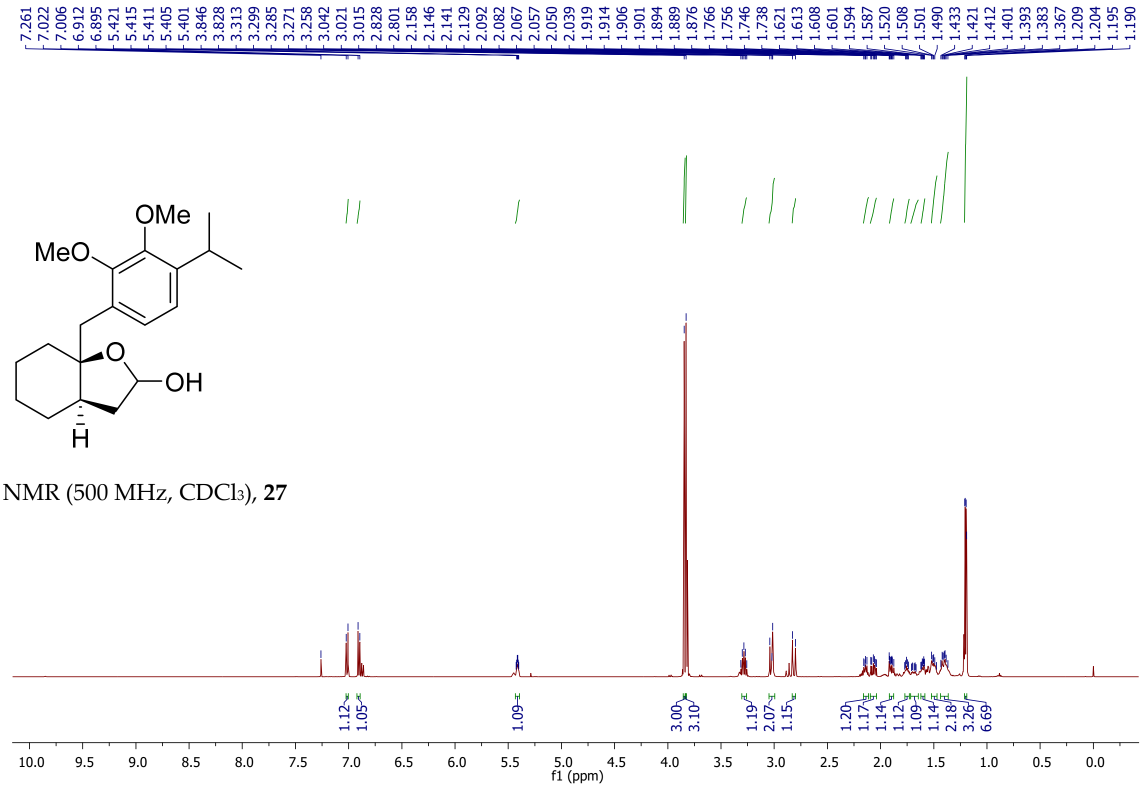

^{1}H NMR (500 MHz, $CDCl_3$), **27**

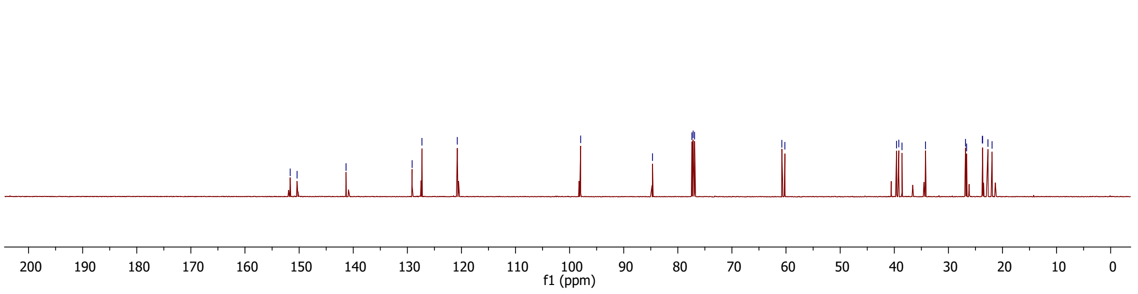

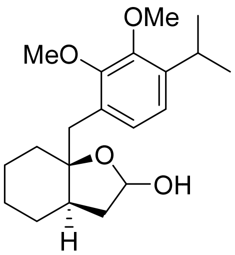

^{13}C NMR (125 MHz, CDCl3), **27**

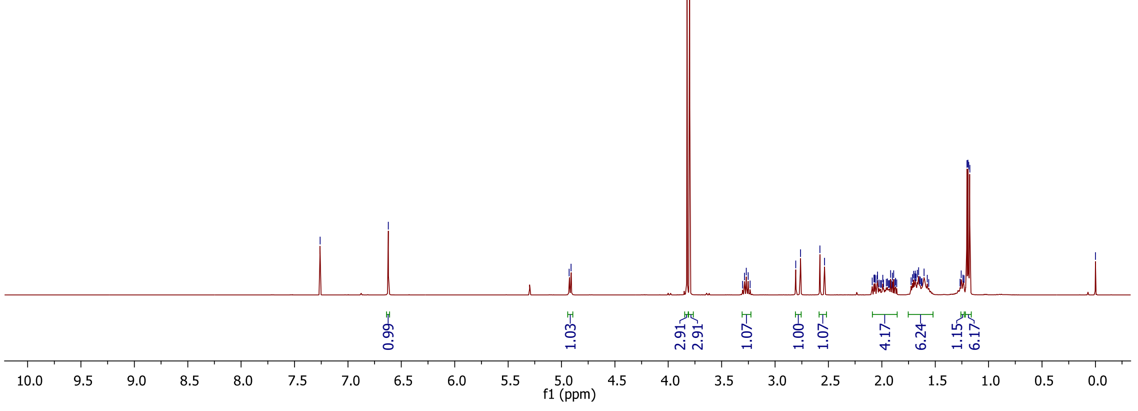

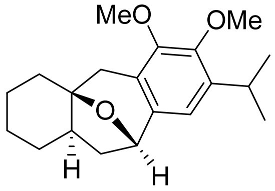

^{1}H NMR (400 MHz, CDCl3), 1**2**

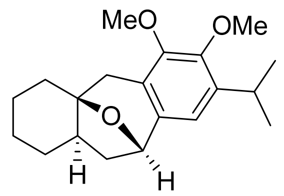

¹³C NMR (125 MHz, CDCl₃), 12

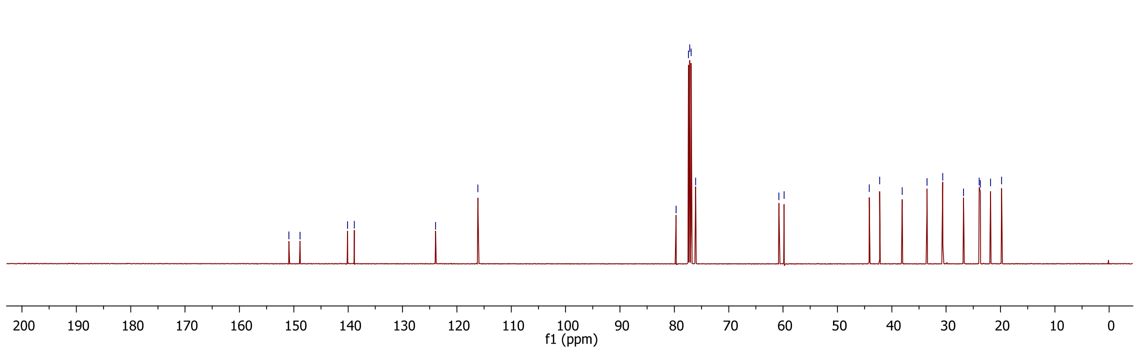

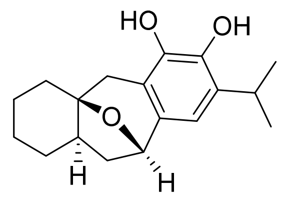

¹H NMR (400 MHz, CDCl₃), 13

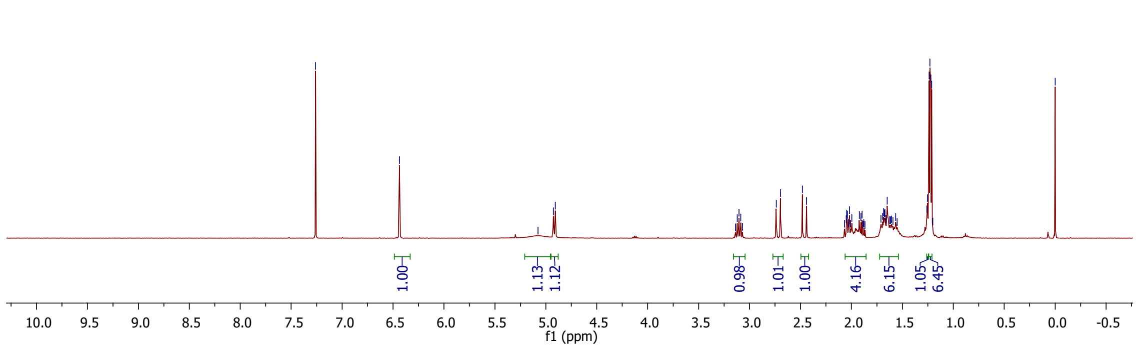

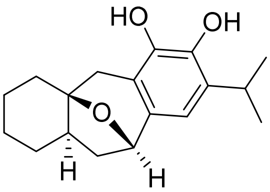

^{13}C NMR (100 MHz, $CDCl_3$), 13

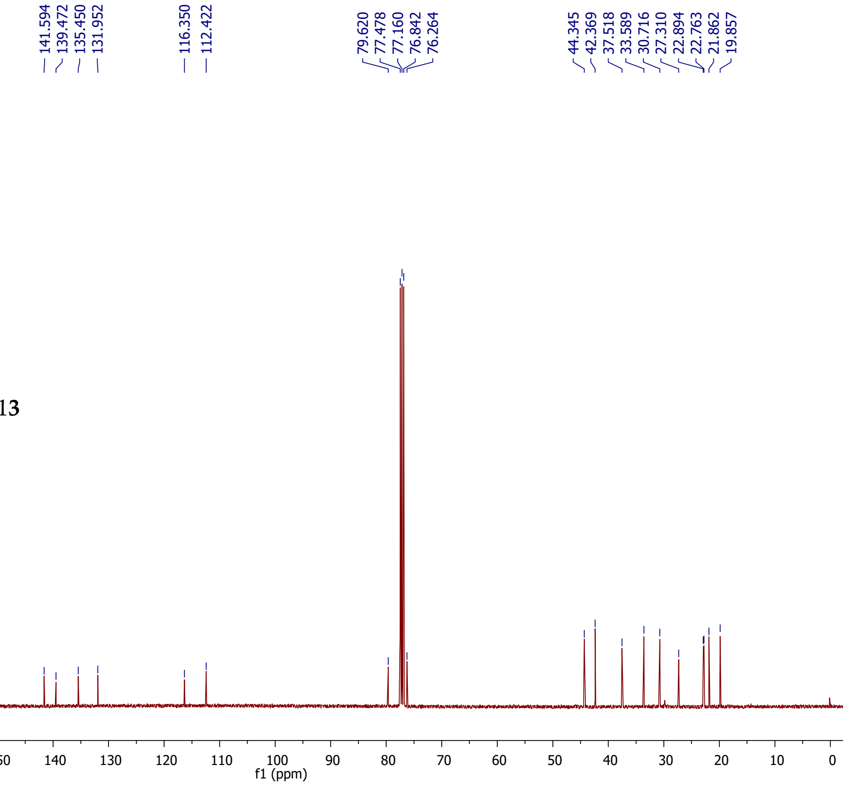

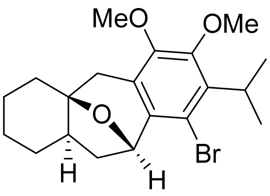

^{1}H NMR (500 MHz, $CDCl_3$), 14

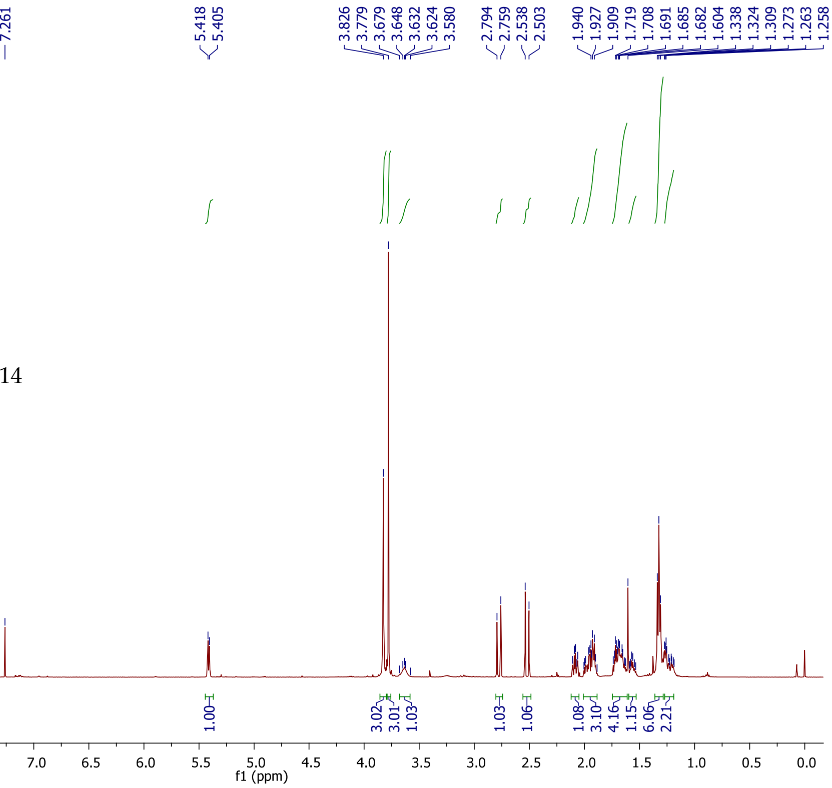

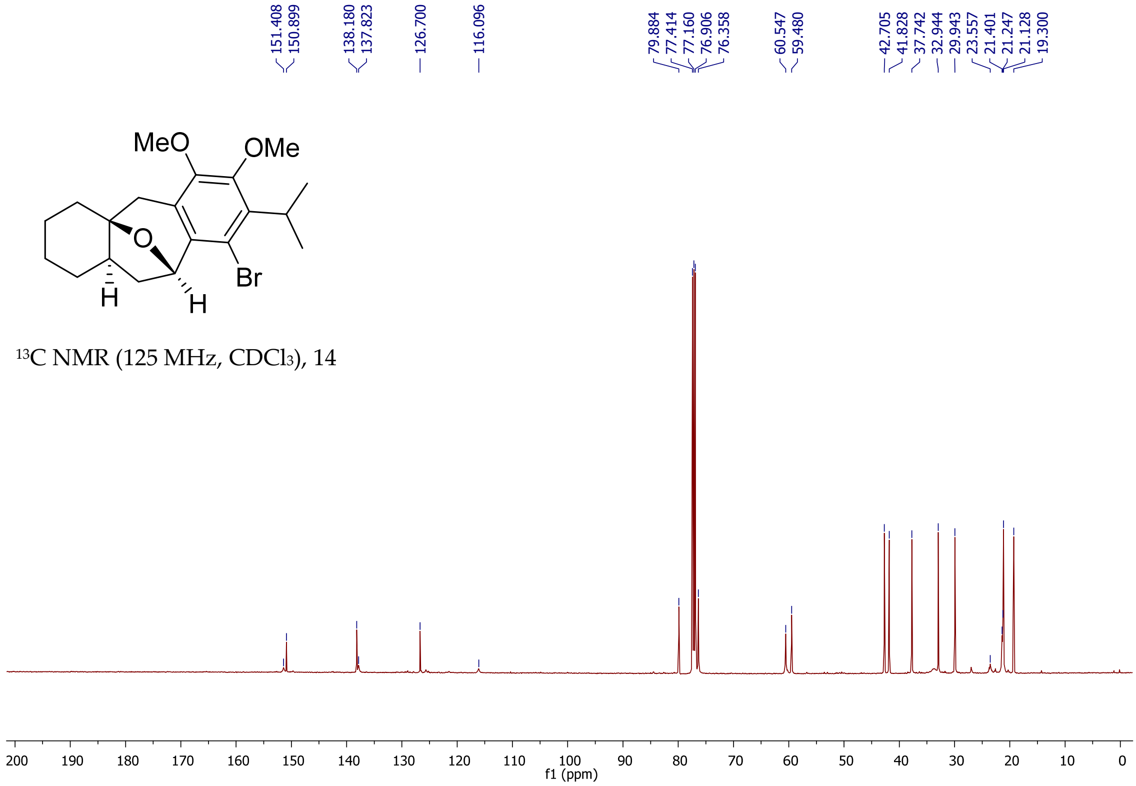

^{13}C NMR (125 MHz, $CDCl_3$), 14

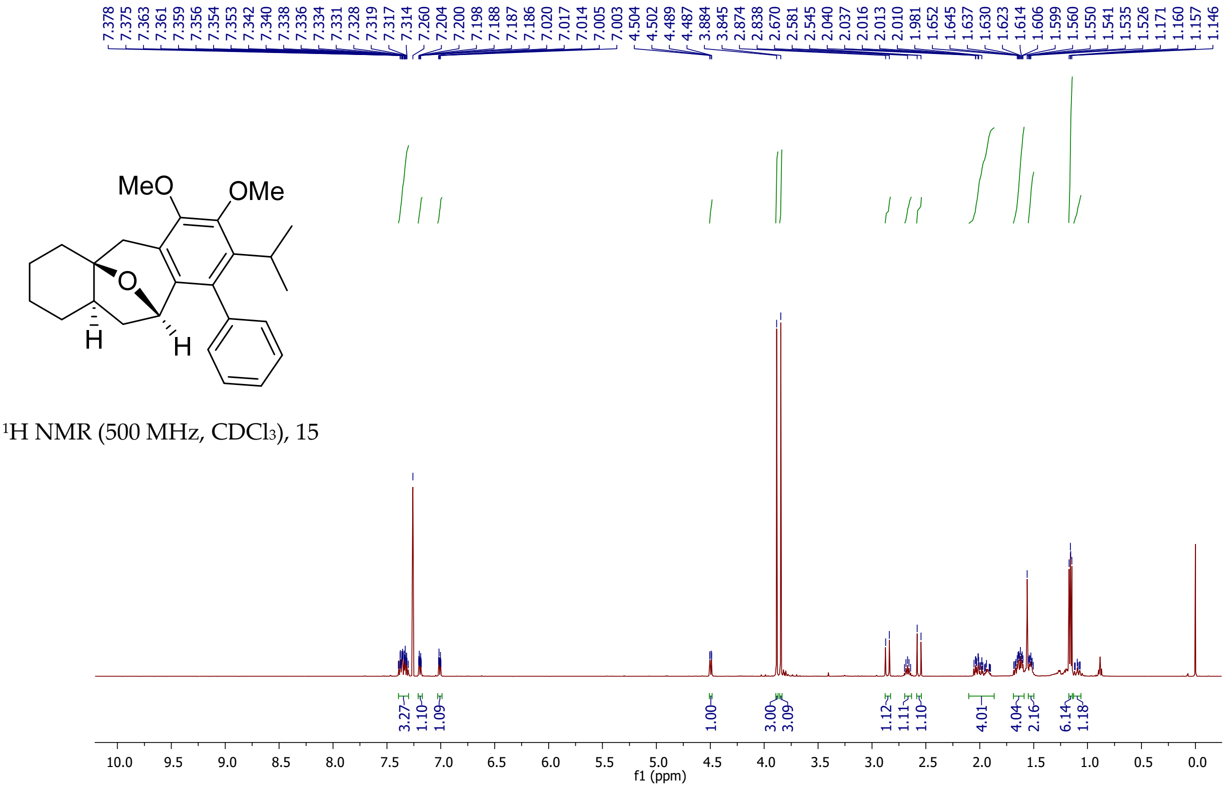

^{1}H NMR (500 MHz, $CDCl_3$), 15

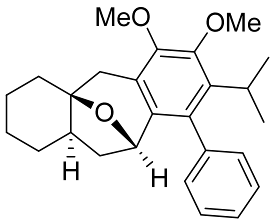

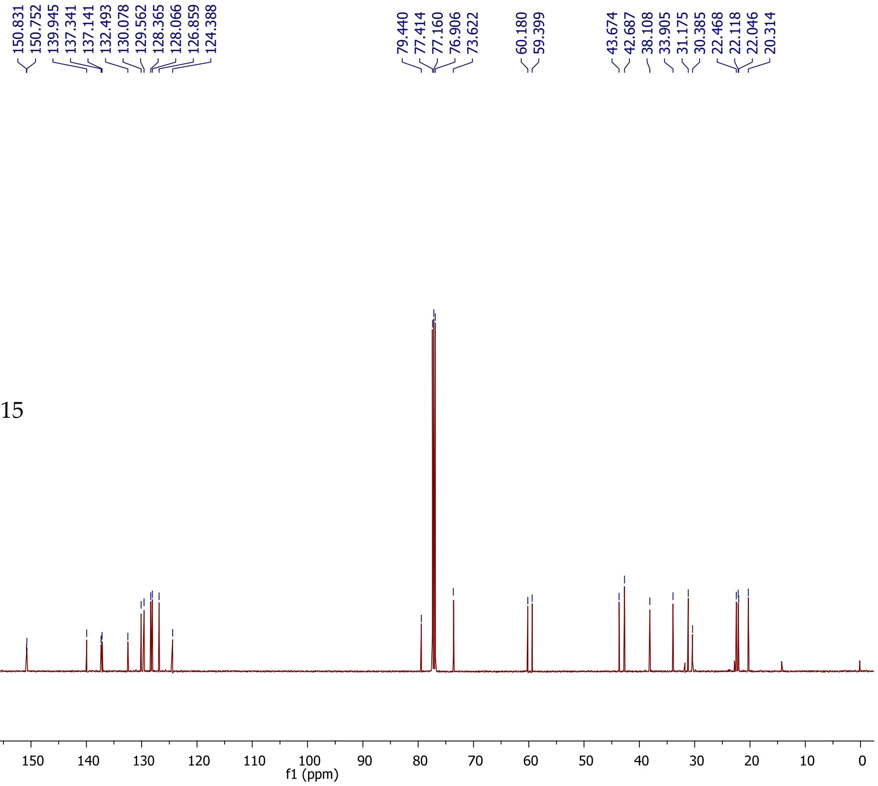

^{13}C NMR (125 MHz, $CDCl_3$), 15
